# Chemical Tools to Characterize the Coordination Chemistry of Radionuclides for Radiopharmaceutical Applications

**DOI:** 10.1021/acs.chemrev.5c00641

**Published:** 2025-10-28

**Authors:** Eszter Boros, Peter Comba, Jonathan W. Engle, Charlene Harriswangler, Suzanne E. Lapi, Jason S. Lewis, Simona Mastroianni, Liviu M. Mirica, Carlos Platas-Iglesias, Caterina F. Ramogida, Raphaël Tripier, Marianna Tosato

**Affiliations:** † Department of Chemistry, 5228 University of Wisconsin—Madison, Madison, Wisconsin 53706, United States; ‡ Universität Heidelberg, Anorganisch Chemisches Institut and Interdisciplinary Center for Scientific Computing, 69120 Heidelberg, Germany; § Department of Medical Physics, 5228 University of Wisconsin—Madison, 1111 Highland Avenue, Madison, Wisconsin 53705, United States; ∥ CICA - Centro Interdisciplinar de Química e Bioloxía and Departamento de Química, 16737 Universidade da Coruña, Campus da Zapateira-Rúa da Fraga 10, 15008 A Coruña, Spain; ⊥ Department of Radiology, University of Alabama at Birmingham, Birmingham, Alabama 35294, United States; # Molecular Pharmacology Program, 5803 Memorial Sloan Kettering Cancer Center, New York, New York 10065, United States; g Department of Chemistry, 1763 Simon Fraser University, 8888 University Drive, V5A 1S6 Burnaby, British Columbia, Canada; h Life Sciences Division, TRIUMF, 4004 Wesbrook Mall, V6T 2A3 Vancouver, British Columbia, Canada; i Department of Chemistry, The Neuroscience Program, Beckman Institute for Advanced Science and Technology, Carle Illinois College of Medicine, Department of Bioengineering, Carle Woese Institute for Genomic Biology, 14589 University of Illinois Urbana−Champaign, Urbana, Illinois 61801, United States; j Univ Brest, UMR CNRS 6521 CEMCA, 6 Avenue Victor Le Gorgeu, 29200 Brest, France; k Radiopharmaceutical Chemistry Laboratory, Nuclear Medicine Unit, AUSL-IRCCS Reggio Emilia, 42123 Reggio Emilia, Italy

## Abstract

During the past decade, the advancement and approval of novel radiopharmaceuticals for clinical application has led to a resurgence of the field of radiochemistry and specifically the coordination chemistry of radionuclides. In addition to well established radionuclides, short-lived radioisotopes of other elements are becoming accessible using new isotope production methods, necessitating the development of coordination chemistry compatible with the aqueous chemistry of such elements under tracer level conditions. As radiochemistry with radioactive metal ions relevant for radiopharmaceuticals is conducted at the nano- to picomole scale, conventional chemical characterization techniques can generally not be applied. Therefore, careful consideration and interfacing of tracer-level compatible techniques and macroscopic characterization methods is required. This Review provides an in-depth survey of common, contemporary characterization strategies for the coordination chemistry of radionuclides, including case studies to demonstrate context and relevance for the prospective development of clinically translatable radiopharmaceuticals.

## Graphical abstract

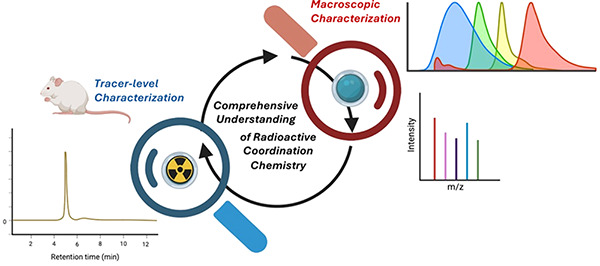

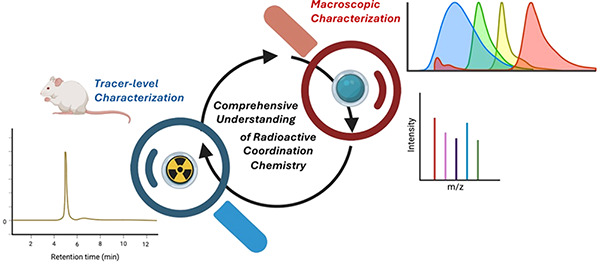

## 1 Introduction

During the mid-20th century, and briefly after the second world war, nuclear chemists and radiochemists were well represented in most chemistry departments. The unique blend of nuclear physics, separation science, and solution chemistry was often best understood and developed by scientists who could be considered inorganic and analytical chemists in our modern era. The subsequent decades in nuclear chemistry and radiochemistry research were dominated by applications in nuclear power and nuclear fuel recycling; as a consequence, many radiochemistry and nuclear chemistry research groups are still part of nuclear engineering departments today. The first wide-ranging clinical success of a radiopharmaceutical was brought about by the advent of ^99m^Tc separation and coordination chemistry. Clever separation strategies were devised to immobilize the parent nuclide ^99^Mo as [^99^Mo]­MoO_4_^2–^ on an ion exchange chromatography column at Brookhaven National Laboratory, allowing for selective elution of [^99m^Tc]­TcO_4_^–^. This effort, led by Powell Richards,^1,2^ provided a gateway for the development of [^99m^Tc]­Tc-coordination complexes as single photon emission computed tomography (SPECT) agents. The rich redox chemistry of technetium represented a formidable challenge, which was solved in various creative ways by Alan Davison’s group by the formation of Tc^5+^-oxo chelates and the hexa-isonitrile organometallic complex [Tc­(CNR)_6_]^+^ (R = CH_2_CMe_2_OMe) later developed and employed as the cardiac imaging agent cardiolite/sestamibi (Figure 1).^3,4^ For several decades, reactor-produced radioisotopes dominated the field of nuclear medicine, with ^99m^Tc remaining a focal point of radiochemistry development and translation, which required extensive inorganic chemistry know-how.^5–9^ The lack of a stable, long-lived isotope of Tc combined with the differing redox properties of the heavier congener Re required critical assessment of the corresponding organometallic and coordination complexes formed to validate their chemical homology. As a consequence, the discovery of a radiochemical advance required the careful correlation of macroscopic Re or Tc chemistry with tracer (<10^–8^ M) level ^99m^Tc chemistry to conclusively affirm the identity of the corresponding solution species.^10^

**Figure 1:**
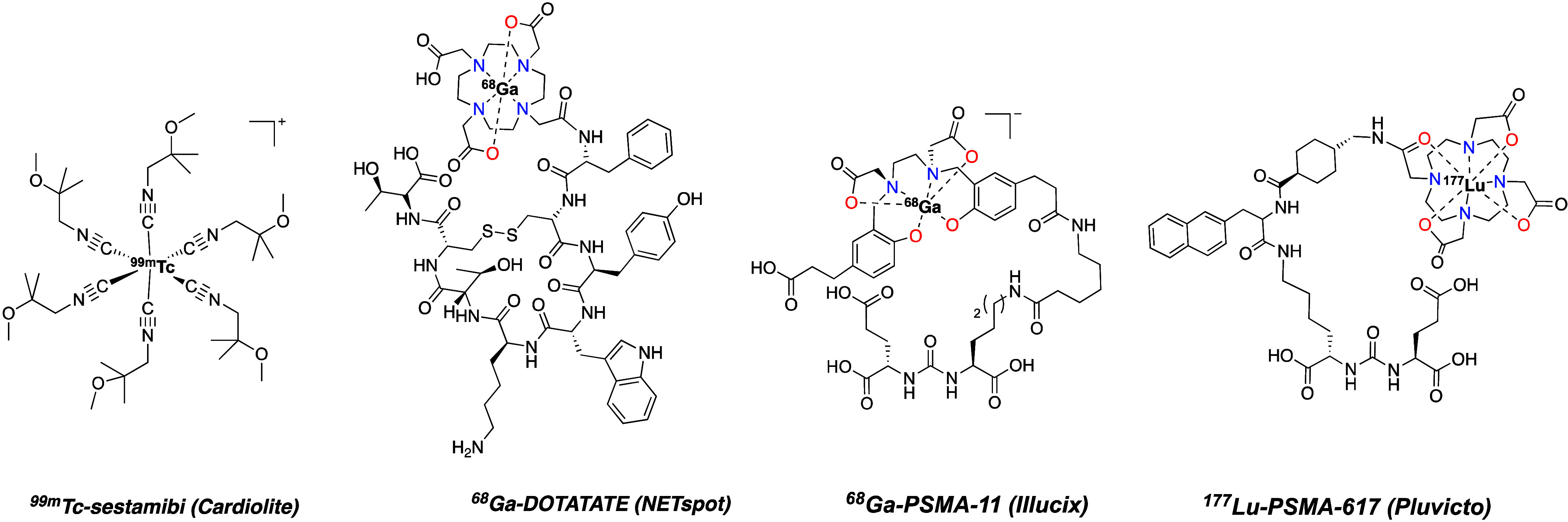
Chemical structures of several contemporary, clinically approved radiopharmaceuticals comprised of coordination compounds.

Since the early 2000s, interest in short-lived nuclides and their potential application in nuclear medicine has steadily grown and further accelerated due to the globally increasing availability and accessibility of small, low-energy cyclotrons. This has not only increased access to the positron emission tomography (PET) isotopes ^18^F and ^11^C, but also reinvigorated interest in short-lived radioactive isotopes that require coordination chemistry approaches for their incorporation into targeted radiopharmaceuticals. Many of these radionuclides, such as ^177^Lu, ^68^Ga, and ^89^Zr, have no relevant redox behavior in aqueous media and readily form coordination complexes with a host of chelators. The wide-ranging dissemination of these isotopes beyond the few expert radiochemistry laboratories has helped to significantly accelerate the preclinical, and subsequently clinical development of radiopharmaceuticals incorporating many nonstandard radioisotopes. Specifically, the incorporation of developmental radiochemistry laboratories within medical research institutes and clinical radiopharmacies has played a significant role in translating recently Food and Drug Administration (FDA)-approved radiopharmaceuticals such as [^68^Ga]­Ga-DOTATATE (NETSPOT, approved in 2016), [^177^Lu]­Lu-DOTATATE (Lutathera, approved in 2018), [^68^Ga]­Ga-PSMA-11 (Illucix, approved in 2020) and [^177^Lu]­Lu-PSMA-617 (Pluvicto, approved in 2022, Figure 1). Indeed, this work was made possible by several teams including Mäcke and co-workers,^11–15^ who employed a coordination-chemistry-based approach to identify appropriate radiochelate conjugates that could be functionalized to produce efficient tumor localization in animal models while minimizing loss of the radiometal cargo in circulation. As such, several early reports on clinically translated somatostatin targeting conjugates included the study of model complexes with standard chemical characterization techniques including X-ray crystallography, providing insight into the distinct coordinative preferences of small ionic radius ions such as Ga^3+^ and In^3+^ when compared with rare earth ions Lu^3+^ and Y^3+^.^11,16^ Another pertinent example is the recently clinically translated [^64/67^Cu]­Cu-SarTATE, which was developed by Donnelly and co-workers,^17–21^ who recognized the potential of Sargeson’s sarcophagine chelators for the low-temperature, inert chelation of copper radioisotopes and probed the permutation of functionalization strategies to identify means to append targeting peptides for disease-specific delivery of radioactive copper chelates.^18^

These recent successful clinical translations and recent FDA approval of a number of radiochelates have significantly contributed to a surge in interest in radiochelation chemistry in the past decade. An increased involvement of scientists in radiochemical and radiopharmaceutical development with limited organic and inorganic chemistry expertise has led to chemical characterization playing a diminishing role. While validated chelation chemistry may not require elaborate chemical analysis to affirm a known metal-ion binding mechanism, the development of new radiochemical methods and molecular constructs must be conducted with appropriate rigor. Specifically, as new radioisotopes and radiometals with great diagnostic or therapeutic potential become accessible, it is essential to adhere to validated macroscopic and tracer-level characterization methods that provide a holistic picture of new (radio)­chemical entities prior to further preclinical development and eventual clinical translation. One of the core challenges of tracer-level radiochelation chemistry is scale, which renders most common chemical characterization methods insufficiently sensitive for analysis. Therefore, the thorough characterization of macroscopic, nonradioactive analogues or congeners is required prior to radiochemical experimentation. Subsequent radiochemical experimentation must take into consideration not only the pre-established behavior of the target compound under radioanalytical characterization conditions but also the effect of high dilution on kinetics and thermodynamics, which can be difficult to mimic on a macroscopic scale.

The goal of this Review is to provide a concise survey of essential chemical characterization techniques for the study of the coordination chemistry of established and emerging radiometal/-metalloid isotopes in the context of radiopharmaceutical development. Analytical techniques for the macroscopic characterization of radiochelation precursors and analogues are not only essential to affirm the identity of new chemical structures but can readily infer or help rationalize radiolabeling performance and *in vitro* and *in vivo* inertness of radiochelates. Furthermore, we survey readily accessible techniques and describe commonly employed strategies and conditions for experimentation with a diverse array of radionuclides of interest *in vitro* and *in vivo*. Taken together, we aim to provide a valuable resource for both novice and seasoned radiochemists with diverse chemical backgrounds.

## 2 Radionuclide Production

### 2.1 General Considerations of Radionuclide Production

Radiometals are typically produced via charged particle bombardment in accelerators, in reactors via fission or neutron capture reactions, or via photonuclear reactions. The production of radionuclides via charged particle reactions can lead to products with high molar activity (amount of radioactivity per unit mass) as the chemical element of the product is different from the target material and can thus be chemically separated. Examples of nuclear reactions leading to medically relevant isotopes are ^64^Ni­(*p,n*)^64^Cu, ^89^Y­(*p,n*)^89^Zr, and ^205^Tl­(*p,3n*)^203^Pb.^22–25^ In some cases, the target material is monoisotopic (e.g., naturally occurring Y is 100% ^89^Y) and thus enables economical production. In other cases, enriched material must be employed (e.g., ^64^Ni) and target material recycling becomes essential to ensure cost-effectiveness.

Reactor-based radioisotope production can be used to induce fission products including the generator system ^99^Mo/^99m^Tc where the ^99^Mo parent radioisotope is produced from fissionable material and decays into SPECT isotope ^99m^Tc. Neutrons produced from fission events can also be used in neutron capture reactions to produce therapeutic isotopes, including ^177^Lu and ^90^Y.^26^ While the majority of isotopes produced by neutron capture are in low molar activity as the product is the same element as the target material and cannot be chemically separated, for example the production of ^90^Y via ^89^Y­(n,γ)^90^Y, radionuclides can also be made in high molar activity by taking advantage of indirect routes, for example, ^176^Yb­(n,γ)^177^Yb → ^177^Lu, where the final product (^177^Lu) can be separated from the Yb target material. As is the case with other routes, natural or isotope-enriched targets may be employed. Photonuclear production routes typically make use of electron accelerators that convert high energy electrons into photons, which can be used to induce nuclear reactions (Table 1). While less widely employed than other accelerators and reactor-based methods, this route shows significant promise for certain isotopes including ^67^Cu, ^47^Sc, and ^225^Ac.^27–32^

**Table 1:** Selected Metal Isotopes and Chemical Form as Precursors

**Nuclide**	**Half-life (*t*_1/2_)^349^**	**Common chemical precursor (salt form)**	**Selected references**
^43^Sc, ^44^Sc, ^47^Sc	3.891 h, 4.042 h, 3.349 d	[^43^Sc]Sc-chloride ([^43^Sc]ScCl_3_), [^44^Sc]Sc-chloride ([^44^Sc]ScCl_3_), [^47^Sc]Sc-chloride ([^47^Sc]ScCl_3_)	^35–38^
^45^Ti	3.075 h	[^45^Ti]Ti-chloride ([^45^Ti]TiCl_4_), [^45^Ti]Ti-hydroxide ([^45^Ti]TiOH_*x*_)	^39–41^
^55^Co, ^58m^Co	17.53 h, 9.10 h	[^55^Co]Co-chloride ([^55^Co]CoCl_2_), [^58m^Co]Co-chloride ([^58m^Co]CoCl_2_)	^42–44^
^60^Cu, ^61^Cu, ^62^Cu, ^64^Cu, ^67^Cu	23.7 min, 3.339 h, 9.67 min, 12.70 h, 61.83 h	[^60^Cu]Cu-chloride ([^60^Cu]CuCl_2_), [^61^Cu]Cu-chloride ([^61^Cu]CuCl_2_), [^62^Cu]Cu-glycine, [^64^Cu]Cu-chloride ([^64^Cu]CuCl_2_), [^67^Cu]Cu-chloride ([^67^Cu]CuCl_2_)	^45–51^
^67^Ga, ^68^Ga	3.262 d d, 67.71 min	[^67^Ga]Ga-citrate,[^68^Ga]Ga-chloride ([^68^Ga]GaCl_3_)	^52–54^
^86^Y, ^90^Y	14.74 h, 64.05 h	[^86^Y]Y-chloride ([^86^Y]YCl_3_), [^90^Y]Y-chloride ([^90^Y]YCl_3_)	^54–58^
^89^Zr	78.41 h	[^89^Zr]Zr-oxalate ([^89^Zr]Zr(ox)_2_), [^89^Zr]-chloride ([^89^Zr]ZrCl_4_)	^23^,^24^,^59^,^60^
^201^Tl	3.042 d	[^201^Tl]Tl-chloride ([^201^Tl]TlCl)	^61^
^99m^Tc	6.007 h	[^99m^Tc]Tc-pertechnetate ([^99m^Tc]TcO_4_^–^)	^62^
^111^In	2.805 d	[^111^In]In-chloride ([^111^In]InCl_3_)	^63^
^117m^Sn	14.00 d	[^117m^Sn]Sn-chloride ([^117m^Sn]SnCl_2_)	^64^,^65^
^119^Sb	38.19 h	[^119^Sb]Sb-chloride ([^119^Sb]SbCl_3_)	^66–68^
^132^La, ^133^La, ^134^La	4.8 h, 3.912 h, 6.45 min	[^132^La]La-chloride ([^132^La]LaCl_3_), [^133^La]La-chloride ([^133^La]LaCl_3_), [^134^La]La-chloride ([^134^La]LaCl_3_)	^69–72^
^149^Tb, ^152^Tb, ^155^Tb, ^161^Tb	4.12 h, 17.5 h, 5.23 d, 6.89 d	[^149^Tb]Tb-chloride ([^149^Tb]TbCl_3_), [^152^Tb]Tb-chloride ([^152^Tb]TbCl_3_), [^155^Tb]Tb-chloride ([^155^Tb]TbCl_3_), [^161^Tb]Tb-chloride ([^161^Tb]TbCl_3_)	^73–75^
^177^Lu	6.65 d	[^177^Lu]Lu-chloride ([^177^Lu]LuCl_3_)	^76^
^153^Sm	46.28 h	[^153^Sm]Sm-chloride ([^153^Sm]SmCl_3_)	^77^
^186^Re, ^188^Re	3.72 d, 17.00 h	[^186^Re]Re-chloride ([^186^Re]ReCl_3_), [^188^Re]Re-chloride ([^188^Re]ReCl_3_)	^78–81^
^203^Pb, ^212^Pb	51.92 h, 10.6 h	[^203^Pb]Pb-chloride ([^203^Pb]PbCl_2_), [^212^Pb]Pb-chloride ([^212^Pb]PbCl_2_)	^82^
^213^Bi	45.61 min	[^213^Bi]Bi-iodide ([^213^Bi]BiI_4_^–^ and [^213^Bi]BiI_5_^2–^)	^83^,^84^
^223^Ra	11.43 d	[^223^Ra]Ra-dichloride ([^223^Ra]RaCl_2_)	^85^
^225^Ac	9.92 d	[^225^Ac]Ac-nitrate ([^225^Ac]Ac(NO_3_)_3_), [^225^Ac]Ac-chloride ([^225^Ac]AcCl_3_)	^86^,^87^
^227^Th	18.69 d	[^227^Th]Th-chloride ([^227^Th]ThCl_4_), [^227^Th]Th-nitrate ([^227^Th]Th(NO_3_)_4_)	^88^

### 2.2 Radionuclidic Purity and Precursor Species

An additional consideration, especially in radiometalated drugs, is the presence of stable contaminating elements affected by modern chelators’ wide-ranging affinities. The concept has been familiar since the early days of nuclear medicine and expressed as the “specific activity” of the radionuclide preparation in units of activity per mass of the element in question. This concept has been extended to modern radiometals, where several stable, similar mass metals (e.g., Co, Ni, and Cu) may share speciation characteristics and similar binding kinetics for the chelating ligand.^33,34^

The molar activity of a chelated radionuclide designates the ratio of the activity of the desired radionuclide to total mass of chelator required to quantitatively complex it.^89^ In these cases, the chemical preparation of the nuclide must carefully consider these stable impurities from every prior step of the process and eliminate them from the final product. Two primary methods of ascertaining successful purification exist: titrimetric reaction with the chosen chelating ligand (or complete drug molecule) to quantify the amount of metal bound in a quantitative reaction with the drug precursor and comprehensive trace metal mass spectroscopy to identify and quantify each contaminating metal constituent. The former is a relatively simple assay, analyzed by reverse thin-layer chromatography (rTLC) or high-performance liquid chromatography (HPLC), performed with a minimum of sophisticated equipment and points directly to realistic minimum injected masses from a given radionuclide preparation, but it offers few clues to the identity of an unidentified contamination. The latter is often much more decisive in troubleshooting, but it is agnostic of the relative affinities between chelators and the many elemental species that might be problematic. Both methods are discussed in more detail with examples in section 8.

## 3 Characterization of Nonradioactive Complexes

Recent advances in the field of production and purification of radiometals have provoked an increase in research efforts toward the development of new chelators. These chelators are multidentate ligands, of either acyclic or macrocyclic nature, that can complex the radiometal of interest rapidly and produce complexes that are inert toward dissociation in biological media. These ligands are prepared through synthetic organic methods, which can vary in complexity depending on the nature of the target ligand and if different functionalities are introduced for coupling to a biovector of interest or a molecular optical probe. Given the low concentration of radiometals used in radiolabeling reactions, it is essential that the ligands have a high purity and are appropriately characterized. Here we present a series of guidelines to ensure that any new chelator that may be prepared for radiolabeling studies has the necessary characterization to prove that it is adequate for subsequent labeling reactions.

An essential aspect of the synthesis of new ligands is that the synthetic procedure is correctly reported to guarantee that the experiments can be replicated without an issue. Therefore, all reagents used must be reported along with the quantities used (mass and/or volume and molar equivalents) along with a concise report of all the experimental conditions employed (duration of the reaction, temperature, purification methods, etc.). Most often, the purity necessary for radiolabeling experiments requires a final step of purification using chromatographic methods such as HPLC. The yield of the reaction should be reported as well, in terms of both percentage and mass. Afterward, the methods used to characterize the product obtained should be reported. These techniques should include at least nuclear magnetic resonance (NMR), high-resolution mass spectrometry (HRMS), and, if possible, elemental analysis (EA). Analytical HPLC traces and high-resolution mass spectra (HRMS) are also commonly used to determine the purity of a sample, although they will not necessarily show the presence of salts in the analyzed compound. In section 7.1, the methods to determine the absolute quantity of chelator are described. Other techniques such as IR can be used to determine the presence of specific functional groups, although it is not essential. The purity of synthetic intermediates does not necessarily need to be as high as for the final products; however, this should be adequately justified, and details on their characterization should be included as well.

General guidelines for each characterization technique are detailed within the Supporting Information to provide a more general description (NMR, HRMS, EA, crystallography, X-ray diffraction, and synchrotron methods), with detail on circumstances/elemental properties that may necessitate the use of specific methods for comprehensive characterization; a few methods are highlighted below with emphasis on radiochemically relevant aspects.

### 3.1 NMR Spectroscopy

NMR remains among the most powerful techniques to elucidate molecular connectivity and identity. When characterizing new chemical constructs of <1200 Da, a comprehensive spectroscopic analysis with ^1^H and ^13^C resonances listed is generally reported, although inclusion of 2D spectra is encouraged to assign the signals unequivocally. In addition to routine ^1^H and ^13^C NMR spectra, several heteroatoms (^31^P, ^15^N, ^19^F, ^29^Si) and metals (^71^Ga, ^89^Y, ^45^Sc, ^103^Rh, ^195^Pt, ^207^Pb, ^99^Tc, etc.) can be probed by multinuclear NMR methods to provide additional structural and electronic information on coordination compounds. In some cases, extremely long relaxation times and low sensitivity (^89^Y, ^103^Rh) or a very large chemical shift range (^207^Pb) of *I* = 1/2 nuclei make the recording of 1D NMR spectra very tedious, but chemical shifts can be easily accessed using 2D HMQC experiments.^90–94^ On the contrary, quadrupolar nuclei such as ^45^Sc (*I* = 7/2), ^71^Ga (*I* = 3/2) or ^139^La (*I* = 7/2) provide very broad signals due to fast relaxation.^95–98^ The line width of the NMR signals for the latter nuclei depend on the quadrupolar coupling constant and an asymmetry parameter, which are affected by the symmetry of the ligand field.^99^ As a result, highly symmetric coordination environments tend to result in easier to detect, well resolved signals, in contrast to those of asymmetric coordination spheres; the chemical shift can provide additional insight into complex speciation. Furthermore, for small complexes the line width is also proportional to the rotational correlation time, and thus experiments performed at high temperature yield sharper signals due to fast rotation.^98^ Another relevant quadrupolar NMR-compatible isotope for nuclear chemists is long-lived quadrupolar (*I* = 9/2) isotope technetium-99 (^99^Tc, *t*_1/2_ = 2.11 × 10^5^ years). ^99^Tc-NMR is a potent tool for probing oxidation state or determining solution structure of Tc complexes as well as monitoring changes in these properties that may occur over time.^100–102^ Theoretical calculations using density functional theory (DFT) have been shown to provide accurate NMR chemical shifts for different chelates and thus can be very useful for structural elucidation (section 6 below). In parallel to the NMR investigation of diamagnetic coordination compounds, the development of paramagnetic NMR in the past few decades has strengthened the ability to characterize coordination compounds containing unpaired electrons. The theory and application of paramagnetic NMR in both solution and the solid state has recently been reviewed, with examples ranging from organometallic complexes in solution to metalloproteins in solution and the solid state, pharmaceutical controlled-release formulations, and systems containing lanthanide ions.^103^ Paramagnetic coordination compounds are also important contrast agents in magnetic resonance imaging (MRI). Unless it is not accessible due to equipment limitations, the demonstration of the effect of coordination by metal ions of interest in relevant resonances must be included. This is also essential as NMR can inform on minute differences in coordination mode and provides insight into similarities and differences between coordination complexes that are intended to be used as theranostic pairs. An example of NMR characterization is provided in Figure 2, which shows ^1^H NMR spectra of chelator PYTA^4–^ recorded in D_2_O solution and the complexes with the diamagnetic ions La^3+^ and Lu^3+^. PYTA^4–^ has been proposed recently as a chelator for the inert coordination of a wide variety of radiometals (^225^Ac, ^177^Lu, ^44^Sc, and ^111^In).^104^ Coordination to La^3+^ produces noticeable changes in the spectrum, with the signals of the −CH_2_– groups of the ligand becoming diastereotopic upon coordination with the metal ion. The spectrum is consistent with the formation of a single (rigid) diastereoisomer in solution with an effective *D*_2_ symmetry and coordination number of ten. In contrast, the small Lu^3+^ ion displays an asymmetric coordination environment as indicated by the ^1^H NMR spectrum. This is related to the formation of a nine-coordinated complex in which one of the acetate pendant arms remains uncoordinated.^105,105^ NMR studies are very useful in establishing structural homology among chelates that are proposed as potential theranostic pairs. Indeed, structural differences established by nuclear magnetic resonance often are consequential for solution phase and biological nonhomology, producing different biodistribution profiles and clearance rates.

**Figure 2:**
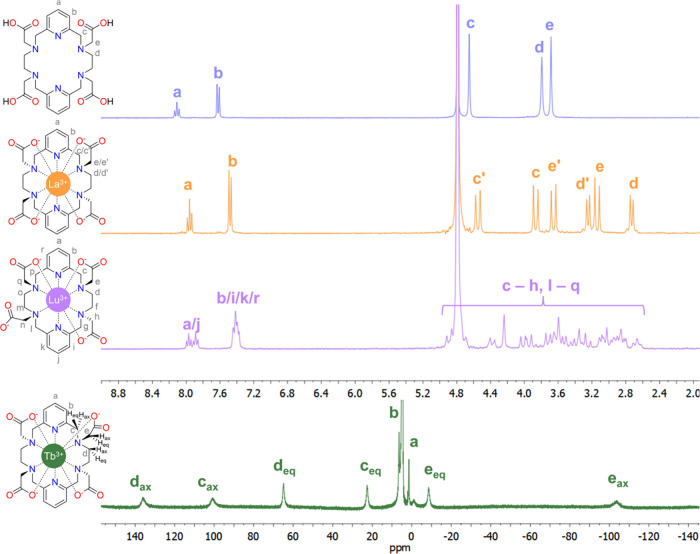
^1^H NMR spectra of chelator PYTA^4–^ and the complexes formed with diamagnetic ions La^3+^ and Lu^3+^ and paramagnetic Tb^3+^ (300 MHz, pD ∼6 for PYTA and ∼7 for the complexes, 298 K). The original spectra and spectral assignments were reported in ref 105.

The ^1^H NMR spectrum of the paramagnetic [Tb­(PYTA)]^−^ complex is also shown in Figure 2.^105^ The paramagnetism of the metal ion has two main effects in the spectrum: induction of paramagnetic shifts and enhancement of the relaxation rates of ^1^H nuclei.^106^ The paramagnetic shifts are the result of both contact and pseudocontact mechanisms. The latter depends on the spatial orientation of the observed nucleus with respect to the paramagnetic ion, encoding very useful structural information.^107^ Relaxation rates are expected to be proportional to 1/*r* ^6^ due to the action of the dipolar and Curie-spin mechanisms, where *r* is the distance between the metal ion and the nucleus and thus also offers structural information. As a result, nuclei in the vicinity of the paramagnetic ion give broader signals, which can be useful for signal attribution (i.e., axial protons give broader signals than equatorial ones). All paramagnetic Ln^3+^ ions (except Gd^3+^)^108–110^ as well as some paramagnetic transition metal ions (i.e., Co^2+^)^111^ can be studied using high-resolution NMR. Some metal ions induce very extensive line-broadening, and therefore high-resolution NMR spectra are uninformative (Mn^2+^, Cu^2+^, Gd^3+^). These complexes can however be characterized using NMR relaxometry, which studies the relaxation rates (relaxivities) of the water proton signal in solutions of the paramagnetic species over a wide range of magnetic field strengths.^112,113^

### 3.2 X-ray Crystallography

X-ray crystallography is another commonly used technique for the characterization of metal complexes, as it provides insight into the potential coordination modes of the designed chelators to the metal ion of interest. However, these crystal structures should not be interpreted as the sole structural confirmation. While they may be representative of the situation in the solid state, often one possible conformation of several that are present in solution shows preferential crystallization in the solid state. Relevant examples are the published crystal structures of [Er­(PYTA)]^−^ and [Bi­(macropa)]^+^ (Figure 3).^105,114^ Crystals of [Er­(PYTA)]^−^contain a nonacoordinated structure with one of the acetate pendant arms pointing away from the metal center. This contrasts with the recorded NMR spectrum (Figure 3a), which shows a number of signals compatible only with the decacoordinated structure with *D*_2_ symmetry. In the case of [Bi­(macropa)]^+^, the ^1^H NMR spectrum displays signals compatible with an effective *C*_2_ symmetry (Figure 3b), although the spectrum does show less defined multiplets than the [Pb­(macropa)] complex, indicating a more dynamic behavior in the case of the Bi^3+^ complex. There are 16 different possible conformations of the macrocycle with this symmetry,^115,116^ none of which are the Δ­(δλλ)­(λδδ)/Λ­(λδδ)­(δλλ) enantiomeric pair observed in the crystal structure of [Bi­(macropa)]^+^, further implying this dynamic behavior. In cases such as these, probing the solution structures using computational models (see section 6) is more adequate than identifying a single solid-state structure as the only solution-relevant structural isomer.

**Figure 3:**
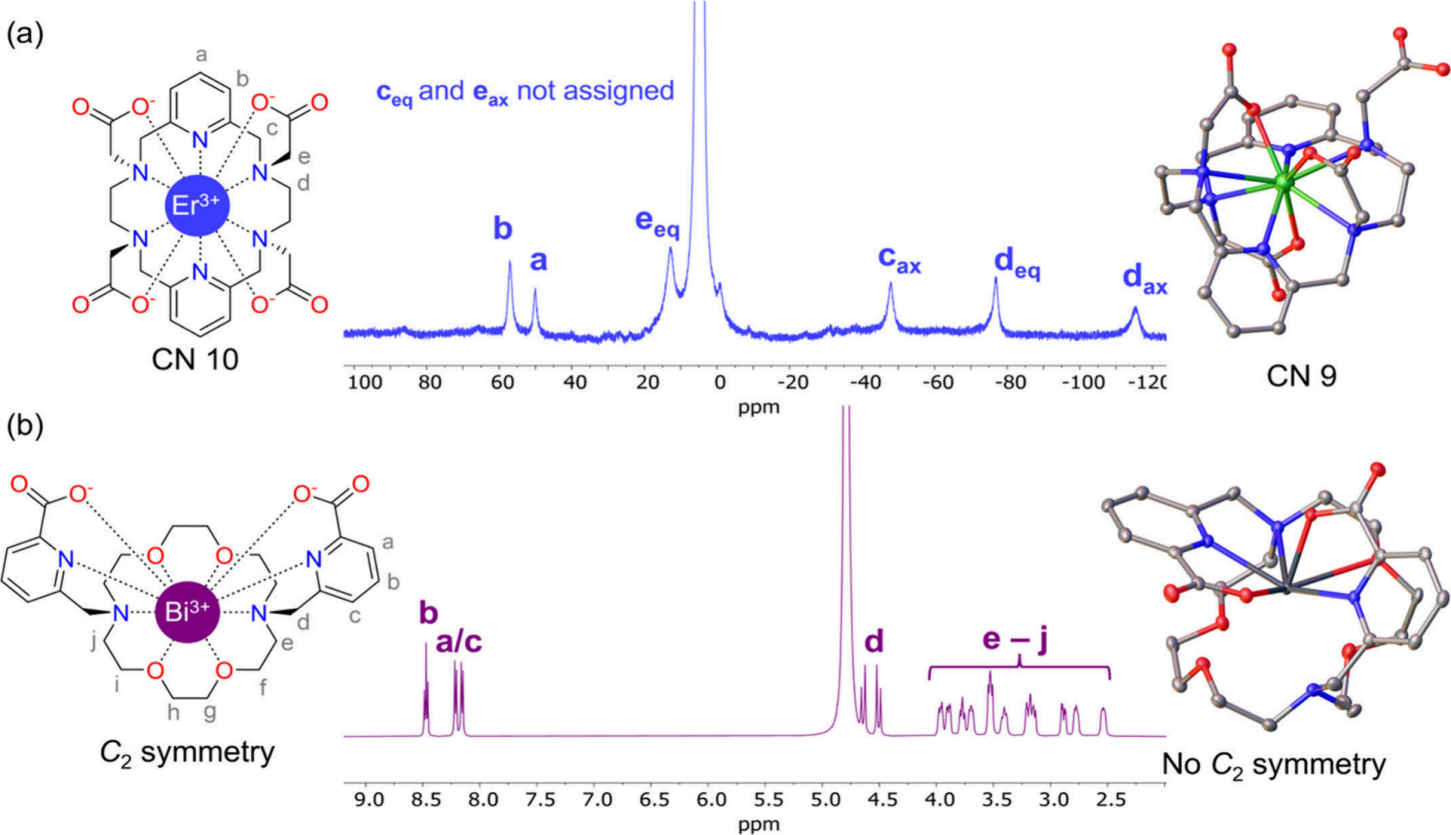
NMR spectra and crystal structures of [Er­(PYTA)]^−^ (a) and [Bi­(macropa)]^+^ (b). The original data was reported in refs 105 and 114 .

### 3.3 Absorption Spectroscopy

Absorption spectroscopy in the ultraviolet, visible, and near-infrared regions is one of the oldest and most often used characterization methods in coordination chemistry. The absorption spectra of transition metal coordination compounds in the visible or near-infrared (NIR) region are usually dictated by the energies of the d orbitals, and ligand field theory can be employed to predict the d–d transitions.^117^ In addition, charge-transfer (CT) bands in the ultraviolet–visible (UV–vis) region can offer insights into the strength and nature of metal–ligand interactions.^118^ This is especially diagnostic for first-row transition-metal ions, such as Ti^4+^, V^5+^, Cu^2+^, Co^2+/3+^ and Mn^2+/3+^, providing a means to characterize pH dependent speciation of coordination complexes using UV–vis absorption spectroscopy. While filled (d^10^) or empty d-orbitals can result in no CT or d–d absorbance bands in the visible part of the spectrum for a host of main group and transition metal ions, characteristic shifts to ligand-specific spectral features provide insight into ligand binding. This is especially useful to derive pH-dependent speciation when only limited amounts of ligand are available; section 5 provides more detailed insight and several case studies on this specific application. Furthermore, metal ions of the p block with a [Xe]­4f^14^5d^10^6s^2^ electron configuration (Pb^2+^, Bi^3+^) display characteristic absorption bands in the UV region due to 6sp ← 6s excitations that provide useful information on ligand binding.^119,120^ Electronic absorption and emission spectroscopies are also extremely useful to characterize complexes of the lanthanide ions. In general, the energy minimum of the excited state in transition metal complexes is shifted along one or several normal coordinates compared to the ground state, leading to broad d–d absorption bands due to many nonzero Franck–Condon factors.^121^ Conversely, the f–f absorption and emission bands of the lanthanide ions are narrow due to the internal character of the 4f orbitals. Some of the f–f transitions are hypersensitive to the coordination environment and have very important diagnostic value in both absorption and emission spectra.^122,123^ The lifetimes of the excited states in lanthanide complexes can also provide important structural information (i.e., the number of coordinated water molecules).^124^ Furthermore, the determination of extinction coefficients for ligands is a useful method to determine the precise ligand concentration.

### 3.4 Electron Paramagnetic Resonance Spectroscopy

Compared to NMR spectroscopy, electron paramagnetic resonance (EPR) spectroscopy is a less routine analytical tool for most synthetic laboratories but is critical to understand the electronic structure of paramagnetic metal centers in diverse ligand environments.^125^ EPR, also known as electron spin resonance spectroscopy (ESR), provides detailed information about the electronic structure of metal centers with unpaired electrons and interactions with neighboring nuclear or electron spins.^126^ EPR signals are characteristic of the metal, oxidation, and spin state, and can be used to identify the presence of a particular species, and do so quantitatively.^127^ Finally, advances in molecular orbital calculations, primarily using density functional theory, have dramatically enhanced the interpretation of experimental data (see section 6). Most commonly, EPR is employed to characterize solution structures of Cu^2+^complexes (Figure 4)^128^ to affirm or contrast structural insight gained from computational and crystallographic data.^129–135^

**Figure 4:**
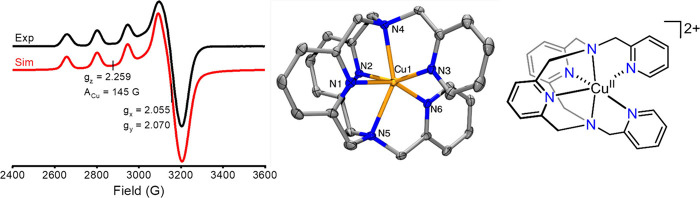
EPR spectrum (black) and simulation (red) of the [(^Pic^N4)­Cu^II^]­(OTf)_2_ complex, obtained in a 1:3 MeCN:PrCN solution mixture; the spectrum was collected as a frozen glass at 77 K, and the simulated spectrum was obtained using the *g* and *A*_Cu_ values shown above. The crystal structure of [(^Pic^N4)­Cu^II^]^2+^ is shown on the right. The original data was reported in ref 128.

### 3.5 Cyclic Voltammetry

Electrochemical techniques can be employed for a wide range of applications in coordination chemistry.^136^ A number of radiopharmaceutically relevant elements display electrochemical noninnocence in aqueous media under biologically relevant conditions, such as Cu, Co, Mn, Tc, Re, Pt, or Th. The characterization of the electrochemical behavior of coordination complexes of these elements, identification of relevant redox events, and evaluation of the chemical stability of species at various oxidation states provide a comprehensive picture of their solution behavior. The most commonly employed techniques used by coordination chemists undertaking electrochemical experiments are 1) voltammetry under transient (e.g., cyclic voltammetry)^137^ or steady-state (e.g., rotated disk or microelectrode) conditions, which requires the interpretation of current–potential–time (*I*–*E*–*t*) curves; 2) spectroelectrochemical measurements in which a spectroscopic or other method of measurement (e.g., mass spectrometry) is used in conjunction with electrochemistry to characterize intermediates or products of electrode processes; and 3) bulk electrolysis for the purpose of electrosynthesis or for a coulometric determination of the number of electrons associated with a redox reaction or a half-cell reaction.^138^ Importantly, it is essential that such electrochemical methods are performed under conditions that are similar to those under which the coordination compounds are used for biological applications (i.e., under aqueous conditions at the appropriate pH). In addition, the electrode potentials need to be measured correctly, and the appropriate reference potentials must be used when converting between various electrode potential scales, to allow for a direct comparison among results obtained by various research groups.^139^ Most commonly employed reference potentials are Normal Hydrogen Electrode (NHE), as well as Ag/AgCl (+0.197 V vs NHE when filled with saturated KCl; +0.210 V when filled with 3 M KCl) and the saturated calomel electrode (SCE, +0.241 V vs NHE).^136,140,141^

For contemporary radiopharmaceutical applications, the most extensively studied redox events are those of Cu^2+/+^, Mn^3+/2+^ and Co^3+/2+^. Representative cyclic voltammetry experiments reported for Cu^2+^chelates^142^ and Mn^2+^ complexes^143^ are shown in Figure 5,^143^ recorded using a Ag/AgCl reference electrode and a glassy carbon working electrode. The two complexes provide quasi-reversible waves with separations of the anodic and cathodic waves slightly above the 57 mV predicted for a one-electron reversible process. The reduction half-wave potential obtained for [Cu­(CBTE1A)]^+^ (−0.92 V vs Ag/AgCl filled with 3 M KCl, which corresponds to −0.61 V vs NHE) is clearly out of the window of common reducing agents present *in vivo*. This is expected to preclude complex dissociation in the presence of bioreducing agents, such as ascorbate. The half-wave potential observed for [Mn­(CHXPYAN)]^2+^ is +0.57 V, which corresponds to +0.78 V with respect to NHE. This indicates that the Mn^2+^ complex is rather resistant to oxidation, as the potential for the oxygen reduction to water is *E*O_2_/H_2_O = +0.82 V vs NHE at pH 7. For the Cu^2+/+^, Mn^3+/2+^ and Co^3+/2+^ pairs, both oxidation states can be stabilized under biological conditions and compatible with radiopharmaceutical applications. This may be achieved in two ways: a) by stabilizing one oxidation state over the other to prevent any redox events *in vivo* or b) by employing chelates that can stabilize both oxidation states in a stable manner. In terms of electrochemical behavior, this would mean that in scenario a) the redox potential lies outside the redox potential for glutathione and ascorbate, or even beyond the oxygen reduction to water and is generally reversible or quasi-reversible, with closely spaced reduction and oxidation waves. The majority of Cu^2+^ chelates such as NOTA^3–^, CBTE1A^–^ and others belong to this class, as well as several Mn^2+^ chelates.^144,145^ For scenario b) several Co^2+/3+^ and Ni^2+/3+^ complexes have been characterized as stable in both forms *in vivo*.^146,147^ Their cyclic voltammograms appear irreversible, or the reduction and oxidation waves are separated by a large voltage difference; yet both oxidation states are accessible. Such behavior is commonly associated with a structural change of the chelate or a change in coordination geometry and/or coordination number of the corresponding metal complex, yet the redox event is actually reversible, without dechelation or other complex degradation events.

**Figure 5:**
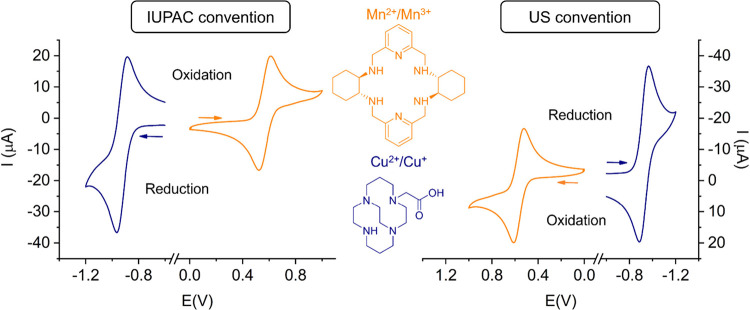
Cyclic voltammograms recorded from ∼2 mM aqueous solutions for representative Cu^2+^ and Mn^2+^ complexes in 0.15 M NaCl, which show quasi-reversible waves due to the Cu^2+^/Cu^+^ (250 mV/s) and Mn^3+^/Mn^2+^ (500 mV/s) pairs. The left panel follows the IUPAC convention and the right panel the US convention. Potentials are reported versus Ag/AgCl. The original data was reported in refs 142 and 143 .

## 4 Characterizing the Chemistry of Nuclides without Stable Isotopes

Generally, characterization data of complexes of the observationally stable or, informally, “cold” coordination complexes on the macroscopic scale provide appropriate prediction of trace chemical behavior. However, for some radioisotopes of interest in nuclear medicine, no observationally stable isotopes exist (Table 2). In these cases, congeners can be used to obtain structural information to guide and evaluate the potential of the ligands of interest toward the radiometal. Alternatively, computational studies that do not require access to the radioactive isotope can be performed to further aid in chemical discovery and characterization.^102,148–152^ The most commonly investigated, short-lived radionuclides with no stable isotopes are technetium (^99m^Tc, *t*_1/2_ = 6.0 h), actinium (^225^Ac, *t*_1/2_ = 9.9 d), radium (^223^Ra, *t*_1/2_ = 11.4 d), thorium (^227^Th, *t*_1/2_ = 18.7 d), and astatine (^221^At, *t*_1/2_ = 7.2 h). In the case of these first four isotopes, it is possible to find other elements with similar chemical properties or long-lived isotopes of the same element. With respect to astatine, this is complicated by the lack of comprehensive radiochemical characterization data, and poorly delineated chemical reactivity with similarity to both heavier halides and main group metals.^153,154^

**Table 2:** Summary of Unstable Elements, Long-Lived Isotopes, and Congeners

**Nuclide**	**Half-life (*t*_1/2_)**	**Long-lived isotopes and their half-lives**	**Ionic radius (Å) of most common oxidation states** ^157^	**Congeners and relevant ionic radii (Å)**	**Selected references**
^99m^Tc	6.01 h	^99^Tc, *t*_1/2_ = 2.11 × 10^5^ years	Tc^5+^ 0.60 (CN 6)	Re^5+^ 0.58 (CN 6)	^156^,^159^,^162^,^174–176^
^223^Ra	11.43 d	^226^Ra, *t*_1/2_ = 1600 years	Ra^2+^ 1.48 (CN 8)	Ba^2+^ 1.42 (CN 8)	^167^,^168^,^177–180^
^225^Ac	9.92 d	^227^Ac, *t*_1/2_ = 21.77 years	Ac^3+^ 1.12 (CN 6)	La^3+^ 1.032 (CN 6)	^165^,^181–184^
^227^Th	18.69 d	^232^Th, *t*_1/2_ = 1.40 × 10^10^ years	Th^4+^ 0.94 (CN 6)	Ce^4+^ 0.87 (CN 6)	^185–188^

Technetium is the lightest element with no stable isotopes, belonging to period 5 and group 7 of the periodic table. A wide range of oxidation states (from +1 to +7) have been characterized, with most common, water-stable oxidation states being +1, +3 and +5. Technetium possesses a long-lived isotope, technetium-99 (^99^Tc, *t*_1/2_ = 2.11 × 10^5^ years), which is accessible as a nuclear fission product.^155^ ^99^Tc has been used to obtain crystal structures and characterize other macroscopic properties of technetium species.^156^ However, access to this isotope is often prevented by regulatory hurdles and increasingly scarce resources, which is why most studies employ rhenium as a congener. Re belongs to the same group as Tc and presents a slightly smaller ionic radius than technetium (0.58 Å vs 0.60 Å for CN 6 and oxidation state +5).^157^ The chemical properties of Re are comparable to those of Tc, which makes it an ideal stable congener that is more accessible to research groups developing chelators for Tc-based radiopharmaceuticals. Thus, ^99m^Tc complexes are often characterized by comparing the HPLC trace of the technetium complex with that of the fully characterized rhenium analogue.^158^ However, the chemistry of technetium and that of rhenium are not identical, and the behavior of ^99m^Tc radiopharmaceuticals may not be adequately predicted by rhenium chemistry.^159^ Specifically, these elements show significant differences in their redox chemistry arising from a higher stability of the +7 oxidation state in Re compared to Tc.^160^ Furthermore, differences in reactivity toward isomerization and ligand substitution reactions have also been described.^161,162^ A remarkable case of different behavior are the attempts to extend the bifunctional HYNIC strategy developed for technetium to rhenium-188 (^188^Re, *t*_1/2_ = 17.0 h) radiopharmaceuticals, where results were less favorable, with authors theorizing that this effect is due to different redox and kinetic behavior.^163,164^ For these reasons, the use of Re as an analogue of Tc must be taken with some caution.

Similarly, longer-lived isotopes of actinium and radium can be used in properly equipped laboratories, such as actinium-227 (^227^Ac, *t*_1/2_ = 21.77 years) and radium-226 (^226^Ra, *t*_1/2_ = 1600 years). It should be mentioned that ^227^Ac is mainly of interest for producing α emitters ^223^Ra and ^227^Th and that production can be carried out through different pathways such as irradiation of ^226^Ra or extraction from protactinium-231 (^231^Pa, *t*_1/2_ = 3.28 × 10^4^ years).^165,166^ At present, only small research-scale amounts (mCi) are available from specialized facilities (e.g., Oak Ridge National Laboratory), though one research group has used legacy protactinium-231 samples to extract quantities sufficient for crystallization.^165^ Recent publications describe the first Ac^3+^ and Ra^2+^ coordination complex crystal structures, using ^227^Ac and ^226^Ra, demonstrating that there is a large difference in coordination modes when compared with stable congener complexes.^165,167,168^ The most closely chemically homologous congeners for these elements are lanthanum and barium, respectively. The ionic radii of these elements differ more than in the case of Tc and Re, with the radioactive elements possessing larger ionic radii (1.12 Å for Ac^3+^ and 1.032 Å for La^3+^, CN 6; 1.48 Å for Ra^2+^ and 1.42 for Ba^2+^, CN 8).^157^ Also, for Ac^3+^ vs La^3+^ the increased covalency of early actinides, in contrast with the lanthanide series, needs to be considered.^169–173^

Finally, thorium presents a slightly more difficult case. While a long-lived congener, thorium-232 (^232^Th, *t*_1/2_ = 1.40 × 10^10^ years), is available and can be used to obtain thermodynamic stability data or X-ray crystal structures,^185,189^ finding an adequate, stable congener to act as a model of this element is more challenging. The most common oxidation state of thorium is +4, and while the lanthanide cerium could be considered a suitable stable congener, it favors the oxidation state +3. In contrast, as Ce^4+^ can be stabilized, differences in coordinative and redox behavior complicate viable comparisons.^190,191^ Additionally, their ionic radii differ to a similar extent to Ac^3+^/La^3+^or Ra^2+^/Ba^2+^ (0.94 Å for Th^4+^ and 0.87 Å for Ce^4+^, CN 6).^157^ Nevertheless, a Ce^4+^ radioisotope, cerium-134 (^134^Ce, *t*_1/2_ = 75.84 h) has been proposed as an imaging surrogate for ^227^Th,^192^ along with the Zr^4+^ isotope zirconium-89 (^89^Zr, *t*_1/2_ = 78.41 h).^193–195^

## 5 Thermodynamic Stability Measurements, Complex Formation and Decomplexation Kinetics

In the study of metal complexes, the determination of thermodynamic constants is crucial for understanding the stability and binding properties of these complexes in aqueous media. These constants include protonation constants of the ligands and complexation constants, which may involve a variety of species, such as hydroxido complexes, species with different numbers of ligands, and oligonuclear metal complexes. Accurate measurements of these constants are essential for characterizing relevant metal–ligand interactions, predicting complex stability, and aiding in ligand design. The variation of the complexation constants for a single ligand for different metal ions indicates selectivity and, in some cases, specificity. For metal ions with redox activity, redox state dependent speciation should be considered,^196^ but data acquisition may be limited by parameters required to conduct reliable and reproducible measurements.

### 5.1 Methods for Measuring Thermodynamic Constants

Several experimental techniques are commonly used to measure thermodynamic constants, each offering unique advantages depending on the system under study. These include potentiometry, NMR, and UV–vis absorption spectroscopy. Other, less frequently used techniques comprise EPR spectroscopy,^197^ electrochemical techniques such as cyclic voltammetry and differential pulse polarography,^198–200^ and isothermal titration calorimetry (ITC).^201,202^ Each method can provide valuable insight into the metal–ligand binding equilibrium, though they differ in the specific type of data they produce and the conditions required for measurement. For instance, electrochemical techniques are limited to systems displaying electrochemical response, while ITC shows limitations for the determination of high stability constants (> ∼10^8^). Additionally, commonly used potentiometry presents the limitation that significant complex dissociation must take place in the pH range of 2–12. Measurements are generally conducted at 25 °C, even if the constructs are to be applied at 37 °C, as the vast majority of data reported in the literature were measured at 25 °C. One might also consider different concentration regimes, as radiopharmaceutical applications often require measurements at nanomolar to micromolar concentrations, compared to the millimolar range commonly used in traditional thermodynamic studies. Similarly, the ligand-to-metal stoichiometry may differ between macroscopic and radioactive tracer chemistry, as radiolabeling is generally performed using a large excess of the chelator, highlighting the importance of careful consideration of relevant experimental conditions when identifying various species in solution through standard coordination studies.

The measurement of thermodynamic constants for metal complexes requires a combination of experimental techniques and careful data analysis. In many cases, a combination of these methodologies is required: for instance, spectrophotometry and NMR to explore the extreme pH (outside pH range ∼2–12) values that are not easily accessible with potentiometry. By utilizing potentiometry, NMR, UV–vis, and EPR spectroscopy and employing appropriate software for each technique, one can gain a comprehensive understanding of the complexation behavior of metal ions in solution. These studies offer helpful insights into chelator design, complex stability, and the fundamental principles of metal–ligand interactions. While potentiometry provides critical quantitative data on binding constants, it lacks the structural information that spectroscopic techniques such as NMR, EPR, or UV–vis spectroscopy can offer. Combining potentiometry with at least one other spectroscopic method is essential to obtain a complete understanding of the system.

Potentiometry itself is one of the most widely used methods for determining protonation and complexation constants ([C] > 10^–3^ M). It is especially valuable for systems involving multiple equilibria (e.g., protonation, deprotonation, and metal binding). Potentiometric pH titrations in the presence of metal ions are often performed from low to high pH by addition of a solution of a standardized base to prevent formation of metal-hydroxide species. Excessive carbonation can lead to inaccurate results as the concentration of the basic titrant is altered. Software packages such as Hyperquad are commonly employed for the analysis of potentiometric titration data.^203,204^ This software allows for the fitting of experimental data to complex equilibrium models, providing a robust and reproducible determination of thermodynamic constants. Experiments should be performed at least in triplicate, which, considering the sensitivity of the technique and technical issues (typically ∼2 mM solutions of the chelator are titrated and volumes >5 mL are required) and the fact that the chelator must be titrated both in the presence and in the absence of the metal ion, can require a minimum of 4–10 mg of chelator for each metal ion investigated. For chelators synthesized using 6–10 synthetic steps or those with limited solubility in the mM concentration range, this represents a challenge, necessitating the use of other, more sensitive techniques compatible with the use of lower concentrations in solution, such as UV–vis spectrophotometric titrations (see below). A recent review discusses in detail the methodology and potential biases in the determination of equilibrium constants using potentiometry.^205^

As mentioned previously, alternative techniques such as NMR and UV–vis spectroscopy can be employed as surrogate methods under conditions in which the pH of dissociation is not compatible with potentiometry. NMR is a powerful tool for studying the solution structures of the protonated species and thermodynamics of metal complexes of diamagnetic metal ions ([C] ∼ 10^–3^ M). By monitoring the chemical shifts of nuclei in the ligand, NMR can provide detailed information about the protonation and coordination environment and the stepwise binding of metal ions. For paramagnetic metal ions, which often cause broadening of NMR signals, conventional high-resolution studies may be limited to some metal ions with favorable relaxation properties. Otherwise, specialized techniques such as relaxometry may be used to extract relevant information, typically by measuring the relaxivity of a solution of a paramagnetic complex at variable pH.^206^ For NMR speciation studies, often deuterated (D_2_O, NaOD, DCl) compounds are used; accordingly, the pD values must be reported and not the direct pH reading provided by the glass electrode (pH*), which have to be converted following data analysis (pD = pH* + 0.40).^207–209^ HypNMR is a widely used software for analyzing NMR titration data.^210,211^ It can model multiple equilibria in solution using a defined number of characteristic proton signals, making it particularly useful for systems where several species coexist, such as complexes with different numbers of ligands or metal ions.

UV–vis spectroscopy is another important technique for studying metal–ligand interactions, particularly for complexes that exhibit distinct absorbance changes as a function of metal binding or protonation ([C] depends of the extinction coefficient of the studied molecules, but usually 10^–5^ < [C] < 10^–3^ M, with ∼10^–3^ M being required when using Laporte forbidden d–d transitions). By recording spectra at different pH values or metal-to-ligand ratios (depending on the denticity of the ligand), one can track the formation of different species in solution. This method is especially useful when the complex formation is accompanied by a color change or a significant shift in the maximum absorbance (e.g., changes of at least 0.2 absorbance units); in other cases, alternative techniques should be used. UV–vis speciation can determine protonation constants and complex formation constants but requires distinct absorbance profiles for different solution species to produce reliable fits. Furthermore, this method may be useful when the complex formed is paramagnetic and therefore not suitable for NMR characterization of speciation or when the ligand and metal complexes exhibit limited solubility. Software packages such as ReactLab,^212^ Hyperquad or HypSpec are typically used for analyzing UV–vis data.^203,204^ It allows for the fitting of absorbance spectra to models that include multiple species, enabling the determination of formation constants for both metal complexes and protonated species.

A typical set of spectrophotometric titrations used to determine equilibrium constants is shown in Figure 6. The absorption band of the pyridyl units of PYTA^4–^ varies considerably depending on the protonation state, which allows for the determination of the ligand protonation constants and the assignment of the different protonation sites. Up to five protonation constants were obtained from simultaneous fits of spectrophotometric and potentiometric data, as shown in the speciation diagram presented in Figure 6c. Once the protonation constants were obtained, potentiometric titrations were used to obtain the protonation constants of the complex. However, pH potentiometry does not allow the determination of the stability constant of the complex, as complex dissociation occurs below pH ∼ 2 (see speciation diagram). Nevertheless, spectrophotometric titrations can be used at higher proton concentrations. For example, this allows the determination of the stability constant of log *K*_PbL_ = 24.63(3). Importantly, an incorrect stability constant value was reported previously from the fit of potentiometric data only (log *K*_PbL_ = 17.7),^213^ highlighting the importance of using a backup method to check the equilibrium model employed for potentiometric data analysis.

**Figure 6:**
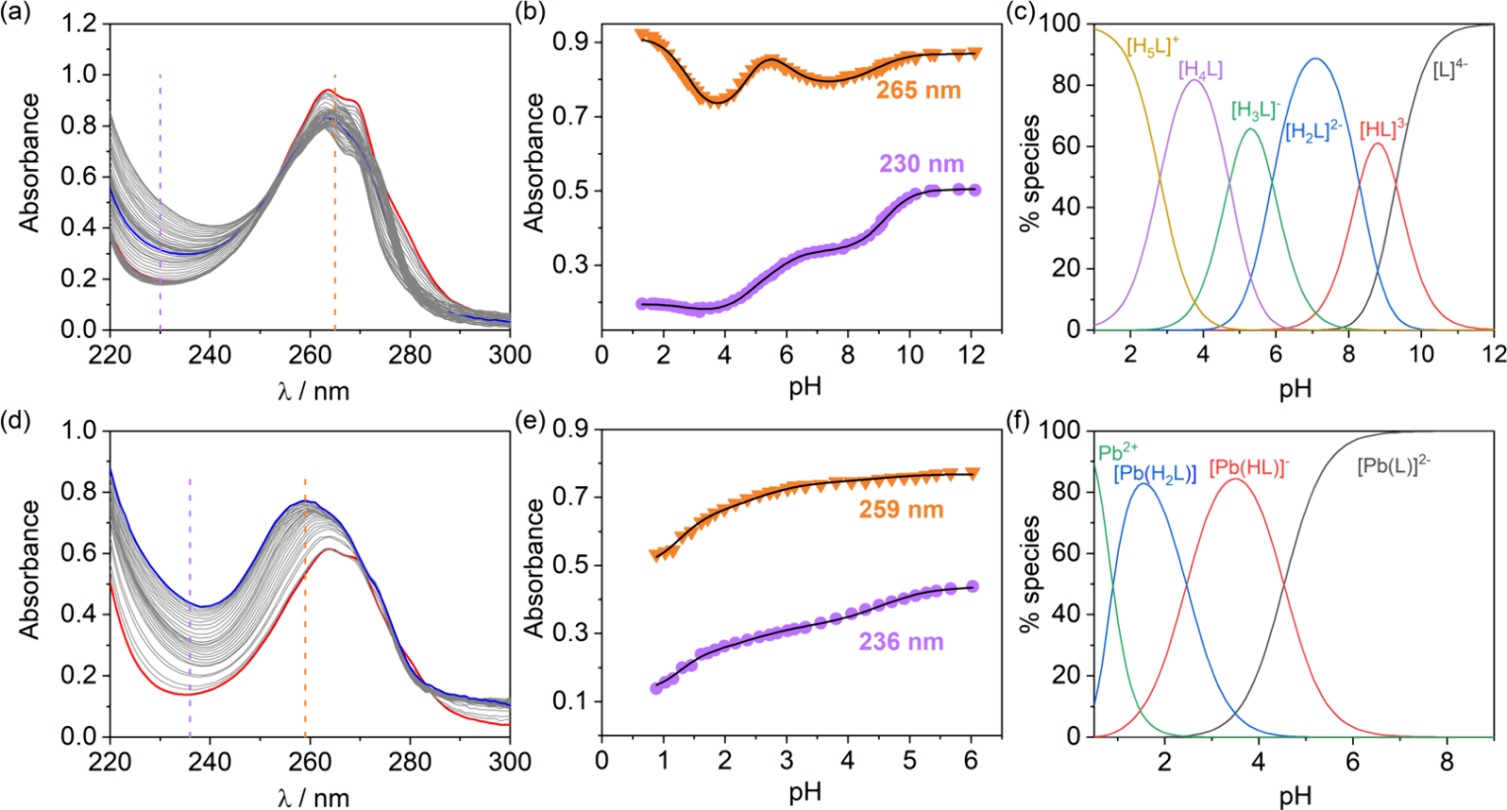
Top panel: (a) Spectrophotometric titration of PYTA^4–^ (10^–4^ M, 25 °C, 0.15 M Na­(ClO_4_), which shows significant changes in the absorption band of the pyridyl unit with pH. (b) Spectral changes at selected wavelengths showing the fits of the data to obtain protonation constants. (c) Speciation diagram calculated with the protonation constants for [L] = 10^–3^ M. Bottom panel: (d) Spectrophotometric titration of PYTA^4–^ in the presence of 1 equiv of Pb^2+^. (e) Absorbance changes at selected wavelengths. (f) Speciation diagram obtained with the stability and protonation constants for [L] = [Pb^2+^] = 10^–3^ M. The original data was reported in ref 213.

Comparison of thermodynamic binding constants of the same ligand with different metal ions can inform the relative binding strength and selectivity. However, it is not possible to compare the performance of two different ligands with the same cation, because the differing basicity of the ligands both with respect to p*K*_a_ and number of protons results in noncomparable log *K*_HLM_ values. Of course, conditional stability constants can be obtained at any pH once the ligand protonation constants and stability constants are known. Very often, pM values, defined as pM = −log­[M_free_], are calculated and used for comparing the stabilities of different complexes with a given metal. The numerical value of free metal ion concentration in solution under given conditions enables quantitative comparison of different ligands. Most commonly, pM values are reported at pH = 7.4, with [M]_tot_ = 10^–6^ M, [L]_tot_ = 10^–5^ M, which are the conditions originally suggested by Raymond and co-workers.^214^ However, pM values calculated using different conditions are sometimes reported (i.e., for Mn^2+^ complexes often for [M]_tot_ = [L]_tot_ = 10^–5^ M),^215^ and care should be taken not to compare values obtained using different concentrations or pH values.

Table 3 shows thermodynamic stability data for a series of Ga^3+^ complexes with representative chelators. The values of the stability constants vary over 17 orders of magnitude for this series of complexes from log *K*_GaL_ = 21.33 for 1,4,7,10-tetraazacyclododecane-1,4,7,10-tetraacetic acid (DOTA^4–^) to 38.51 for *N*,*N*′-bis­(2-hydroxybenzyl)­ethylenediamine-*N*,*N*′-diacetic acid (HBED^4–^). However, the pGa values do not show such large differences due to the different ligand basicities, as estimated by the Σlog *K*_HiL_ values. This effect is particularly evident for HBED^4–^ and 1,4,7-triazacyclononane-1,4,7-triacetic acid (NOTA^3–^), which show *K*_GaL_ values differing by nearly 8 orders of magnitude, but very similar pGa values, indicating similar concentrations of free Ga^3+^ at the conditions used to calculate pGa. Of note, the pGa values shown in Table 3 were obtained using all equilibrium data reported in the original references, including complex protonation constants, formation of hydroxido species, and the hydrolysis constants reported for Ga^3+^. However, pGa values presented in Table 3 and in the literature do not consider the amount of uncomplexed metal present in the form of [Ga­(OH)_4_]^−^ and other hydroxo species,^216,221^ and thus do not reflect the actual amount of Ga^3+^ that is not chelated by the ligand. This issue is relevant only for metal ions with a high tendency to form hydroxo-complexes (i.e., Bi^3+^ and even Pb^2+^).^222^

**Table 3:**
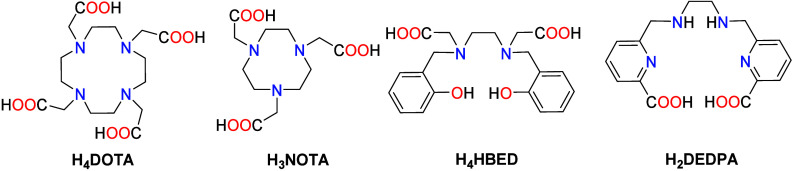
Common Chelators Used for Ga^3+^ Complexation, Their Protonation Constants and Overall Basicity, Stability Constants of the Complexes, and pGa Values

**Ligand**	**log** ***K***_**HiL**_	**Σlog** ***K***_**HiL**_	**log** ***K***_**GaL**_	**pGa** ^a^	**Selected references**
DEDPA	9.00; 6.31; 3.04; 2.59	20.94	22.9	22.2	^216^,^217^
HBED	12.64; 11.03; 8.34; 4.40; 2.24	36.41	38.51	29.6	^218^
NOTA	10.773; 6.032; 3.163; 1.955	21.92	30.98	28.5	^219^
DOTA	11.14; 9.69; 4.84; 3.95	29.62	21.33	20.0	^220^

a pGa is defined at pH 7.4 for [Ga^3+^]_tot_ = 10^–6^ M, [L]_tot_ = 10^–5^ M.

Some chelators may present stability or solubility problems over the wide pH range required for the determination of protonation constants. Under these circumstances, conditional association constants are often reported, most commonly determined using spectrophotometric (or fluorometric) titrations or ITC.^223^ These experiments are conducted in buffered solutions to maintain the pH constant, often at pH 4. Of note, association constants determined in different buffers may not be comparable due to their different binding ability. Some authors analyze binding affinity using dissociation constant *K*_d_, which is the inverse of the association constant.

Complexation kinetics can pose significant limitations on the measurement of thermodynamics. Indeed, in plenty of cases, the complexation process is relatively slow in acidic medium or all along the pH scale. This means that complexation may not occur in the time scale of the experiments (especially by potentiometric titrations where the electrodes cannot stay calibrated for a long time, specifically in acidic/basic media). In these cases, a batch method is indicated: several batches of an appropriate ligand/metal ratio are prepared at different pH values (generally at least 20–25 batches between pH 2 and 12), and the potentiometric measurements are performed after equilibrium is achieved. This process may require weeks or even months. While systems that present this behavior are likely not appropriate for radiopharmaceutical applications, they may be very valuable for aiding chelator design and for the rationalization of radiolabeling studies.

On the other hand, some complexes of chelating ligands form rapidly and almost quantitatively at the onset of the experiment under acidic conditions, and thus measurement of the complex formation constants may require competitive studies. In competition experiments, the effectiveness of different ligands to coordinate the same metal ion in solution is investigated. This process involves several crucial steps: 1) Finding the right ligand to compete with the target ligand; the choice of competing ligand depends on its affinity and ability to compete effectively under appropriate experimental conditions. Often acyclic ligands such as ethylenediaminetetraacetic acid (EDTA) or ethylene glycol-bis­(2-aminoethoxy)-tetraacetatic acid (EGTA) are employed as competing ligands, as they display fast complexation kinetics. 2) Determining the optimal stoichiometry of the competing ligand: to ensure accurate competition, the optimal ratio of the competing ligand must be determined. This often requires computational modeling, and software tools like HySS are useful for this purpose as they help simulate the required dosage to achieve effective competition.^224^ 3) Batch studies to account for slow transchelation kinetics: the kinetics of the transchelation process is frequently too slow for a single, serial titration approach. Careful consideration of these steps ensures that equilibrium is reached and accurate competition data are gathered.

Finally, the determination of thermodynamic stability constants can also be applied to radioactive isotopes, for example, to investigate the stabilities of complexes with metal ions that lack stable radioisotopes, such as those discussed in section 4. The sensitivity of spectrophotometric and spectrofluorometric methods are particularly useful for these situations. For instance, the presence of chromophores with high extinction coefficients allowed the determination of stability constants of [^232^Th]­Th^4+^ complexes with HOPO derivatives using concentrations in the ∼10^–5^ M range.^193,350^ In another example, the stability constant of the Ac^3+^-HOPO complex was determined using spectrofluorimetric titrations with Eu^3+^ as a competitor. The Eu^3+^-HOPO complex is highly luminescent and displays a stability that is in the right range for competition experiments, allowing the determination of stability constants using only 1.70 μg of the long-lived [^227^Ac]­Ac^3+^ ion.^165^ However, alternative (radioanalytical) methods are necessary when spectrophotometric or spectrofluorometric methods are not suitable, due to the small quantities of radioisotopes available. Thiele et al. used the competitive cation exchange method to determine the stability constants of [^223^Ra]­Ra^2+^ complexes with macropa and other macrocyclic ligands. These experiments measure the distribution coefficient (*D* value) defined as the ratio of activity adsorbed to the resin versus that of the aqueous phase at varying concentrations of the chelator (Figure 7).^150^ This allows the determination of the apparent cumulative stability constant (β_app_) by linear regression using eq 1, where *D*_0_ represents the distribution coefficient in the absence of a chelator. Experiments at different pH values allow the determination of the pH-independent stability constant (log *K*_ML_).

$$\frac{D_{0}}{D}-1=\beta_{app}\left[chelator\right]$$ 1

A similar linear expression can be used to determine equilibrium constants using solvent extraction experiments, as in the case of the determination of the stability constant of the [^99^Tc]­Tc^4+^-DTPA complex and An^3+^ complexes of macropa.^351,352^

**Figure 7:**
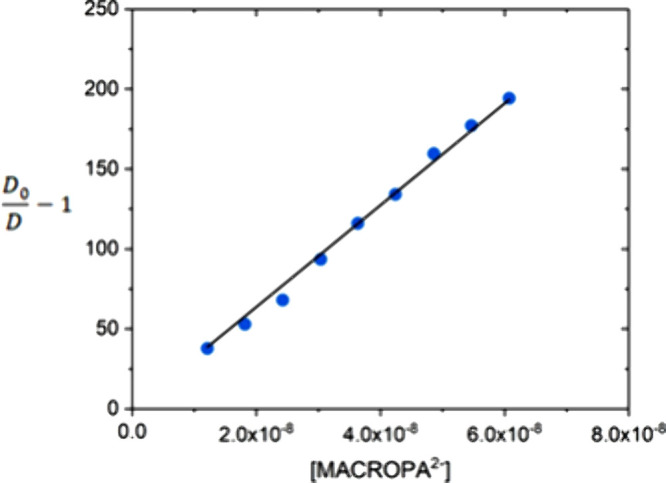
Determination of β_app_ for the [^223^Ra]­Ra^2+^ complex of macropa carried out using the competitive cation exchange method at pH 5.65. The slope of the linear fit affords log β_app_ = 9.51. The original data was reported in ref 150.

### 5.2 Complex Formation and Decomplexation Kinetics

Two important requirements for ligands for metal-based radiopharmaceuticals are fast complex formation and high inertness (slow metal ion and ligand exchange, i.e., slow decomplexation). To be able to improve the efficiency of complex formation and prevent complex lability, the overall formation and decay rates are not sufficient to accurately understand the pathways, and a thorough study of the complexation and decomplexation mechanisms requires a detailed analysis of the reaction kinetics, combined with various other experimentally and/or computationally determined features. These may include 1) the analysis of the product distributions (e.g., various isomers), 2) the trapping and spectroscopic or structural characterization of intermediates (e.g., there are examples, where it is difficult to transform in a fast pre-equilibrium formed complex species into the stable final complexes, and often these are named “out of cage isomer” without detailed analysis of their structures, which might allow to prevent them via ligand modification), and 3) the structural analysis of the final complex. The combination of experimental and computational data may help to fully understand the mechanistic pathways. An example of the thorough kinetic and mechanistic analysis of complex formation is that of the Cu^2+^ complexes of cyclam-type tetraazamacrocycles (cyclam = 1,4,8,11-tetraazacyclotetradecane).^225^ There are various examples, where relatively labile “out of cage” intermediates, that may be difficult to transform to the fully encapsulated inert complex, have been observed.^226–229^ Techniques for the structural analysis of isomers of the complexes, including intermediates, e.g., the out of cage forms, include various spectroscopies, possibly in combination with computational analyses, and X-ray crystallography (see sections 3.2 and 6).

The complex’s kinetic inertness, of course, is an important feature in the full characterization of the final complex. It is helpful when data of the metal–ligand system under consideration (specifically kinetic and thermodynamic data) are produced under identical conditions with respect to solvent, temperature, ionic strength, and medium (solvent, inert salt). As radiochemical and biological experiments are not conducted under these conditions, correlation or extrapolation to biological/tracer level kinetics may be limited, but identical conditions in the kinetic and thermodynamic studies certainly are helpful for qualitative extrapolations.

The decomplexation kinetics may involve acid-, base-, metal-, redox-, and ligand-dependent terms. H^+^ and OH^–^ induced reactions are relevant under physiological conditions because at pH 7, the H^+^ and OH^–^ concentrations (10^–7^ M) are about 3 orders of magnitude higher than the metal complex concentration (≈10^–10^ M), corresponding to a complex to H^+^ (or OH^–^) ratio at a 10^–3^ M complex concentration at approximately pH 0 or pH 14. Therefore, pH dependent kinetics are relevant and show whether obvious decomplexation pathways (microscopic reversibility, i.e., protonation of the ligand (M^*n*+^/H^+^ competition), and hydrolysis, i.e., formation of stable metal-hydroxido species) are prevented by the ligand design.^229^ Some metal ions (in particular Cu^2+^ and Zn^2+^) and anions (e.g., bicarbonate, citrate) are present in body fluids at relatively high concentrations and are known to trigger complex dissociation.^230^ Additionally, complexes of redox active metal ions (e.g., Cu^2+^) can be reduced *in vivo*, which may provide an additional pathway for complex dissociation. For instance, very inert Cu^2+^ complexes with respect to acid-initiated dissociation may dissociate relatively fast in the presence of reducing agents such as ascorbate, if the Cu^+^ oxidation state is accessible.^231^ Some metal ions also form strong complexes with proteins, such as Ga^3+^ with human serum transferrin, which may offer additional dissociation mechanisms *in vivo*.^232^ In terms of metal ion decomplexation, it is therefore important to consider the entire landscape of dissociation pathways when assessing the kinetic inertness of complexes for radiopharmaceutical applications. Often kinetic inertness is judged by following dissociation in strongly acidic conditions ([H^+^] = 1 M or even higher), which may provide data that are difficult to compare with physiological conditions (see also above). Generally, complexes with macrocyclic chelators dissociate following proton- or OH^–^-assisted mechanisms, while complexes with acyclic ligands often show contributions from metal-assisted pathways (see Figure 8).

**Figure 8:**
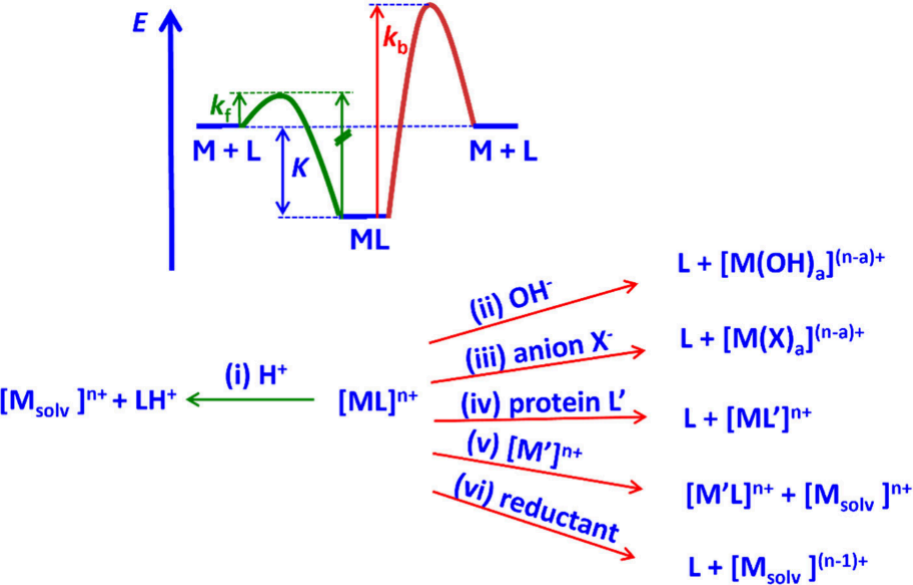
Qualitative potential energy surface (PES) and pathways for the complexation and decomplexation of radiometals.

The possible pathways and a qualitative energy diagram for complexation and decomplexation are listed in Figure 8. Preconditions for radiopharmaceuticals are fast complexation and inertness. From the potential energy surface (PES) on the top of Figure 8 it follows that, with a given complex stability, increasing the complexation rate leads to increased lability (green curve). Avoidance of microscopic reversibility, i.e., blocking protonation of the coordinated donors—green pathway (i)—is the key option to prevent lability with chelators that are efficiently radiolabeled.^229^ Steric shielding and rigidity have been shown to be possible ways to achieve this.^229^ Note that the complexation obviously follows a complex pathway that often is less well understood than in the seminal work of Cu^2+^-cyclam mentioned above;^225^ i.e., the M + L → ML pathway in Figure 8 (top) is an oversimplification. Importantly, the corresponding trajectory must, in general, also be available for decomplexation. That is, high complex stability obviously enforces slow decomplexation (see PES in Figure 8). In addition, steric factors may at least partially prevent microscopic reversibility. One possibility involves rigid pendant arms such as picolinates attached to coordinating amines as, e.g., shown in the examples discussed in Figures 3, 10 and 11: protonation of the corresponding carboxylate of these planar tetradentate units does not allow the rigid group to swing out easily, and this is discussed in detail elsewhere.^229^ A similar possibility is the stabilization of the chair–chair conformation in bispidines, specifically when the metal ion is coordinated (see Figure 11),^227,229^ and this has been shown to lead to extremely slow decomplexation and to decomplexation kinetics that do not show any H^+^ dependence.^206,229^ Attack of the radionuclide by OH^–^ or other anions [paths (ii) and (iii))] or by other ligands, specifically by abundant proteins [path (iv)], may be prevented by full encapsulation of the metal ion and rigid ligand scaffolds, where cleavage of a single metal-donor bond is not possible as for example with planar bi- or tridentate chelate units.^229^ Transmetalation (v) is similar to reprotonation (i) and may be hindered by efficient encapsulation and rigidity. Reduction of the metal center is mainly of relevance with Cu^2+^, where highly negative redox potentials are achieved with high complex stability since there is a linear correlation of redox potentials and Cu^2+^ complex stabilities due to only relatively small variations in the Cu^+^ complex stabilities,^233–235^ and this therefore is a case where complex stability and inertness are directly correlated. Cyclic voltammetry data obtained with Cu^2+^ complexes of macrocyclic ligands indicate that reduction potentials falling outside the window of reducing agents present *in vivo*, together with quasi-reversible voltammograms, correlate with a superior *in vivo* stability.^236–238^

Time-dependent spectrophotometry, also including stopped-flow experiments, is often used to determine reaction kinetics; some details on appropriate software packages for fitting are provided in the Supporting Information. Dissociation kinetics studies are generally conducted at variable pH to assess the proton- and base-assisted dissociation pathways. These studies require conditions in which the complex dissociates thermodynamically, and thus large excesses of acid, base, and/or scavengers need to be used. Ligand exchange reactions in compounds of radiopharmaceutical interest can also be investigated by NMR techniques. For instance, ^17^O and ^99^Tc NMR measurements were used to study ligand exchange reactions in *fac*-[(CO)_3_Tc­(H_2_O)_3_]^+^, an important precursor for the preparation of ^99m^Tc-based radiopharmaceuticals.^239,240^

As mentioned previously, the dissociation of macrocyclic ligand complexes often follows the acid- or base-catalyzed mechanisms and thus pseudo-first-order conditions are ensured by the large excess of acid/base required to induce dissociation.^241–244^ Complex dissociation may involve the formation of mono- or diprotonated intermediates, as illustrated in Figure 9 for lanthanide complexes. The metal-assisted mechanism does not play any role for most macrocyclic ligand complexes, although some exceptions have been reported.^245^ Spectrophotometry is often used to follow the reaction if the chelator incorporates chromophores, such as pyridyl or other aromatic units. Metal ions such as Cu^2+^ may also be used as scavengers, allowing the use of d–d transitions to follow the dissociation reactions. The terms that relate the observed rate constants *k*_obs_ with proton and Cu^2+^ concentrations are shown in Figure 9. This equation can be obtained by considering the definitions of the equilibrium constants shown in Figure 9 and expressing the total amount of complex present in solution as the sum of the concentrations of the different reactive species.^246^ Additional terms can be added to consider a base-catalyzed mechanism in case this plays a significant role (i.e., Bi^3+^ complexes).^247^

**Figure 9:**
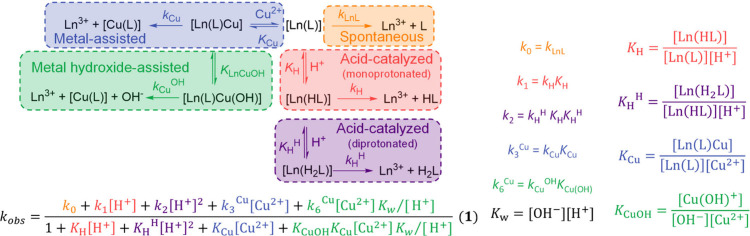
Representative dissociation mechanisms of Ln^3+^ complexes and the expressions required to fit the dissociation kinetics data.

Figure 10 shows examples of both macrocyclic and acyclic ligand complexes of different rare-earth ions that dissociate through different representative mechanisms. Examples are included, in which the same ligand binds to different metal ions, the same metal ion is complexed by different but structurally related ligands, and an example in which both the metal ion and chelator are different. In all cases, the observed rate constants *k*_obs_ increase with the proton concentration. However, the dependence on proton concentration changes depending on the nature of the ligand and the lanthanide ion. The dissociation of [Y­(PY3ABn)] shows a linear dependence with [H^+^], indicating that only the *k*_1_[H^+^] term in the nominator of the equation in Figures 9and 10contributes to *k*_obs_, with the *K*_H_[H^+^] term in the denominator being negligible (*K*_H_[H^+^] ≪ 1). The [Eu­(do3apic)]^−^ complex shows slightly different behavior, as the plot of *k*_obs_ versus [H^+^] displays a quadratic dependence. This indicates that the proton-assisted pathway proceeds through the formation of both mono- and diprotonated species, characterized by the rate constants *k*_1_ and *k*_2_, respectively. In contrast, the data obtained for the Ce^3+^ and Yb^3+^ analogues show a saturation behavior indicating that the *K*_H_[H^+^] term is not negligible, and *K*_H_ can thus be obtained from the fits of the kinetic data. Of note, all curves obtained for the complexes with macrocyclic chelators show negligible intercepts with the *y*-axis, indicating that the spontaneous dissociation, characterized by *k*_0_, does not contribute under the conditions employed in the kinetic studies.

**Figure 10:**
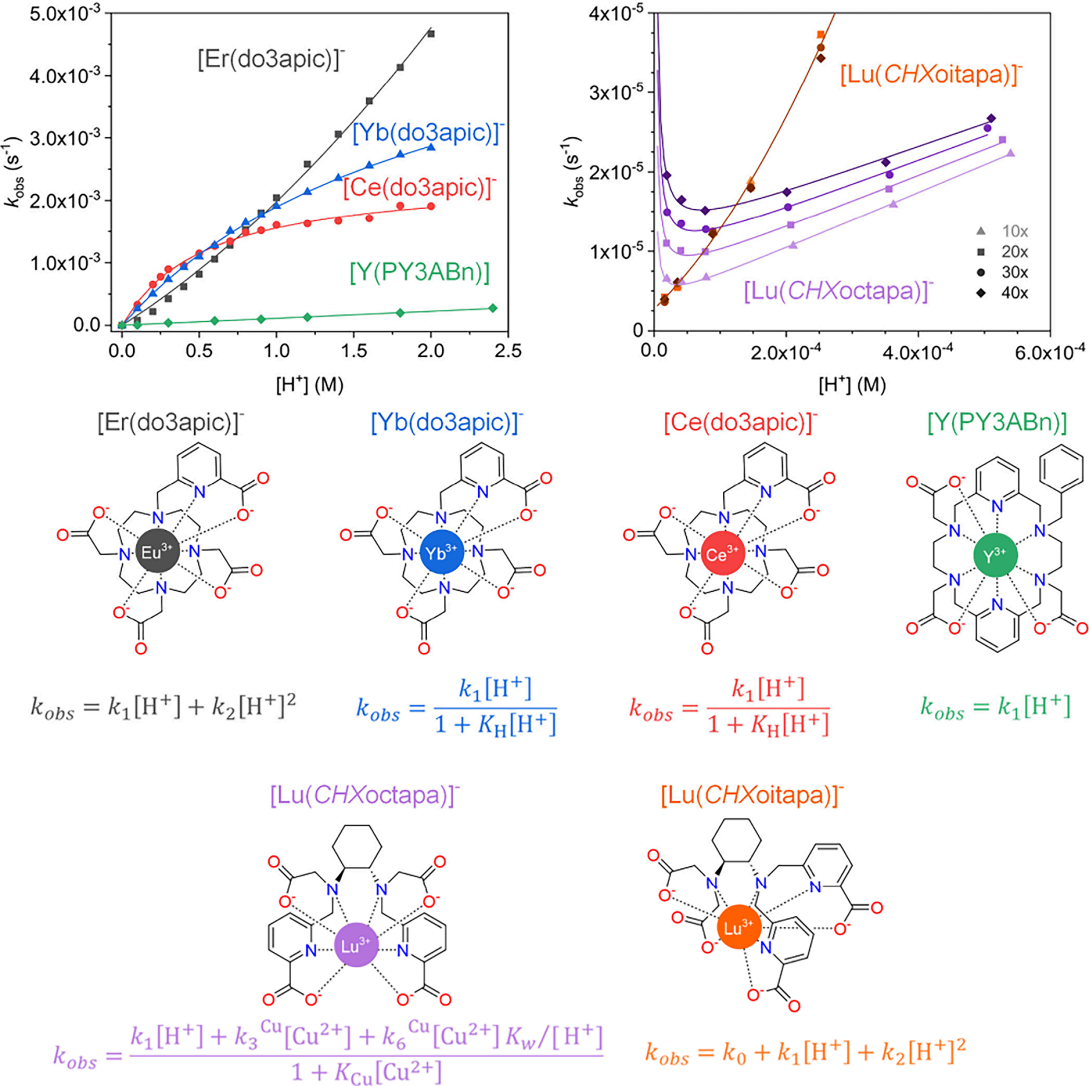
Representative dissociation kinetics studies on complexes with macrocyclic and acyclic ligands and the expressions used to fit the experimental data.

The complexes of acyclic octapa derivatives shown in Figure 10 display markedly different behavior when compared with the macrocyclic systems. Indeed, the dissociation of the [Lu­(CHXoitapa)]^−^ and [Lu­(CHXoctapa)]^−^ complexes requires relatively low proton concentrations, indicating that they are more labile than the complexes with macrocyclic ligands.^248,249^ The dissociation kinetics were monitored using a large excess of Cu^2+^ as a scavenger (Figure 10). The values of *k*_obs_ obtained for [Lu­(CHXoitapa)]^−^ show a quadratic dependence with [H^+^] and a non-negligible y-intercept, which indicates that both the spontaneous and proton-assisted mechanisms contribute to complex dissociation, the latter through the formation of mono- and diprotonated forms. The rate constants are not affected within experimental error when different Cu^2+^ concentrations are used, showing that the metal-assisted mechanism does not play a significant role. The situation is different for [Lu­(CHXoctapa)]^−^, which displays a linear dependence of *k*_obs_ versus [H^+^] when [H^+^] > 10^–4^ M and a strong dependence of the dissociation rates with [Cu^2+^], indicating that the metal-assisted mechanism provides an efficient pathway for complex dissociation (characterized by *k*_3_^Cu^).^250^ A similar behavior was observed for complexes of other acyclic chelators such as DTPA.^251^ The rate constants also increase at low proton concentrations, which indicates that dissociation may take place through a metal-hydroxido mechanism characterized by *k*_6_^Cu^. This pathway contributes significantly to the dissociation of the complex close to physiological pH.

The rate constants shown in Table 4 indicate that the values of *k*_1_ are more than 1 order of magnitude higher for the complexes of the acyclic chelators compared to the macrocyclic ones.^252^ Furthermore, the metal-assisted pathway contributes significantly at pH 7.4 for the complexes with some acyclic chelators. As a result, the half-lives calculated using the rate constants at pH 7.4 are significantly shorter for complexes with acyclic chelators. The data reported for the two acyclic chelators highlight the key role of ligand topology in preventing or favoring certain dissociation pathways.

**Table 4:** Rate Constants Characterizing the Dissociation of Ln^3+^ Complexes with Representative Macrocyclic and Acyclic Chelators and Half-Lives Calculated at pH 7.4^a^

	**[Ce(do3apic)]** ^**–**^	**[Eu(do3apic)]** ^**–**^	**[Yb(do3apic)]** ^**–**^	**[Y(PY3ABn)]**	**[Lu(CHXoctapa)]** ^**–**^	**[Lu(CHXoitapa)]** ^**–**^	**[Gd(DTPA)]** ^**–**^
*k*_0_/s^–1^	—	—	—	—	—	2.7 × 10^–6^	0.58
*k*_1_/M^–1^ s^–1^	2.40 × 10^–3^	1.56 × 10^–3^	5.9 × 10^–3^	1.13 × 10^–4^	0.0374	0.080	9.7 × 10^4^
*k*_2_/M^–2^ s^–1^	—	4.8 × 10^–4^	—	—	—	203	
*k*_3_^Cu^/M^–1^ s^–1^	—	—	—	—	6.3 × 10^–4^	—	0.93
*k*_6_^Cu^/M^–2^ s^–1^	—	—	—	—	5.1 × 10^5^	—	
*K*_H_	1.84		0.47		—	—	100
*K*_Cu_	—	—	—		12.1	—	13
*t*_1/2_/h^a^	6.2 × 10^5^	9.5 × 10^5^	2.5 × 10^5^	1.3 × 10^7^	876	69	203
ref	^249^	^249^	^249^	^252^	^250^	^248^	^251^

a Half-lives calculated as ln2/*k*_obs_ at pH 7.4 and [Cu^2+^] = 1 μM.

Overall, the examples provided evidence that both the nature of the ligand and the metal ion may affect the dissociation kinetics significantly. For transition metal complexes, dissociation kinetics vary dramatically depending on the specific characteristics of the metal ion, oxidation state, and electron configuration. This variability is well represented by the ligand exchange rates of aqua-complexes. For instance, water exchange in Cu^2+^_(aq)_ is very fast (*k* ^298^ ∼ 4 × 10^9^ s^–1^), while water exchange in Ni^2+^_(aq)_ is 5 orders of magnitude slower (*k* ^298^ ∼ 3 × 10^4^ s^–1^).^253,254^ The importance of the oxidation state is most obvious in the classical example of cobalt coordination chemistry. Cobalt isotopes, for example, are produced in oxidation state +2, the stable oxidation state for the aqua ion. However, since Co^2+^ complexes are labile, these are oxidized to stable and inert Co^3+^ complexes.^147^

## 6 Computational Modeling

Computational methods, preferably in combination with experimental data that may confirm the accuracy of the modeling, can help design completely new ligands, permutate on existing chelates, or interpret experimental, thermodynamic, or kinetic data. Data that allow to tune and confirm the computational models are experimental solution and solid-state structures, thermodynamic stability, kinetic data of similar metal ion/ligand systems, as well as spectroscopic data of the complexes or trapped intermediates, including electronic transitions, EPR and NMR parameters.

Computational methods include empirical force-field based methods for structural modeling,^255–258^ the computation of relative energies, e.g., in the context of conformational flexibility and cavity size and shape,^206,227,259^ and molecular dynamics and Monte Carlo based methods for searching the conformational space.^257,260^ Ligand-field-based methods are used for the computation of spectroscopic properties based on single crystal X-ray or computed structures (e.g., by force field calculations; e.g., d–d transition, EPR spin Hamiltonian parameters),^261,262^ and these methods have also been used to determine structures in solution.^263,264^ Quantum-chemistry-based approaches (often DFT) are used to compute properties related to the metal–ligand bonding,^265^ and this includes structural aspects as well as spectroscopic parameters (e.g., NMR, EPR, Mössbauer),^266–268^ stabilities (e.g., using energy decomposition analysis, EDA)^206,269–271^ and chemical transformations (complexation/decomplexation, i.e., the computation of transition states for various possible pathways). Obviously, combinations of the various methods mentioned may also be of relevance, and these are not discussed here in detail. The choice of method depends on the problem to be solved (it is a misconception to believe that the highest possible “level of theory” yields the most relevant results; “standard B3LYP DFT” is not always a reliable and relevant approach). In this context, it is of importance to remember that the accuracy of the description of a bond depends on the DFT functional used and the basis set and that this may be different for each metal ion/ligand donor system. Besides the functional and basis set, additional considerations such as integration grids, dispersion corrections, and the incorporation of solvent and relativistic effects must be considered. A guide to best practice in DFT studies has been published recently.^272^ Some specific systems and/or certain properties may not be well described by single-reference electronic structure methods, requiring the use of wave function methods, often based on the complete active space self-consisting field (CASSCF) method. However, this is very expensive and various approximations are available (e.g., DLPNO, NEVPT2)—obviously with corresponding limitations.^273,274^ These methods have been used to compute optical and various other spectroscopic and magnetic properties of transition-metal, rare-earth and p-block complexes.^268,275,276^

For any computational work, it is advisible to check and report the level of relevance and accuracy to be expected by a comparison with experimental data. What is required in any computational work is to report the type of approach used together with the software (including version) and all parameters used for each specific computation. For DFT e.g., this includes functional, basis sets, integration grids, and solvation model, and for molecular mechanics, e.g., the type of functions, minimization method, and force field parameter set. The information provided must enable the reader to reproduce the reported data. Moreover, for each computational study published, it is required to give the reader access to the computed coordinates to enable them to plot the molecule (in analogy to CSD data for experimental X-ray structures) or use them for further computational work and to understand the reported interpretation.

Figure 11 shows an example of a DFT-based computational study in combination with corresponding X-ray single crystal structural data.^206^ Here, hepta- and octadentate ligands were used to enforce Mn^2+^ selectivity with a Δlog *K* (Mn^2+^/Zn^2+^) of the order of 10.^206,277^ This study also involved molecular-mechanics-based cavity size and shape analyses as well as EDA calculations (not discussed here). The overlay plots in the middle row of Figure 11 show excellent agreement between experimental (red) and computed (blue) structures. This indicates that the theoretical models used (functional and basis sets) are appropriate. Importantly, this also indicates that the structures in solution are very similar but the coordinated triflate (OTf^–^) will be replaced by water in aqueous solution, leading to an efficient MRI contrast agent.^278^ Interestingly, the DFT analysis reveals that the Zn^2+^ complex with L^2^ has various, close to degenerate minima (bottom row in Figure 11), indicating that the Zn^2+^ structure is highly dynamic.

**Figure 11:**
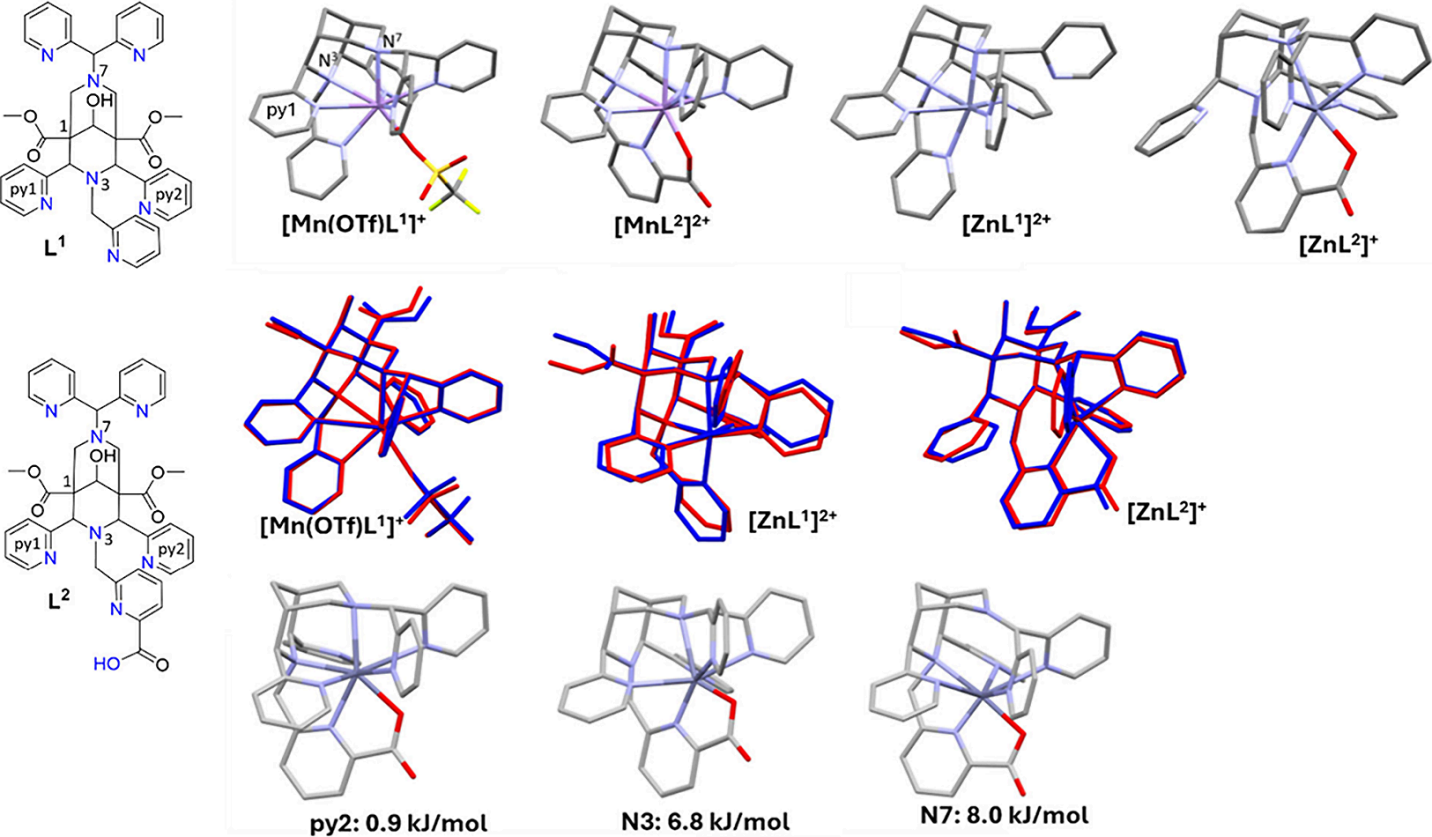
Experimental and DFT-computed structural data of Mn^2+^ and Zn^2+^ complexes of the hepta- (L^1^) and octadentate (L^2^) bispidines shown as ChemDraw structures (H atoms are omitted, and in the top and bottom rows substituents to the bispidine scaffold are also omitted). The top row shows the crystal structures, overlay plots of experimental (red) and DFT optimized (blue) structures appear in the middle row, and the bottom row shows three local minima of the hexa-/hepta-coordinate Zn^II^ complexes of L^2^.^206^

## 7 Radiochemical Labeling Protocols, Analysis and Interpretation of Results

Once the nonradioactive precursor and macroscopic chelate are appropriately characterized, radiochemical experiments can be conducted. This requires appropriate preparation of the radiopharmaceutical precursor (ligand) and the identification of analytical methods that allow monitoring and quantifying of the formation of the desired radiometal complex.

### 7.1 Preparing Stock Solutions of Ligand Precursor

As discussed in section 3, a host of chemical characterization techniques inform on the purity of precursors. Most techniques, however, do not appropriately capture the presence and quantity of inorganic salts which can comprise a large weight fraction of lyophilized and dried solids, unless such salts have quantifiable spectroscopic handles. Elemental analysis is not a suitable quantitation method for chemical constructs that require >10 synthetic steps and can only be synthesized at a sub-10 mg scale at which many disease targeting chelator-conjugates are prepared.

Therefore, other methods are better suited to determining the absolute quantity of chelator in a sample. If analytical instrumentation is available to determine absolute metal ion content in solution, such as Atomic Absorption Spectroscopy (AAS), Inductively Coupled Plasma Optical Emission Spectroscopy/Mass Spectrometry (ICP-OES/MS), a stock solution of metal ion with a known concentration should be first prepared and subsequently diluted to appropriate levels for accurate analysis. This metal ion solution is subsequently used to titrate a stock solution of ligand, monitoring conversion from ligand to complex by UV–vis spectroscopy or UV-HPLC. Once the saturation concentration is established, the corresponding concentration of ligand can be determined, in addition to the ligand’s molar absorptivity. With the molar absorptivity known, the concentration of the ligand can be readily determined from different synthetic batches. Figure 12 provides an exemplary data set for a Cu^2+^-batch titration of a stock solution of the NOTA chelator.^279^ Due to the absence of ligand chromophores above 220 nm, the n–d transition of the corresponding copper complex at 270 nm is monitored. A plot of the relative absorbance provides a means to determine the equivalence point and directly determines ligand stock concentration. The metal ion stock concentration should be at least 1 order of magnitude less than the estimated ligand concentration, and titration should be conducted to at least 2× the equivalence point/concentration to ascertain that sufficient data points were acquired. Attention must be paid to the relative size match of the titrant and ligand to account for the formation of additional species besides 1:1, which can result in sloping of the saturation section of the titration; furthermore, the rate of complexation should be rapid and occur within the time intervals used to add aliquots of titrand.

**Figure 12:**
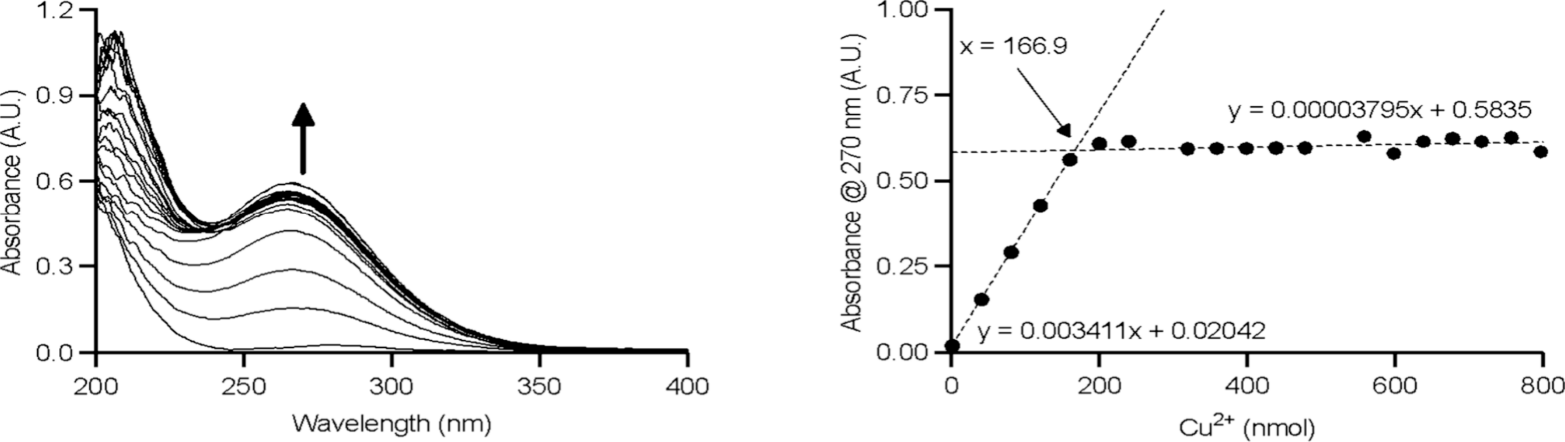
Sample ligand titration of NOTA with Cu^2+^ stock solution as monitored by UV–vis. Plotting of the absorbance value against Cu^2+^ stock concentration determines the ligand stock concentrations; linear regression analysis of both sections of the titration curve determines the intersection point; reproduced with permission from ref 279. Copyright 2020, ACS Publications.

In the absence of analytical equipment that can directly quantify the metal ion content, metal ion stock solutions may be titrated with a commercially available ligand with a known extinction coefficient first. An example is 3-(2-arsono-phenylazo)-4,5-dihydroxy-2,7-naphthalenedisulfonic acid (arsenazo­(III)), a promiscuous chromophore with the ability to generate a spectroscopic response that can be readily monitored using UV–vis.^280^ We defer the reader to relevant literature detailing typical titration protocols.^281,282^

Following confirmation of single species nature of the ligand precursor, the formation and characterization of the nonradioactive, macroscopic analogue of the target radiometal complex followed by purity analysis LC-MS is required (NMR if the species is diamagnetic). Specifically, this sample should be used to establish the characteristic retention times or *R*_f_ values of the target radiochemical complex species. Here, it is essential that the target species does not coelute with precursor or side-product species; this is common especially with hydrophilic, low *M*_w_ complexes that are not well retained on conventional reverse phase chromatography columns. In this case, it is recommended to test other chromatography methods, which improve the resolution of the radiochemical precursor or target complex species; increasing commercial availability of reverse phase column sorbents that have improved affinity and retention for hydrophilic compounds (“ultra-aqueous” C18) can be valuable for such analytical purposes. For more lipophilic compounds, solid phases should be adjusted accordingly.

### 7.2 RadioHPLC and RadioTLC Analysis—Key Guidelines for Positive Species Identification

Due to the subnanomolar quantities employed in radiochemical experimentation with medically relevant radionuclides, only a few analytical techniques provide insight into the speciation and identity of the corresponding radiochemical complex. By far, the most extensively used are chromatographic techniques where the radioactive species can be detected using gamma detector units (counts) or indirectly by autoradiography. Specifically, radioactive high performance liquid chromatography (radioHPLC) and radioactive thin-layer chromatography (radioTLC) are among the most extensively utilized in exploratory and clinical radiochemistry. The detection of a characteristic retention time, that is different from those produced by the reactive precursor and products of side reactions is required to successfully identify and detect the desired radiochemical species (*vide infra*).

Most radiochemical HPLC analyses are conducted using reverse-phase chromatography and pH buffered water–methanol/water–acetonitrile mobile phases. Suitable analysis methods are identified by optimizing the chromatographic behavior of nonradioactive, macroscopically characterized species. Due to the constraints of the nuclide’s half-life, retention times and chromatographic methods should be as short as possible, unless resolution does not permit shortened analysis. Another aspect of radioHPLC or radioTLC analysis is the optimization of activity quantities that provide a sufficiently strong signal output to not only detect the target species but also identify side-products with sufficient accuracy. This can be achieved by individual calibration experiments for each radionuclide of interest, using known quantities of radioactivity to determine the 1) limit of detection and 2) linear response range of the detector.

Similarly, radioTLC is frequently employed to track and confirm reaction progress; indeed, radioanalytical techniques for clinical batch release of [^18^F]-FDG have relied on radioTLC analysis to confirm identity and purity of the radiopharmaceutical.^283^ It is important to note that the use of radioTLC analysis is generally not sufficient for target compound identification, unless the *R*_f_ is well resolved and within 0.2–0.7 due to risks of coelution with nontarget species. However, it can serve as a means to rapidly evaluate and track reaction progress without the comparatively lengthy acquisition time of a radioHPLC chromatogram. The polarity of coordination complexes can render the selection of mobile phase conditions for normal solid phases such as aluminum oxide and aluminum backed silica especially challenging. Table 5 provides examples of TLC conditions that have been employed to monitor the radiolabeling of nonfunctionalized ^64^Cu, ^68^Ga, ^45^Ti coordination complexes and others. We note that functionalized/peptide-linked coordination complexes generally do not fall above an *R*_f_ of 0.05 and therefore should be additionally characterized for radiochemical purity, speciation, and complex identity by detection of characteristic retention time using radioHPLC.

**Table 5:** Selected Examples of RadioTLC Conditions for Assessing Radiometal-Chelator Stability and/or Radiolabeling Conversion Yield

**Radiometal**	**TLC plate type**	**Mobile phase**	**References**
[^203^Pb]Pb^2+^	iTLC-SA	EDTA (50 mM, pH 5)	^294^
[^227^Th]Th^4^	Al-backed SiO_2_ TLC	Citric acid (0.4 M, pH 4)	^148^
[^155/161^Tb]Tb^3+^	iTLC-SA	EDTA (50 mM, pH 7)	^148^
[^197m/g^Hg]Hg^2+^	iTLC-SG	DMSA (50 mM, pH 5)	^93^
[^64^Cu]Cu^2+^	iTLC-SG	EDTA (20 mM)/NH_4_OAc (0.15 M)	^295^
[^225^Ac]Ac^3+^	iTLC-SG	EDTA (50 mM, pH 5)	^296^
[^111^In]In^3+^, [^177^Lu]Lu^3+^	iTLC-SG	EDTA (50 mM, pH 5)	^297^
[^43/44/37^Sc]Sc^3+^	TLC-SG	25% aq. NH_3_/H_2_O/MeOH 2/1/1 (v/v)	^298^
[^132/135^La]La^3+^	iTLC-SG	Sodium citrate (0.4 M, pH 4)	^299^
[^68^Ga]Ga^3+^	iTLC-SC	EDTA (50 mM, pH 5.5)	^300^
[^45^Ti]Ti^4+^	iTLC-SG	Citric acid (0.1 M, pH 5)	^300^
[^213^Bi]Bi^3+^	iTLC-SG or SA	Citrate buffer (0.4 M, pH 4); EDTA (50 mM, pH 5.5)	^93^
[^89^Zr]Zr^4+^	iTLC-SG	DTPA (100 mM, pH 7)	^301^
[^45^Ti]Ti^4+^	iTLC-SG	Citric acid (0.1 M, pH 5)	^302^
[^44^Sc]Sc^3+^	Al-Silica	EDTA (50 mM)	^303^

Additionally, the identity of radiolabeled complexes should be confirmed by co-injection of a macroscopic amount of the corresponding nonradioactive metal complex (when available) with separately synthesized trace levels of the radiometal complex. The UV–vis chromatogram of the nonradioactive standard should align with the radiochromatogram of the radiolabeled tracer (Figure 13).^284^ This approach is especially important for small-molecule- or peptide-based radiopharmaceuticals, where radioTLC may not sufficiently resolve different isomers or species in solution.

**Figure 13:**
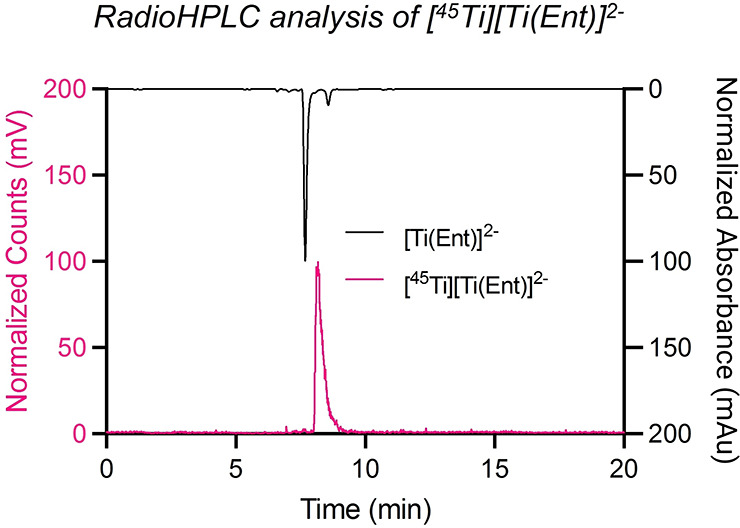
Representative HPLC chromatographic analysis of a co-injected sample of nonradioactive ^nat^Ti-complex (black) with radiolabeled ^45^Ti-complex (pink). Small peak offsets, as those seen above, are common and due to linear arrangement of detectors in the HPLC instrument setup. Image reproduced in part from ref 284. Copyright 2024, Wiley.

Radiochemical analysis by radioHPLC or radioTLC provides a means to quantify radiochemical conversion yields. While less common in conventional synthetic organic and inorganic chemistry, radiochemical conversion yields and radiochemical purity are generally reported as an average of experimental triplicates, including the standard deviation. This is an accepted standard because of the importance of reproducibility of radiochemical synthesis outcomes of radiopharmaceuticals in the clinic.^285^

### 7.3 Measurement of Apparent Molar Activity and Interpretation of Corresponding Experimental Data

The quantitation of apparent molar activity provides means to characterize the efficiency of radiochemical labeling methods to incorporate radioactive isotopes in the presence of defined quantities of nonradioactive precursor. Ideally, apparent molar activities should be maximized, meaning the ratio of the radioisotope to labeling precursor should be as close to 1:1 as possible, especially if the preparation of the radiopharmaceutical does not involve chromatographic purification to remove unreacted precursor. Otherwise, when administered *in vivo*, the unlabeled precursor may interact with the biological target and prevent the binding of the radiopharmaceutical, reducing the delivery of the radioactive payload to the disease target.^286^

For radiometal chemistry, due to high dilution conditions and presence of competing, nonradioactive trace metal ions in solution, a typical requirement is the need for at least 100-fold excess of the corresponding chelator precursor. The less selective and kinetically favored the chelation reaction, the larger the ligand loading required to achieve quantitative conversion to the desired radiochemical species must be. Apparent molar activity (abbreviated AMA) is typically determined by quantification of radiochemical yield in reactions using an identical activity quantity with varying quantities of chelator. The chelator concentration resulting in 50% radiochemical yield is multiplied by 2 to report the apparent molar activity in Ci/mol or Bq/mol. Concentrations (a minimum of 6) should be chosen such that the 50% yield is achieved, bracketed by at least one additional concentration measurement, and conducted in triplicate. Measurement of AMA in dependence of time provides an additional dimension of characterization to optimize radiochemical conversion yields. Clinical benchmarks provide guidance for desirable AMA values; a high AMA value not only guarantees optimized interaction with the biological target but also extends the “expiration time” of the radiopharmaceutical if it needs to be shipped over a greater distance or stored for next-day administration.

Accordingly, care must be taken when reporting and evaluating AMA values. AMA reduces as the radioactive isotope of interest decays and, therefore, is diluted in its native stock solution. This means that experiments conducted on “aged” radionuclide solutions will generally produce lower AMA values. Therefore, a control experiment is generally conducted and reported using a gold standard chelator such as DOTA (rare earth isotopes such as Sc, Y, La, Lu, and Ac, and large ionic radius main group metals such as In, Bi, and Pb), NOTA (late, first-row transition and main group metals such as Cu, Ga, and Mn), or DFO (early transition metals such as Ti and Zr and high-valent rare earths such as Ce and Th) to benchmark and contextualize radiolabeling performance appropriately. Figure 14 shows HPLC and TLC-supported analysis of an AMA measurement, including autoradiographic characterization in direct comparison, showing good agreement of quantitation with both results.^287^

**Figure 14:**
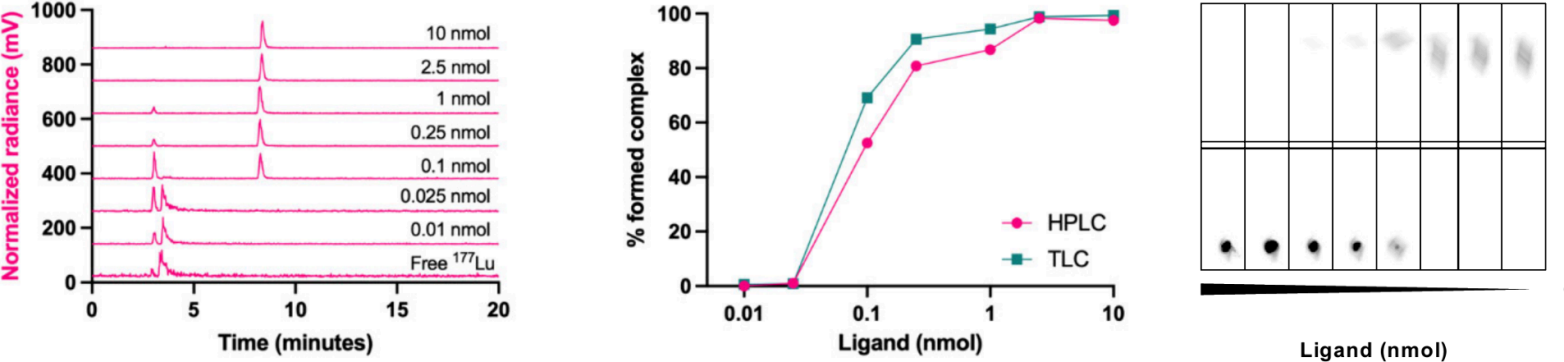
Ligand-quantity-dependent complexation of a macrocyclic phosphonate chelator with ^177^Lu (60 min, pH 5.5, 40 °C, *n* = 1). Comparison of low- and high-throughput quantification of apparent molar activity via radioHPLC (0.1% TFA/water and acetonitrile 16 min linear gradient) and radioTLC (mobile phase: 0.15 M ammonium acetate 10 mM EDTA, pH 5.0), respectively. Reproduced with permission from ref 287. Copyright 2024, ACS Publications.

### 7.4 Characterization of Radiolabeled Proteins: Gel Electrophoresis Autoradiography and Chromatography Techniques

While the characterization of radiolabeled small molecules and peptides (<5 kDa) is straightforward by radioTLC or radioHPLC due to their characteristic *R*_f_ and retention times, biologics are more challenging because they cannot be chromatographically separated from other labeled species without size-based resolution. A common approach is therefore to monitor radiochemical labeling by quantifying the fraction of protein-bound isotope versus free isotope using radioTLC.^288^ Generally, TLC methods will only separate macromolecular and small molecular fractions from one another and therefore provide no quantitative assessment or unequivocal identification of the desired chelate species. Therefore, it is recommended to conduct additional characterization to affirm that the isotope is coordinatively bound to a covalently protein-appended chelator. Specifically, size exclusion chromatography (i.e., size exclusion radioHPLC) and/or gel electrophoresis followed by autoradiography should be employed to confirm radiochemical labeling and monitor complex and protein integrity over time.^289–291^ While these methods are more time-consuming than radioTLC analysis, they provide superior analytical confirmation. An example is provided in Figure 15,^292^ where radiolabeled antibodies were characterized using autoradiography gel electrophoresis and size exclusion chromatography. Prior to radiolabeling, the antibodies (glycosylated or deglycosylated) had been modified either site-specifically and or stochastically with bifunctional chelators. Analysis using gel electrophoresis, employing reducing conditions, indicates where the radiochemical label is localized (heavy or light chain).^292^ The corresponding size-exclusion radioHPLC analysis reveals changes in speciation following exposure to plasma; of note, the small-molecular fraction frequently does not elute and has to be identified using other size exclusion methods or gel electrophoresis where the released activity is found on the bottom, low molecular weight region of the gel.

**Figure 15:**
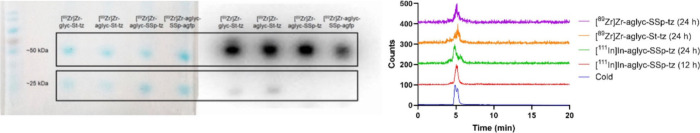
Gel electrophoresis (reducing conditions) of immunoconjugates radiolabeled with ^89^Zr, comparing Coomassie blue staining and autoradiography to identify the location of the radioactive tag (left). Size exclusion radioHPLC was used to characterize plasma stability of ^111^In and ^89^Zr-radiolabeled immunoconjugates (right). Reproduced with permission from ref 292. Copyright 2020, ACS Publications.

### 7.5 *In Vitro* Stability Assays: Chelator, Protein, and Plasma Challenge

To ensure that a radiopharmaceutical is both effective and safe, the radioactive drug must remain stable as it is transported to the target cells and eliminated from the body. The stability of a radiopharmaceutical *in vivo* is highly dependent on the chelator’s ability to securely bind the radiometal of interest and not “let go” of it for the duration of most decay events. Therefore, it is crucial that the radiometal-complex remains kinetically inert and does not undergo transchelation or transmetalation events when competing endogenous ligands and/or metal ions are encountered *in vivo*. To assess the kinetic inertness of radiometal-complexes before *in vivo* administration, *in vitro* tests are commonly performed. The tests discussed in this section offer valuable insights into the stability of the radiometal complexes; however, it is important to recognize that *in vitro* conditions do not accurately mimic the complexities of the *in vivo* environment. Cases have been reported where complexes exhibit high stability in serum *in vitro*, yet undergo rapid metabolism/dissociation *in vivo*.^293^

The following *in vitro* tests are commonly employed to assess the stability of radiometal-complexes, each providing distinct insights into their kinetic inertness: (i) serum/plasma stability, (ii) competition studies with endogenous metal-binding proteins, (iii) transmetalation, and (iv) transchelation studies. Generally, the radiometal-chelator complex is prepared using a standardized radiolabeling protocol at a concentration (10^–*x*^ where *x* = 3, 4, 5, 6 concentration of chelator or bioconjugate) determined to result in efficient radiometal complexation (i.e., RCY > 99%). Then, a solution containing the relevant biological medium (e.g., biological/endogenous ligands, metal ions, relevant chelators) is introduced to the solution containing the preformed radiometal-chelator complex. The mixture is incubated at an appropriate temperature (e.g., 37 °C) and pH (e.g., 7.4), typically with slight agitation. The stability of the complex is then monitored by removing small aliquots (5–10 μL) of the mixture at predetermined time points (e.g., 1 h, 3 h, 24 h, 48 h) that are analyzed to determine the quantity of radiometal displaced from the chelator over at least one radiological half-life. Common experimental techniques used to analyze the aliquots described previously include radioTLC, radioHPLC, gel electrophoresis, and/or PD-10 size exclusion chromatography. The choice of analytical method depends on the nature of the radiotracer being studied (e.g., small molecule, peptide, or antibodies). Each method provides insight into the percentage of the intact radiometal complex remaining at each selected time point. To ensure reproducibility, experiments are conducted in triplicate, and statistical analysis, including determination of standard deviations, is performed.

A negative control (*n* = 3) must be prepared in parallel to each *in vitro* test being conducted as outlined above, following the same protocol but without the addition of a chelator. Negative controls serve to validate the analysis by incubation of “free” radiometal with biological competitor (e.g., human serum) under equivalent conditions to ensure the radioanalytical technique can differentiate between intact radiometal-complex and uncomplexed “free” radiometal.

### 7.6 Chromatographic Methods to Separate and Identify Radiometal–Chelator Complexes

One way in which the aliquot of the competition incubated mixture described above (section 7.5) can be analyzed is by radioTLC (for commonly employed TLC methods, see Table 5). To analyze the mixture, the aliquot is spotted onto an iTLC plate, such as an iTLC-SA, iTLC-SG, or SiO_2_ TLC plate, depending on the radiometal used, where the TLC plate is typically 60–100 mm long. The iTLC plate is then developed in a chamber filled with an appropriate mobile phase.

Upon development, unbound radiometal migrates with the solvent front (*R*_f_ = 1) by forming a polyanionic complex with the excess chelating component of the mobile phase, while the intact complex remains near the baseline (*R*_f_ = 0–0.2). The percentage of intact complex is then determined using a radioTLC scanner. The radioTLC scanner (e.g., AR-2000, Eckert & Ziegler) is equipped with a radiation detector (e.g., gas-based radiation detector) sensitive to γ radiation and beta particles that moves along the plate and obtains measurements of emitted radiation as a function of distance on the plate as highlighted in Figure 16.^304^

**Figure 16:**
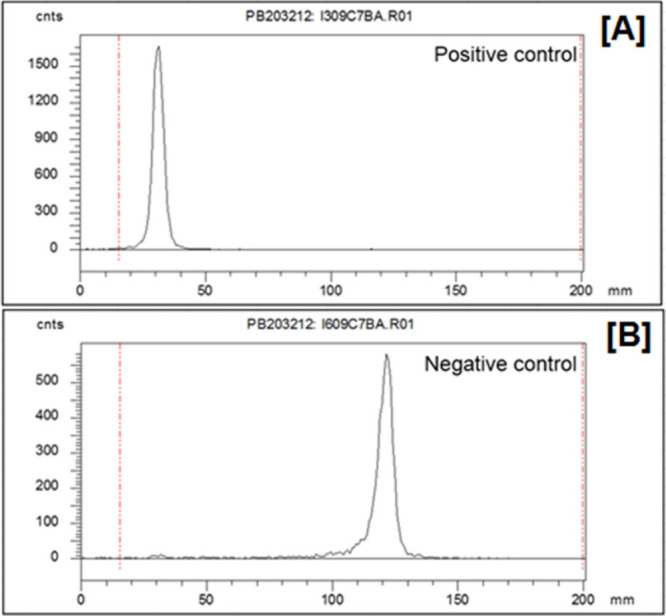
Representative positive and negative control iTLC radio-chromatograms for ^203^Pb radiolabeling. [A] 10^–4^ M DOTA-3Py labeled with ∼50 kBq ^203^Pb, [B] unlabeled ^203^Pb, at 1 h aliquot spotted onto SA iTLC plates, developed using EDTA (50 mM, pH 5.0) as the mobile phase.

In most instances, after the iTLC plate is developed, it can be immediately measured via radioTLC. For example, ^213^Bi plates should be read immediately to eliminate interference from the grow-in of grand-daughter ^209^Pb (*t*_1/2_ = 3.2 h). On the contrary, if the radioisotope being tested has multiple radioactive daughters (e.g., ^225^Ac, ^212^Pb, ^227^Th) it is critical to allow sufficient time for the daughter radioisotopes to reach secular/transient equilibrium before counting the iTLC plate. Premature measurement could result in misattributed activity, as daughter isotopes eluting with the solvent front may produce false positives, leading to inaccurate assessments of the RCY.

When the iTLC plate is ready for analysis by radioTLC, the scanning time required is determined by the activity level of the sample, with 0.5–3 min typically being sufficient to obtain an accurate reading.^305^ While radioTLC offers advantages, such as simplicity and rapid analysis, it also has limitations. For biological samples, appropriate sample preparation is essential, as direct spotting can otherwise result in false positives from components, such as serum proteins. Furthermore, degradation caused by radiolysis is often not detected by TLC; in such cases, the mixture should be analyzed using radioHPLC (see section 7.2).^306^

RadioHPLC is a sensitive technique capable of distinguishing between different radiolabeled species by detecting small changes in complex integrity, where the radioactivity detection is proportional to the concentration of the element or compound regardless of its chemical form in the sample. This method is particularly useful for low-molecular-weight radiometal chelators or radiometal peptides, as small structural changes can be detected through shifts in retention times. Its applications in radiopharmaceutical development are highly versatile, radioHPLC can even be used to track the metabolic fate of radiolabeled compounds in bacterial cultures or minute chemical changes to chelate structure by metabolic processing *in vivo*.^307^ RadioHPLC is further useful when the distinct separation of multiple compounds/components is required.^305^ For example, when trying to detect radiolysis events when performing *in vitro* studies, the aliquot of the serum-incubated mixture can also be analyzed by radioHPLC.^308^

Although radioHPLC is valuable for identifying transchelation, transmetalation, and degradation events, especially when monitoring radiometal-complex stability *in vitro*, it differs from radioTLC in that, when assessing complex integrity, the aliquot removed from the mixture cannot be immediately injected. Prior to analysis, proteins must first be precipitated using an organic solvent, for example, acetonitrile (1:1 v/v). Then the precipitate is separated from the supernatant by centrifugation, and the supernatant is collected, then diluted with water (preferably trace metal grade) before injection into the radioHPLC.^309^ A radiochromatogram is then obtained, where the area under each peak, as determined by radioHPLC analysis, defines the radiochemical purity (RCP). This enables the identification of radiolysis-induced/degradation products, as radiolysed peptides and/or any radiochemical impurities appear as distinct peaks on the chromatogram, separated from the nonlabeled complex.^310^ An example of minor chelating impurities in the formulation of a clinical radiopharmaceutical, [^177^Lu]­Lu-PSMA-617, is provided in Figure 17.^311^ Additionally, if the radiometal possesses multiple radioactive daughters (e.g., ^225^Ac), each fraction containing radioactive species should be collected, and radionuclides should be quantified via an appropriate radioanalytical technique such as High-Purity Germanium (HPGe) gamma spectroscopy, to avoid misinterpretation of results.

**Figure 17:**
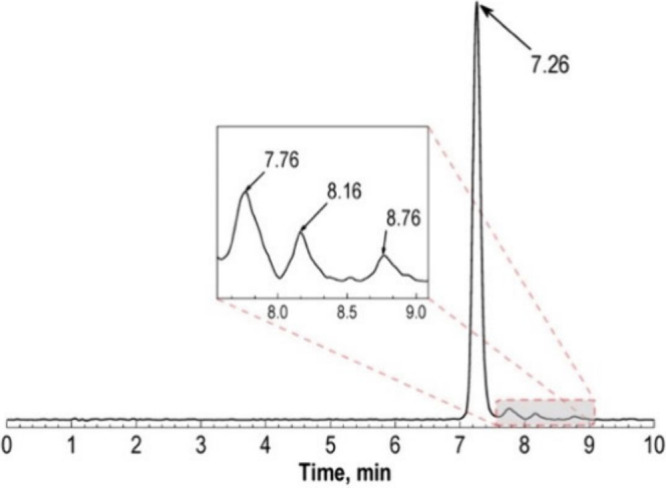
Radio-HPLC chromatogram of [^177^Lu]­Lu-PSMA-617; in addition to the main peak at 7.26, three radiochemical impurities at 7.76, 8.16, and 8.76 min were identified as products of structural changes in the PSMA-617 pharmacophore Glu-C­(O)-Lys. This figure demonstrates the ability of HPLC to identify small impurities that may have gone undetected via iTLC.^311^

A quick and relatively simple method capable of separating chelate-bound radiometal from large protein-bound radiometal (>5–10 kDa, e.g., serum-protein-bound) is provided by use of prepacked sepharose-based size exclusion columns, also termed PD10 columns (e.g., GE, Sephadex G25, size exclusion for MW < 5000 Da). These can be used to analyze the mixture by separating the components based on molecular weight.

The general procedure is as follows: at each set time, an aliquot is taken from the serum mixture, typically diluted with phosphate buffered saline (PBS, >1:5 v/v dilution), then counted with a well counter (e.g., Capintec CRC 15R) to obtain a value for total activity in the removed aliquot. Then, fractions containing serum-protein-bound radiometal are collected and analyzed using a gamma counter. The continued, fractioned elution can be conducted to capture low molecular weight components such as free radiometal and/or low molecular weight chelator-bound radiometal; in some instances, however, strong molecular interactions with the sepharose column material may preclude elution of all loaded activity. Each isolated fraction may be compared to the total activity sampled to quantify species in both fractions. By comparing the initial activity to the activity in the serum-protein bound fraction, researchers can determine the percentage of radiometal that is no longer chelate-bound.^312,313^ Note that this method does not provide information about protein aggregates or protein degradation; such information must be obtained using radioHPLC.

### 7.7 *In Vitro* Stability Assessment

The most common *in vitro* test conducted to assess the stability of radiometal-complexes is the (human/mouse) serum/plasma stability test. In this assay, preformed radiometal-chelator complexes are incubated with serum at physiological temperature and pH, and changes in complex integrity are monitored over time. Typically, serum/plasma is added directly to the vial containing the preformed radiometal complex, with a 1:1 (v/v) dilution being standard. However, a 1:10 (v/v) dilution or higher (e.g., 1:50 v/v) may be performed to better simulate the extreme dilution conditions encountered *in vivo*, providing a more thorough assessment of stability.^294,310^ The mixture is then incubated, and the displacement of radioactivity from the chelator to serum proteins is monitored over time using the techniques outlined above alone or in combination to quantify the extent of dissociation and complex degradation when incubated in serum and plasma *in vitro*. It is important to evaluate the stability of the radiometal-chelator complex not only in human serum/plasma but also in a variety of sources, such as in mouse and rat.^314^ Differences in endogenous metal-binding proteins across species can impact the stability of the complex, potentially leading to variations in transchelation events. Assessing stability across multiple serum types provides a more comprehensive understanding of the *in vitro* behavior of the complex.

A common dilemma researchers may encounter is whether to test radiometal-complexes in serum or plasma. To make the decision, one must first understand the differences between the two. Serum and plasma are both derived from blood, and contain elements of virtually all proteins produced in the body, but differ in composition due to the presence or absence of clotting factors.^315^ Plasma is the liquid component of blood, collected in a syringe (heparinized) precoated/filled with anticoagulant (e.g., heparin, EDTA, or citrate), preventing the blood from clotting. After collection, the sample is centrifuged to separate the plasma from the blood cells. However, the anticoagulants remain.^316^ Serum is similar to plasma in composition, but it lacks the clotting factors, as it is collected in a syringe (nonheparinized) without any anticoagulant (e.g., heparin), allowing the blood collected with it to clot naturally. After clot formation, the sample is centrifuged to remove fibrin clots, blood cells, and coagulation factors, leaving behind the serum. The choice between serum and plasma is dependent on the goal of the experiment. Serum is often preferred for biochemical assays, biomarker analysis, and immunoassays as clotting factors in plasma can sometimes interfere with the results of the experiment. However, studies have indicated that protein profiles obtained from plasma and serum can be very different; therefore, when it comes to testing radiometal-chelator complexes, the ideal solution for determining the stability would be to perform *in vitro* tests in both serum and plasma.^317^

Serum stability studies evaluate the integrity of the radiometal-chelator complex in a biologically relevant environment containing numerous competing ligands capable of displacing the radiometal. However, based on the coordination preferences of a radiometal, targeted competition assays can be designed to better predict the susceptibility of radiometal transchelation to specific biological chelators *in vivo*. In such experiments, the radiometal-complex integrity is challenged using an excess of a particular biological competitor. Refer to Table 6 for selected examples of *in vitro* stability competition studies with endogenous metal-binding proteins. Therefore, evaluating the stability of radiometal-chelator complexes in the presence of specific biologically relevant competitors enables identification of the most likely culprits responsible for transchelation *in vivo* (Table 6).

**Table 6:** Selected *In Vitro* Stability Tests of Radiometal-Chelator Complexes in the Presence of Biological Competitors

**Biological competitor**	**Radiometal of interest**	**Experimental conditions**	**Key observations**	**References**
l-Glutathione (GSH)	[^197m/g^Hg]Hg^2+^	50 mM l-glutathione (1:22 v/v GSH:reaction solution dilution, 37 °C).	Assess Hg^2+^ transchelation to thiol-containing biomolecules	^310^
Superoxide dismutase (SOD)	[^64/67^Cu]Cu^2+^	Presence of human SOD, physiological conditions.	Evaluate resistance of radiocopper complexes to copper transchelation	^318^
Cysteine	[^64^Cu]Cu^2+^	1000:1 Cys-to-ligand molar ratio.	Detect transchelation to thiol-containing biomolecules	^222^
Hydroxyapatite (bone mineral)	[^90^Y]Y^3+^, [^89^Zr]Zr^4+^	Incubate at a physiological pH	Determines affinity and retention of radiometal complexes targeting bone	^312^,^319^
Transferrin (apo-transferrin)	[^67^Ga]Ga^3+^	130-fold excess apo-transferrin, in the presence of bicarbonate at 37 °C	Assess likelihood of M^*n*+^ transchelation requiring chelator stability greater than M^*n*+^-transferrin complex	^320^
	[^111^In]In^3+^			
	[^45^Ti]Ti^4+^			

In addition to targeted metal ion sequestration protein challenges, transmetalation studies provide valuable insights into the kinetic inertness of radiometal-complexes by determining whether the chelator remains bound to the radiometal ion when exposed to competing metal ions or undergoes metal exchange. Generally, the preformed radiometal complex is prepared first with quantitative radiochemical purity (>99%) confirmed prior to incubating with an excess of nonradioactive metal ion. The competing, nonradioactive metal can be either the same ion (i.e., ^nat^Pb for ^203^Pb-labeled complexes), or an elemental surrogate ion if no nonradioactive isotopes exist (e.g., ^nat^La for ^225^Ac-labeled complexes), or another biologically relevant metal ion exists.

Competing metal concentrations typically range from 20-fold to several thousandfold. The chelator’s susceptibility to releasing the radiometal and binding to a nonradioactive metal is evaluated over time and analyzed as outlined above. Refer to Table 7 for selected examples of transmetalation *in vitro* studies.

**Table 7:** Selected Examples of Transmetallation Studies

**Radiometal**	**Stable competitor**	**Experimental conditions (molar excess compared to chelator)**	**References**
[^203^Pb]Pb^2+^	Pb^2+^	20-fold	^228^,^294^
[^89^Zr]Zr^4+^	Co^2+^, Cu^2+^, Fe^2+^, Ga^3+^, Gd^3+^, K^+^, Mg^2+^, Ni^2+^, Zn^2+^	10-fold	^321^
[^225^Ac]Ac^3+^	La^3+^	50-fold	^296^
[^47^Sc]Sc^3+^, [^45^Ti]Ti^4+^, [^68^Ga]Ga^3+^	Fe^2+^, Cu^2+^, Mg^2+^, Zn^2+^	100-fold	^300^
[^64^Cu]Cu^2+^	Zn^2+^, Ni^2+^	2-fold	^222^

Transchelation studies similar to those on the macroscopic scale described in section 5.1 are conducted to evaluate the resistance of a radiometal-chelator complex to displacement by excess competing ligands. Where a preformed radiometal chelator complex is prepared, the complex is then incubated with an excess (20–1000 fold) of a competing ligand (e.g., EDTA, DTPA, DOTA).^222^ Incubation conditions are consistent with serum/plasma and transmetalation assays. Table 8 summarizes selected examples of transchelation studies.

**Table 8:** Selected Examples of Transchelation Studies

**Challenge chelator**	**Radiometal**	**Competing ligand excess**	**Analytical method**	**References**
EDTA/DTPA	[^89^Zr]Zr^4+^	100–10000-fold excess	Radio-HPLC	^319^
EDTA	[^89^Zr]Zr^4+^, [^68^Ga]Ga^2+^ and [^177^Lu]Lu^3+^	100-fold excess	Radio-TLC	^321^
EDTA	[^203^Pb]Pb^2+^	20-fold excess	Radio-TLC	^294^
DOTA	[^64^Cu]Cu^2+^	1000-fold excess	Radio-HPLC	^222^
EDTA	[^64^Cu]Cu^2+^	100-fold excess	Radio-HPLC	^279^

An additional variable evaluated in these experiments is the pH dependence of transchelation susceptibility when radiometal-chelator complexes are challenged with competing ligands. Many chelators are pH sensitive; for example, for chelators containing amine N atoms, the coordinating nitrogen becomes protonated at a low pH, and as a consequence, its lone pair becomes unavailable for bonding with the metal ion, preventing/decreasing the likelihood of metal binding. Transchelation assays performed across a range of pH values (e.g., pH 4 to 7.4) help evaluate the complex’s stability under physiologically relevant conditions including those of acidic tumor microenvironments.^322^

In the same fashion as in the previously discussed studies at predetermined time points, aliquots are removed from the mixture and analyzed using radioTLC or radioHPLC to detect if the radiometal has been transferred from the original chelator to the challenging chelator. If the radiometal remains bound to the original chelator, the complex is stable under chelator challenge conditions (Figure 18).^319^

**Figure 18:**
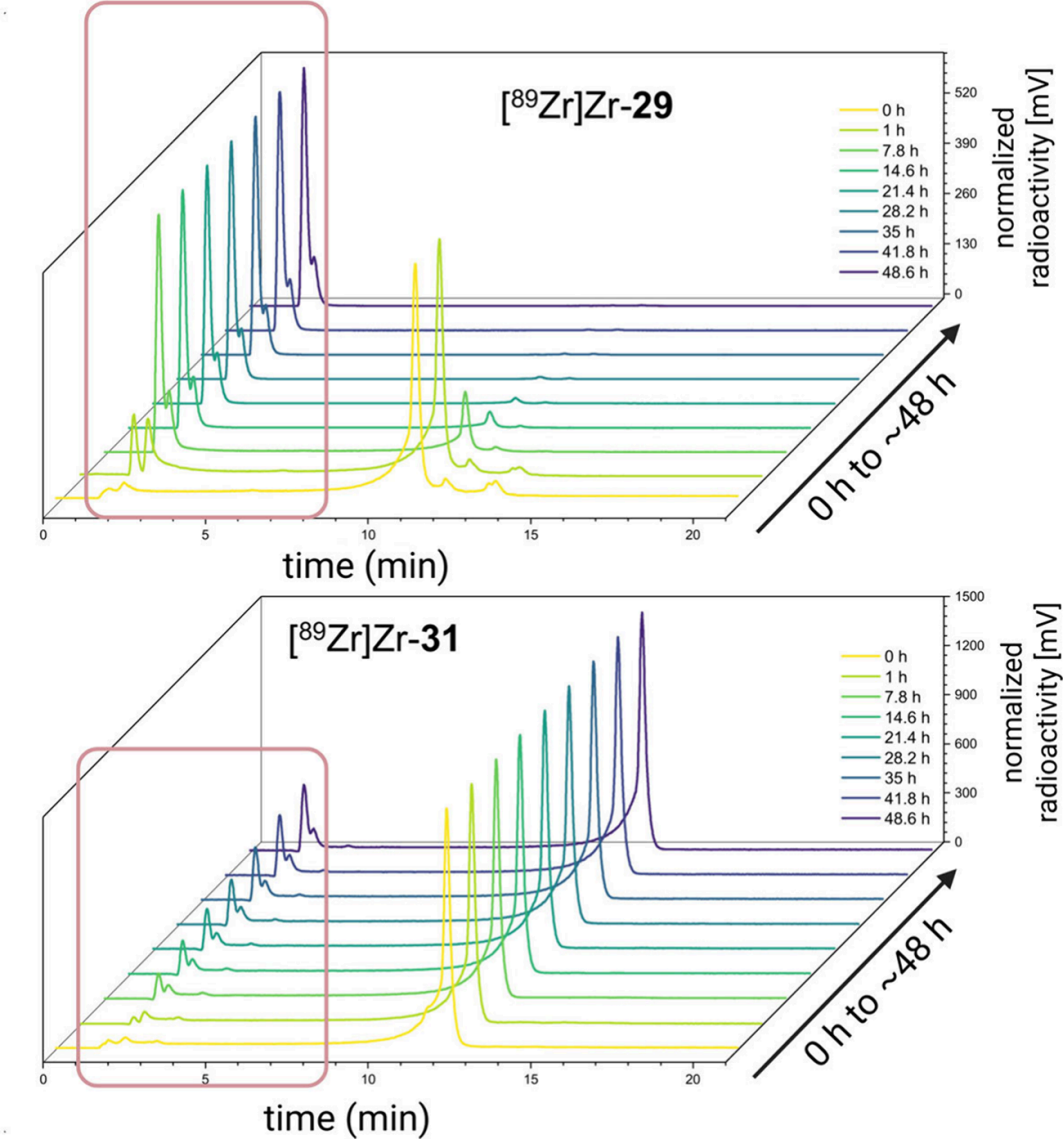
RadioHPLC analysis of various [^89^Zr]­Zr-labeled complexes (5 nmol complex) challenged with EDTA (50 μmol) and monitored over 48 h, illustrating differences in complex stability. The [^89^Zr]­Zr-**29** complex exhibits low inertness, undergoing immediate dissociation and rapid transchelation to EDTA, evidenced by rapid grow-in of the “free” radiometal ion peak and *t*_R_ < 5 min (red box) and disappearance of intact complex at *t*_R_ ∼11 min over time. In contrast, the [^89^Zr]­Zr-**31** complex is more inert, as evidenced by the slower grow-in of “free” radiometal ion peak and *t*_R_ < 5 min (red box) and maintained intact complex at *t*_R_ ∼12 min over 48 h, indicating strong resistance to challenge conditions. Reproduced in part with permission from ref 319. Copyright 2021, MDPI.

In the studies discussed above, a preformed radiometal complex was incubated with an external competitor to determine the likelihood of complex dissociation. While this approach assesses the kinetic inertness of the radiometal-complex, it fails to reveal the chelators’ inherent selectivity for the radiometal in the first place. To address this concern, competitive radiolabeling experiments can be performed by introducing nonradioactive metal ions (e.g., Na^+^, K^+^, Ca^2+^, Mg^2+^, Cu^2+^, Zn^2+^, Fe^2+^) to a solution containing the radioactive metal before adding the chelator. By adding the chelator to a mix of both stable metals and the radioactive metal, the selectivity of the chelator for a given radiometal can be examined.^323,324^ These studies can give insight into the need to obtain a radionuclide with high (radio)­chemical purity.

In the same fashion, competitive assays can be conducted by introducing an equimolar mixture of multiple chelators into a solution containing the radiometal, allowing for a direct comparison of their labeling efficiencies.^222^ These assays can provide insight into the radiometal-chelator complexes favored kinetically versus thermodynamically. The radiometal-chelator complexes formed first represent the “kinetic” species, while any subsequent changes over time indicate the formation of more thermodynamically stable complexes. Such changes can be monitored using radio-HPLC, which enables the detection of different radiolabeled species over time.

### 7.8 PBS and Shelf-Stability

In radiolabeling chemistry, buffer solutions are essential components of reaction mixtures. Common radiolabeling buffers include sodium acetate, ammonium acetate, phosphate buffered saline (PBS), 2-[4-(2-hydroxyethyl)­piperazin-1-yl]­ethanesulfonic acid (HEPES), 2-(N-morpholino)­ethanesulfonic acid (MES), and 2-amino-2-(hydroxymethyl)-1,3-propanediol (Tris-HCl).^325^ The buffer (PBS) serves multiple functions, including maintaining physiological ionic strength, diluting reaction mixtures, and acting as a wash buffer in size-exclusion chromatography, such as PD-10 column purification.^326^ Additionally, PBS is often employed as a nonbiological control to distinguish intrinsic chelator instability from biologically induced degradation. Stability assays in PBS are commonly conducted to assess the integrity of radiometal-chelator complexes over time, as degradation may occur due to radiolysis.^222^ A phenomenon could be attributed to the saline component of PBS (0.9% NaCl), which is frequently used in radiopharmaceutical formulations due to its isotonicity with blood. Despite this advantage, high NaCl concentrations are not ideal for storing radiometal-chelator solutions, as they can exacerbate water radiolysis, leading to the formation of free radicals that contribute to degradation.^311^

To mitigate these effects, high NaCl concentrations should be avoided during radiolabeling reactions if the respective radiometal-chelator complex is determined to be unstable overtime in PBS. Alternatively, chemical radioprotectants—radiolysis quenchers, e.g., ethanol 10% v/v or ascorbic acid 10% v/v—can be added to PBS incubation mixtures to evaluate their effectiveness in preventing degradation over time.^222,325^ Additional examples of commonly used radioprotectants are gentisic acid, l-methionine, and selenomethionine, all of which can be added before the addition of radiometal, or after radiotracer preparation.^325^ The selection of buffering conditions and the addition of radioprotectants are crucial for maintaining the stability of radiometal-chelator complexes over time.

Ultimately, the *in vitro* stability assays discussed in section 7.5, including serum/plasma stability tests, competition studies with endogenous metal-binding proteins, transmetalation, and transchelation assays, provide valuable insights into the kinetic inertness of radiometal-chelator complexes over time. Analytical techniques such as radioTLC, radioHPLC, SDS-PAGE, and PD-10 size exclusion chromatography serve as essential tools for quantifying the integrity of radiometal-chelator complexes over time when challenged *in vitro*. Understanding the interactions between endogenous chelators and metal ion competitors on radiometal-complex combinations is crucial for designing the next generation of stable and effective radiopharmaceuticals. Testing preformed radiometal-chelator complexes using a combination of the assays outlined above will enable radiochemists to better combat and prepare for potential issues that may arise *in vivo*. However, *in vitro* assays do not entirely replicate the complexity of the *in vivo* environment. Consequently, *in vitro* results must be interpreted with caution, and further *in vivo* testing must be conducted to confirm the suitability of the radiometal complexes for future clinical applications.

## 8 *In Vivo* Analysis

Preclinical studies play an important role in the development of chelators for metal-based radiopharmaceuticals, providing key insights into their stability under physiological conditions, an essential prerequisite to ensure reliable imaging or therapeutic performance once the chelator is conjugated to the tumor-targeting vector.

Biodistribution studies in animal models (typically healthy mice) are central to this evaluation as they offer information on the metabolic stability of the complex as well as clearance and excretion pathways. In a standard setup, animals are injected with the radiolabeled chelator, sacrificed at one or multiple time points (e.g., 1 h, 4 h, 24 h—depending on the half-life of the radioisotope of interest). Relevant organs—generally including the liver, kidneys, spleen, lungs, heart, intestines, brain, muscle, bladder, and representative bone sample—are collected to assess organ-specific retention. When feasible, these studies can be complemented or partially replaced by SPECT or PET imaging, which can provide a means to diminish excessive use of animals to study the time course of the radiolabeled compound distribution and excretion.

Blood samples are also collected to evaluate plasma or serum stability and protein binding, while urine and feces are analyzed to determine the excretion routes and identify potential radioactive metabolites. These analyses are typically conducted using radioHPLC or radioTLC techniques. HPLC analysis of blood components is generally conducted by the separation of soluble, reverse phase compatible components of blood metabolites. This involves separation of erythrocytes from plasma by centrifugation and isolation of soluble components of blood metabolites by precipitation of proteins by addition of cooled acetonitrile. The acetonitrile fraction is subsequently chromatographically analyzed. It is important to note that this provides insight into only the acetonitrile-soluble plasma metabolite fraction. The protein precipitate should be evaluated for residual radioactivity to account for protein associated radioisotope or radiochelate. Resolubilization of the protein fraction and analysis by size exclusion chromatography provides additional insight into the nature of the protein-associated radioactivity (*vide supra*, section 7). For HPLC analysis of urine metabolites, generally, no additional processing besides filtration is required prior to chromatographic analysis. Among radiochelation studies that investigate metabolites, a vast majority of studies include urine and blood analysis. Metabolite analysis from harvested organs is more involved and generally requires mechanical tissue dissociation prior to extraction with organic solvents, which can be destructive to the chelate or protein adducts.

Importantly, the *in vivo* behavior of radiometal complexes can vary significantly depending on their physicochemical properties. Highly stable, nonfunctionalized complexes are typically excreted rapidly—via the kidneys/bladder/urine for hydrophilic species or through the liver/feces for more lipophilic ones.^327^ Charged, lipophilic species are excreted enterohepatically in the gall bladder. In contrast, unstable complexes often exhibit persistent uptake in organs where the nonbound radiometal naturally accumulates.^328^ Therefore, retention in these organs may serve as an indicator of poor *in vivo* stability. To evaluate this, the biodistribution of the free, unchelated radiometal represents an essential benchmark, providing a baseline for evaluating the ability of the chelator to retain the radiometal under physiological conditions. Figure 19 illustrates representative biodistribution profiles for a variety of unchelated radiometals spanning the periodic table, including transition metal ions and p-block elements.

**Figure 19:**
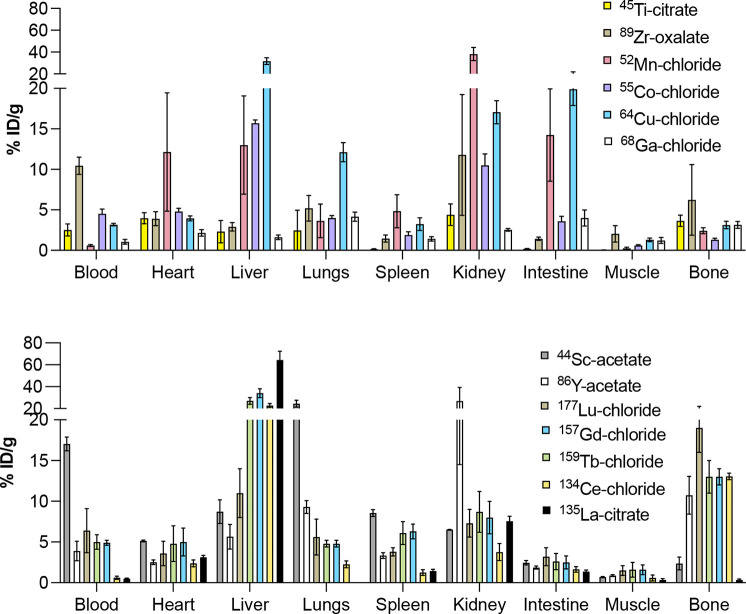
Biodistribution analysis of select isotopes at the 1 h time point post injection, analyzed by radioactivity quantitation (Ti, Zr, Mn, Co, Cu, Ga, Sc, Y, Lu, Ce, La) or inductively coupled plasma-mass spectrometry (Gd, Tb). Top: transition metals and p-block metals. Bottom: rare-earths. For numerical values, see the Supporting Information.

The biodistribution profile of nonchelated radiometals is generally governed by endogenous proteins that sequester the parent metal ion or a chemically homologous metal ion, then traffic and deposit the ion subsequently in tissues or organs of predominant storage or metabolism. For instance, the sequestration of ^64^Cu by ceruloplasmin and subsequent deposition in the liver have been thoroughly investigated;^329,330^ the chemical homology between Fe^3+^ and Ga^3+^ has been widely recognized and is considered the source of high blood retention of ^68/67^Ga by sequestration of transferrin.^331^ On the other hand, later transition metals that play essential functions as metallocofactors have established sequestration and deposition routes. For instance, Mn^2+^ shows homologies to Ca^2+^, which results in accumulation in the heart, and eventual capture by calprotectin, a divalent ion sequestration protein of the gut, mirrored by the high intestinal uptake of ^52^Mn.^332^ Co^2+^ may be captured in similar ways but ^55^Co biodistribution studies show more elevated levels in the liver, which represents the primary vitamin B12 (cobalamin) storage organ and biological Co^2+^ sink.^333^

The trend observed among the rare earth series is less straightforward to interpret and offers insight into divergent biological behavior that is closely linked to the charge-to-ionic radius ratio. Specifically, large lanthanides show significant accumulation in the liver (arising from sequestration by serum albumin), whereas smaller ionic radius rare earths also show increased deposition in the bone (a hallmark of increasing oxophilicity). Sc^3+^, the smallest rare earth, diverges from this trend by showing enhanced blood retention, possibly caused by scavenging by transferrin due to the Sc^3+^ ion’s small ionic radius.^287,334^ Ti^4+^ provides a strikingly similar biodistribution profile, supporting the hypothesis that Ti^4+^’s close chemical homology to Fe^3+^ is reflected by binding to transferrin.^335,336^ Due to its comparatively large ionic radius, Zr^4+^ has a diminished affinity to transferrin, resulting in rapid deposition in bone due to this ion’s oxophilicity and high relative charge^337^—the actinide Th^4+^ shows similar behavior.^338^ Only few data sets exist that describe the *in vivo* behavior of free actinide ions, which, in the case of Ac^3+^, shows both liver and bone uptake comparable to smaller rare earths.^339^ However, this behavior more likely arises due to the chemical homologies to Ra^2+^ and Ca^2+^, which are both effective bone-seeking ions.^340^ Table 9 provides a summary of the primary endogenous binders and associated organs of deposition for selected radiometals.

**Table 9:** Main Endogenous Binder and Organ of Deposition for Selected Radiometals

	**Radiometal ion**	Main natural/endogenous binder	**Main** organ(s) of deposition (1 h p.i.)	**References**
Alkaline earth radiometals	^131^Ba^2+^	Hydroxyapatite	Bone	^341^
	^223^Ra^2+^	Hydroxyapatite	Bone	^340^
Transition radiometals	^43/44/47^Sc^3+^	Transferrin	Many well-perfused organs	^287^,^334^
	^45^Ti^4+^	Transferrin	Blood; many well-perfused organs	^293^
	^89^Zr^4+^	Hydroxyapatite	Kidney	^337^
			Bone	
	^64^Cu^2+^	Ceruloplasmin (minor: superoxide dismutase)	Liver	^330^,^342^,^343^
	^52^Mn	Calprotectin, calcium channels	Intestine, heart	^332^
	^55^Co	Cobalamin (calprotectin)	Liver	^333^
	^197m/g^Hg^2+^	Metallothionein	Kidney	^344^
Radiolanthanides	^132/135^La^3+^	Serum albumin	Liver	^345^
	^134^Ce^3+^	Serum albumin	Liver	^339^
	^177^Lu^3+^	Hydroxyapatite serum albumin	Bone, liver	^287^
Radioactinides	^225^Ac^3+^	-	Liver	^339^
			Bone	
	^227^Th^3+^	Hydroxyapatite	Kidney	^338^
			Bone	
*p*-Block radiometals	^68^Ga^3+^	Transferrin	Many well-perfused organs	^331^
	^201^Tl^+^	Na^+^/K^+^-ATPase	Kidney	^346^

When feasible, benchmarking novel chelators against state-of-the-art systems (e.g., DOTA, NOTA, DFO, etc.) or comparison to validated literature data at identical sampling times and animal species is recommended.

However, it is important to note that radiometal complexes are typically small, highly polar, or charged molecules that may be cleared rapidly from circulation. Accelerated clearance means that these complexes do not persist in the body long enough to encounter biologically relevant challenges to their structural integrity. Conversely, more lipophilic complexes may exhibit prolonged retention in the liver and digestive tract, which could be misinterpreted as instability, even when the complex is intact. Notably, one of the first clinically translated radioactive coordination complexes, ^99m^Tc-sestamibi, exhibits characteristic uptake in the mitochondria-rich myocardium due to its similarity to the K^+^ ion,^347^ and TcO_4_^–^ localizes readily in the thyroid due to its similarity to the I^–^ ion.^348^ Therefore, although these preclinical studies are an essential first step, they may not fully predict the long-term *in vivo* performance of radiometal complexes once conjugated to long-circulating biomolecules, which is particularly pertinent for antibodies. Nonetheless, they provide a necessary foundation for advancing more comprehensive biological evaluations of radiochemical coordination complexes incorporating tumor-targeting vectors.

## 9 Concluding Remarks

The production, separation, chelation, and *in vitro* and *in vivo* validation of radiochelates require careful and thorough characterization of the coordination chemistry involved, requiring a multitude of analytical techniques to establish structural and electronic parameters that govern metal ion on/off kinetics and thermodynamic stability. These properties ultimately govern the chelate’s behavior in complex biological systems. While, to date, there are no computational or *in vitro* experimental approaches to reliably model and predict these properties, a combination of standardized approaches provides the required insight such that observed reactivity can be explored, interpreted, and modulated. The field of radiochelation chemistry has caught second wind, with work on established nuclides rapidly expanding and the exploration of preclinical radioisotopes with interesting properties for radiopharmaceutical applications also accelerating. This Review summarizes contemporary methods of characterization of ligand precursors, nonradioactive complexes and radiochemical complexes for established and yet to be synthesized radiochelates by radio- and coordination chemists, providing a consensus document for a growing scientific community.

## Supplementary Material



## Notes

NMR, HRMS, EA, HPLC, electronic absorption spectroscopy, vibrational spectroscopy (IR, Raman), EPR spectroscopy, X-ray and neutron diffraction methods, synchrotron methods (XAS, XES), electrochemical methods, other methods, recommended software packages for spectra simulations, thermodynamic and kinetic analysis, metal complex thermodynamic stability determination, computational analysis of metal complexes, and tabulated values for biodistribution data (PDF)
