# Supplementary material for: Chemical Tools to Characterize the Coordination Chemistry of Radionuclides for Radiopharmaceutical Applications
Source: Chem Rev. Author manuscript; Available in PMC 2026 Jan 16. (PMC12746417; doi:10.1021/acs.chemrev.5c00641)
Supplement: Supporting Info [file NIHMS2127299-supplement-Supporting_Info.pdf]

Supporting Information for:

Chemical Tools to Characterize the Coordination Chemistry of Radionuclides for Radiopharmaceutical Applications.

Eszter Boros,* Peter Comba, Jonathan W. Engle, Charlene Harriswangler, Suzanne E. Lapi, Jason S. Lewis, Simona Mastroianni, Liviu M. Mirica, Carlos Platas-Iglesias, Caterina F. Ramogida, Raphaël Tripier, Marianna Tosato.

Table of Contents

1. NMR 2
2. HRMS 2
3. EA 2
4. HPLC 2
5. Electronic Absorption Spectroscopy 3
6. Vibrational Spectroscopy (IR, Raman) 3
7. EPR Spectroscopy 3
8. X-ray and Neutron Diffraction Methods 4
9. Synchrotron Methods (XAS, XES) 4
10. Electrochemical Methods. 4
11. Other Methods. 5
12. Recommended Software Packages for Spectra Simulations, Thermodynamic and Kinetic Analysis 5
13. Metal Complex Thermodynamic Stability Determination 5
14. Computational Analysis of Metal Complexes 7
15. Tabulated values for Biodistribution Data 8

1. NMR Spectroscopy

A detailed list of the peaks observed should be included in the experimental section or supporting information, detailing the frequency of the spectrometer used, the solvent and temperature at which the experiments were carried out. Most journals require ^{1}H signals to be reported with a 0.01 ppm precision, and ^{13}C signals to be reported with a 0.1 ppm precision. Additionally, spectra recorded should be included, without suppressing any signals, in the SI of the publication. At least, ^{1}H and ^{13}C should be reported, although inclusion of 2D spectra is encouraged to assign the signals unequivocally. If there are any heteroatoms in the compound with useful NMR nuclei (^{19}F, ^{31}P or others), the signals and spectra should be reported as well. NMR spectra should present a good signal-to-noise ratio and should show only signals that can be assigned to the compound of interest, residual solvents, or modifiers such as TFA or formic acid, among others, which must be clearly identified in the spectra. The introduction of more user-friendly spectrometers operating at higher fields and the introduction of multi-pulse techniques and methods such as dynamic nuclear polarization (DNP) and signal amplification by reversible exchange (SABRE) that afford significant gains in sensitivity and of *in situ* methods has allowed NMR spectroscopy to rise to the challenges posed by modern science. Overall, NMR will remain a core spectroscopic technique for studying functional coordination chemistry.

2. HRMS

HRMS can be used as a criterion of compound identity but does not necessarily represent purity of the sample. The signals for which a molecular fragment can be determined should be reported in the experimental section and the spectra should be included in the SI. In well-justified cases, HRMS can substitute EA if the NMR is clean (i.e., very hygroscopic samples).

3. EA

Elemental analysis can be used to determine the percentage of different elements in a sample and is particularly useful for the quantification of salts present, as a final molecular formula can be worked out. The presence of salts can greatly affect the molecular weight, especially if the final purification step included TFA or formic acid in the mobile phases, which in turn can have a negative effect on radiolabeling yields. EA should be performed in duplicate at least, and an average of both analyses should be taken for the final composition. Also, the experimental and theoretical compositions are recommended by most journals to differ at most by 0.4%. However, there has been some debate on whether this threshold is adequate and it should be taken as a reference value.[1] Contrary to MS, which provides prove of identity, EA aids establishing the purity of the compound, and thus its use is strongly encouraged. If it is not possible to determine the elemental composition by EA, for example in the case where the final product is an oil, is very hydroscopic, or the amount of sample available is not sufficient, other techniques should be used. For instance, UV-Vis titrations with a metal ion that forms a 1:1 complex with the chelator can help determining the concentration of ligand, or the number of molecules of TFA or formic acid per molecule of chelator, thereby avoiding errors in the report of radiolabeling yields (see section 6-1). The amount of TFA can be also determined by techniques such as ^{19}F NMR and capillary electrophoresis.[2]

4. HPLC

The use of HPLC to determine the overall purity of the final compound intended for radiolabeling is essential if any of the previous techniques are not sufficient to prove the purity of the compound or if it was not possible to acquire EA, for example. To be considered pure enough for radiolabeling experiments, the purity by HPLC analysis should be at the very least of 95%, and the method used for the analysis should be reported including: column used, solvents, type of detector and the gradient used. As a recommendation, the use of a table to indicate the changes in the percentage of the solvents can be especially visual and helpful to replicate the analyses. Chromatograms should be included in the SI. The purification of the final chelators by preparative HPLC and the characterization by analytical HPLC often use TFA or formic acid in the mobile phase. The amount of acid present in the sample is difficult to quantify using this technique, and thus the use of alternative techniques like NMR or EA is strongly encouraged.

5. Electronic Absorption Spectroscopy

Absorption spectroscopy in the ultraviolet, visible, and near-infrared regions of the electromagnetic spectrum is one of the oldest and most often used characterization methods in coordination chemistry. A rich variety of colors are among the earliest features reported for coordination compounds, and they were studied by the very earliest versions of absorption spectroscopy in the visible range, involving the human eye as a detector. Over the course of the second half of the 20th century, absorption spectroscopy became one of the most used characterization techniques, leading to ubiquitous tables with wavelengths of absorption maxima and molar absorption coefficients. The absorption transitions of coordination compounds are molecular electronic excitations promoting the molecule from the ground state to an excited electronic state. In general, the energy minimum of the excited state is shifted along one or several normal coordinates compared to the ground state. These structure changes lead to broad absorption bands due to many non-zero Franck-Condon factors.[3] The absorption spectra of coordination compounds in the visible or NIR region are usually dictated by the energies of the d orbitals, and ligand field theory can be employed to predict the d-d transitions.[4] In addition, charge-transfer bands in the UV-vis region can provide information on the strength and nature of metal-ligand interactions.[5] Whenever possible, the solvent used for the measurements, the position of the absorption maxima, and molar absorptivity coefficients should be provided for a proper characterization.

6. Vibrational Spectroscopy (IR, Raman)

Vibrational spectroscopy is concerned with the measurement of the energies of transitions between quantized vibrational states of molecules, and these transitions usually occur in the middle infrared (IR) region of the electromagnetic spectrum, hence the commonly used term IR spectroscopy. IR spectroscopy is often used as a rapid analytical tool for the characterization of coordination complexes. Some of the more common uses of IR spectroscopy in coordination chemistry include: (i) detecting the presence of particular ligands (*e.g.*, CO, CN, *etc.*) and their mode of binding, *e.g.*, whether they are terminal or bridging; (ii) determining the mode of coordination of some ligands (*e.g.*, NO_2, N_2, or H_2); or some further structural information, using new subtle approaches; (iii) providing some information on bonding (electron distribution, oxidation state, *etc.*); and (iv) monitoring equilibria and following the progress of reactions. IR has also become a particularly powerful structural probe when it is used to monitor kinetic processes, from very slow to extraordinarily fast, in the latter case particularly for electron-transfer processes.[6] The interpretation of IR spectra of coordination compounds requires some understanding about the principles of vibrational spectroscopy, force constants, the use of symmetry and group theory, and knowledge of the frequencies (and intensities) of a wide range of functional groups.[7]

Raman spectroscopy is another spectroscopic method commonly used by inorganic chemists today to help in establishing the structures of the new molecules that have been synthesized. It is a complementary technique to infrared (IR) spectroscopy in that it also provides important information about the vibrations of the molecules in the gaseous, liquid, and solid states. The origins of these two molecular spectroscopic methods are, however, quite different. Infrared spectroscopy involves the absorption of electromagnetic (IR) radiation, while Raman spectroscopy is based on the scattering of electromagnetic radiation, usually in the visible region.[8] For all reported IR or Raman data, the phase of the measured samples should be reported (solid state, thin film, solution state, *etc.*), as well as the assignment of the essential transitions that are characteristic of the compounds of interest.

7. EPR Spectroscopy

Compared to NMR spectroscopy, electron paramagnetic resonance (EPR) spectroscopy is a less routine analytical tool in most synthetic laboratories but is critical tfor understanding the electronic structure of paramagnetic metal centers.[9] EPR, also known as electron spin resonance spectroscopy, provides detailed information about the electronic structure of metal centers with unpaired electrons and interactions with neighboring nuclear or electron spins.[10] EPR signals are characteristic of the metal, oxidation state, and spin state, and can be used to identify the presence of a particular species, and do so quantitatively.[11] Samples may be in fluid solution or solid state (powder, glass, single crystal), and it is important that the reported data include the solvent mixtures used as well as pH, electrolyte, concentration of the solution, the temperature at which the sample was prepared and at which the EPR spectra were collected, the exact frequency of the EPR spectrometer, and the spin integration of the EPR spectrum versus a known paramagnetic standard. Knowing the spin relaxation properties of a metal helps in the selection of probes of structure and function, and in interpretation of results obtained from other methods such as nuclear magnetic resonance, Mössbauer and electronic spectroscopy. Continuous wave (CW) and pulsed EPR methods, along with isotope substitution, are powerful tools for characterization of metal ions and their interactions with the surroundings. Reported spin Hamiltonian parameters generally are the result of spectra simulations, and these need

to be presented together with the software and method used. Finally, advances in molecular orbital calculations, primarily using density functional theory, as well as ligand field calculations, have dramatically enhanced the interpretation of experimental data.

8. X-ray and Neutron Diffraction Methods

Diffraction methods offer the most powerful and information-rich approach to chemical structure determination. Of these, the most widely used is single-crystal X-ray diffraction, but the application of the technique is broadened using neutrons and with microcrystalline powders instead of single crystal samples. The method, in use for over a century and being well established, continues to be developed. Characterization of a new compound is not complete without a single-crystal X-ray structure, and the rapidly expanding structural databases such as the Cambridge Structural Database (CSD) and the Inorganic Crystal Structure Database (ICSD) are proof of the prominence of X-ray diffraction as an analytical tool; more than half of the >1 million entries into the CSD contain one or more metallic elements. By comparison, the impact of neutron diffraction and powder diffraction methods is less pronounced compared with the overwhelmingly dominant single-crystal X-ray diffraction can be seen in the fact that these two less well represented techniques contribute approximately 0.2% and 0.5%, respectively, of the entries in the CSD, however, they offer distinct advantages in particular cases.[12] Characterization based on diffraction by crystalline samples provides information on composition and molecular connectivity, as do various spectroscopic methods, but they give full geometrical parameters, both intramolecular and in the spatial arrangements of molecules and/or ions in the solid state. Such detail is unachievable by other physical investigations; it is important for unambiguous and reliable characterization of newly synthesized molecules, and for a full understanding of observed physical and chemical properties. However, it is important to keep in mind that the structure obtained from single-crystal X-ray diffraction may not represent the actual structure in solution, especially given the known lability of coordination compounds and the ability of the ligands to rapidly exchange with solvent molecules (especially in aqueous environment), while more than one conformation or isomer could exist in solution at the same time.

9. Synchrotron Methods (XAS, XES)

The exploitation of synchrotron radiation to study structural and electronic aspects of coordination compounds has significantly developed during the past few decades.[13] The intense and tunable beams of X-rays produced by synchrotron radiation sources are used to study the crystal and molecular structures of coordination complexes that are too difficult to study by regular laboratory instruments. Among the methods used, X-ray absorption spectroscopy (XAS) includes measurements around absorption edges that allow the investigation of the atomic and electronic structures of complexes with elemental specificity, leading to insight into the atomic arrangement, oxidation states, chemical bonding and magnetic properties.[14] Because of the high intensity of synchrotron radiation, measurements can be rapid, leading to the investigation of samples undergoing physical or chemical change, induced by temperature, photochemical excitation, chemical reactivity and catalysis, adsorption of gas, and under *in situ* or *in operando* conditions.[15] As well as the X-ray sources themselves, the experimental techniques have continued to improve, along with the computational and theoretical means to model and analyze the data, and the interpretation of spectroscopic measurements.

10. Electrochemical Methods

Electrochemical techniques can be employed for a wide range of applications in coordination chemistry, such as defining the redox chemistry of coordination compounds, understanding the redox chemistry of metal centers of compounds of biological significance, electrosynthesis of compounds, electrochemical generation of unstable species in unusual oxidation states,the study of their reaction pathways, and obtaining thermodynamic or kinetic data.[16] The most commonly employed techniques used by coordination chemists undertaking electrochemical experiments are: 1) voltammetry under transient (*e.g.*, cyclic voltammetry) or steady-state (*e.g.*, rotated disk or microelectrode) conditions, which requires the interpretation of current–potential–time (I–E–t) curves; 2) spectroelectrochemical measurements in which a spectroscopic or other method of measurement (*e.g.*, mass spectrometry) is used in conjunction with electrochemistry to characterize intermediates or products of electrode processes; and 3) bulk electrolysis for the purpose of electrosynthesis or for a coulometric determination of the number of electrons associated with a redox reaction or a half-cell reaction.[17] Importantly, it is essential that such electrochemical methods are performed under conditions that are similar to those under which the coordination compounds are used for biological applications (*i.e.*, in aqueous conditions at the appropriate pH and electrolyte concentration). In addition, the electrode potentials need to be measured correctly and the appropriate refence potentials are

used when converting between various electrode potential scales, to allow for a direct comparison among results obtained by various research groups.[18]

11. Other Methods

Additional spectroscopic methods and molecular magnetism, not discussed here in detail, may yield structural and electronic or quantitative analytical information. The latter is the case when specific molecular parameters are obtained, as *e.g.* the extinction coefficients in UV-vis-NIR spectra, where the Lambert-Beer law allows to determine the concentration of a solution (*i.e.*, the purity of a compound), when the extinction coefficient is known. Another example is molecular magnetism, where magnetic moments are molecular parameters. With spin quantification, this is also possible for EPR spectroscopy. Spectroscopies not routinely used in the area of radiopharmaceutical chemistry and therefore not discussed here include Squid magnetometry (molecular magnetism), ESEEM, ENDOR, NQR, NRVS, CD, MCD, Mössbauer spectroscopies, various additional synchrotron and photophysical methods, and these are described in detail in the literature, specifically also in collections for bioinorganic chemistry.[19]

12. Recommended Software Packages for Spectra Simulations, Thermodynamic and Kinetic Analysis

As mentioned, the interpretation of experimental data in these studies relies heavily on advanced software for data fitting and equilibrium modeling. Here are some of the most commonly used software packages in the field:

Hyperquad: This software is widely used for potentiometric data analysis and is capable of handling systems with multiple equilibria, including those involving hydroxide species.[20]

HypNMR: This package is specifically designed for analyzing NMR titration data and can model complex systems with multiple protonation and metal-binding equilibria.[21]

Spectfit: This software is used for fitting UV-Vis absorption spectra and is particularly useful when multiple absorbing species are present in solution.

EPR specific software: For analyzing EPR data, various specialized programs such as WinEPR Simfonia,[22] XSophe,[23] and EasySpin[24] exist, depending on the complexity of the system and the parameters being studied. These programs typically focus on simulating g-values, hyperfine and superhyperfine splitting, *i.e.* general spin Hamiltonian parameters but, specifically for oligonuclear complexes, can also include structural information.

Analytical speciation diagrams: These are given thanks to the Glee software for both protonation and complexation experiments. The software allows also the easy calculation of the pM values once the pK values and experimental conditions are in hand.

Kinetic analysis: ReactLab.[25] To guarantee reproducibility and the possibility to directly compare the reported rates with those of other systems, in addition to experimental parameters and statistical analysis, the method of parameter fitting needs to be reported.

Spectra simulations: The analysis of spectroscopic data in general may yield important information on structural and electronic properties of the complexes studied. This may involve fitting of spectroscopic traces (as described above for EPR) or the simulation of spectra based on the computation of electronic parameters (*e.g.,* spin Hamiltonian parameters of EPR spectra) based on quantum-chemical or ligand field theory-based methods (see below).

13. Metal Complex Thermodynamic Stability Determination

When conducting thermodynamic studies, it is crucial to specify and control experimental conditions to ensure the reliability and reproducibility of the results. Ionic strength, temperature, and pH are parameters that must be carefully monitored, as they can significantly influence the equilibrium between metal ions and ligands in solution. Ionic strength affects the activity coefficients of ions, altering their apparent binding affinities, while temperature directly impacts the thermodynamics of binding. Unfortunately, the enthalpic and entropic contributions required to characterize the temperature dependence of the equilibrium are rarely reported in literature. Experiments are typically performed at a fixed ionic strength (e.g., 0.1 M $NaNO_3$, 0.1 M KCl, 0.15 M NaCl) and at a controlled temperature (25°C). Clearly defining and reporting these parameters is essential for accurate interpretation of data and comparison across studies. The definitions of all equilibrium constants used for thermodynamic studies must be provided, either in the main text of the manuscript or in the supporting information.

The typical process of determining thermodynamic constants begins with the determination of protonation constants for the ligand. This step is important as it provides a baseline understanding of the ligand's behavior in solution, such as how several protons can be bound and at what pH they dissociate. These constants are usually determined via potentiometry or NMR titrations (or, when possible, UV-vis titrations). Once the protonation constants are known, complexation studies can proceed. The thermodynamic stability constants (K_{ML}) can be subsequently determined once ligand protonation constants are known. However, the K_{ML} indicate the affinity of the fully deprotonated ligand for the concerned metal ion (see also, supporting information). Thus, establishing the speciation in solution requires considering the ligand protonation constants in the equilibrium model, as well as any complex protonation constants, and those characterizing the formation of hydroxo species, if relevant.

The protonation of a given ligand is generally characterized by the corresponding stepwise protonation constants (K_i) defined as in Eq (1), but some software packages such as Hyperquad use cumulative protonation constants (β_i, Eq (2)).

$$K_i = \frac{[\mathrm{H_iL}]}{[\mathrm{H_{i-1}L}][\mathrm{H^+}]} \qquad \text{Eq (1)}$$

$$\beta_i = \frac{[\mathrm{H_iL}]}{[\mathrm{L}][\mathrm{H^+}]^i} \qquad \text{Eq (2)}$$

Alternatively, the protonation of a ligand can be described by the acid dissociation constants expressed as pK_a (Eq (3)). Note that $log\beta_i = \sum_i logK_i$.

$$\mathrm{p}K_{a,i} = -logK_{a,i} = -log\frac{[\mathrm{H_{i-1}L}][\mathrm{H^+}]}{[\mathrm{H_iL}]} = log\frac{[\mathrm{H_iL}]}{[\mathrm{H_{i-1}L}][\mathrm{H^+}]} = logK_i \qquad \text{Eq (3)}$$

For the discussion of complex stabilities, the coordination chemistry community generally uses stepwise (log K_{ML}) or cumulative stability constants (logβ_{ML}). For multidentate chelators that completely encapsulate the metal ion forming 1:1 (M:L) species, individual (log K_{ML}) and cumulative (log β_{ML}) stability constants are identical:

$$K_{ML} = \frac{[\mathrm{ML}]}{[\mathrm{M}][\mathrm{L}]} \qquad \text{Eq (4)}$$

Additionally, metal complexes often form protonated species in solution, which are characterized by the corresponding stepwise or cumulative protonation constants:

$$K_{MH_iL} = \frac{[\mathrm{MH}_i\mathrm{L}]}{[\mathrm{MH}_{i-1}\mathrm{L}][\mathrm{H^+}]} \qquad \text{Eq (5)}$$

$$\beta_{MH_iL} = \frac{[\mathrm{MH}_i\mathrm{L}]}{[\mathrm{M}][\mathrm{L}][\mathrm{H^+}]^i} = \sum_i K_{MH_iL} \times K_{ML} \qquad \text{Eq (6)}$$

The formation of hydroxido complexes is also quite frequent for certain metal ions, particularly if they are small and possess a high positive charge (*e.g.*, Ga^{3+}). The formation of hydroxido species can be described by the corresponding stepwise and overall equilibrium constants:

$$K_{MLOH} = \frac{[\mathrm{ML}]}{[\mathrm{MLOH}][\mathrm{H^+}]} \qquad \text{Eq (7)}$$

$$\beta_{MLOH} = \frac{[\mathrm{MLOH}][\mathrm{H^+}]}{[\mathrm{M}][\mathrm{L}]} = \frac{K_{ML}}{K_{MLOH}} \qquad \text{Eq (8)}$$

The speciation in solution may require considering additional equilibrium constants, for instance those characterizing the hydrolysis of metal ions (*e.g.* for Ga^{3+}) or the formation of oligonuclear species. All relevant equilibrium constants must be defined in the main text of the manuscript or in the supporting information of any manuscript.

The biological, medicinal and pharmacological communities generally use dissociation constants K_D, *i.e.*, the inverse of K values used in coordination chemistry (K_D=1/K_{ML}), to describe complex stabilities. As discussed in the main text, conditional parameters are useful to quantify the complex stability at specific conditions (*e.g.*, ambient temperature, physiological pH, specific concentration of the chelator and metal ions), allowing for a comparison of the stabilities of different complexes. For these comparative purposes at a specific pH value, the comparison of K_{ML} (or K_D) values may be misleading if the basicities of the ligands being compared are significantly different. A particularly useful and widely used parameter is pM,[26] another is the competitivity index CI.[27] The latter is defined as the logarithm of the conditional stability constant under certain conditions (pH and concentration of competitors).

pM is often defined as using the amount of uncomplexed metal ion as pM = -log$[M]_{free}$ for certain conditions of pH and metal and ligand total concentration, often pH 7.4, [L] = 10^{-5} M and [M] = 10^{-6} M. With the latter definition, the

minimum value of pM is 6 (all metal ion uncomplexed). The value of pM can be calculated from the different equilibrium constants, such as those shown in Eq (1) – Eq (8), using dedicated software such as Hyss. Alternatively, the pM values can be determined by a competition batch titration method, using a ligand L' for which the pM value is known. This method relies on the fact that the difference in $\log K_{ML}$ values for ligands L and L' equals the difference in pM values (ΔpM) for the total concentrations used to define pM. Therefore, one can write:[28]

$$\log K_{ML} - \log K_{ML'} = \mathrm{pM}_{ML} - \mathrm{pM}_{ML'} = log\frac{[\mathrm{ML}]}{[\mathrm{ML'}]} + log\frac{[\mathrm{L'}]}{[\mathrm{L}]} \qquad \text{Eq (9)}$$

$$log\frac{[\mathrm{ML'}]}{[\mathrm{ML}]} = log\frac{[\mathrm{L'}]}{[\mathrm{L}]} - \Delta pM \qquad \text{Eq (10)}$$

Thus, a plot of $log\frac{[\mathrm{ML'}]}{[\mathrm{ML}]}$ should provide a straight line with slope 1, which provides directly $\Delta\mathrm{pM} = log\frac{[\mathrm{L'}]}{[\mathrm{L}]}$ when $log\frac{[\mathrm{ML'}]}{[\mathrm{ML}]} = 0$. These methods have been widely used when solubility issues prevent a full determination of the equilibrium constants (*i.e.* for some HOPO derivatives), or when the amount of sample available is limited.[29] However, we notice that some authors obtained pM values from log/log plots giving slopes that differ very significantly from 1, which put into question the accuracy of the reported pM values.[30] Whenever possible, pM values should be determined using the whole set of equilibrium constants. Alternatively, it can be obtained directly from a conditional stability constant determined at the pH of interest using an appropriate mass balance.

An important point is that thermodynamic (and kinetic) parameters need to be measured and reported at constant and well-defined conditions (solvent, temperature, pH, electrolyte, ionic strength). With known dependences (*e.g.*, enthalpic and entropic terms, pH titrations etc.), stabilities at different conditions may be compared, at least qualitatively, and there are reports of the determination of physical parameters at variable (but accurately known) conditions (*e.g.* variable pH or ionic strength), but these are not routine techniques.[31]

14. Computational Analysis of Metal Complexes

There are dedicated force fields for the optimization of metal complexes, and these allow for the fast optimization of their structures, also leading to relative stabilities (strain or steric energies) and, depending on the method used, to vibrational spectra.[32] It is useful to define "the force field" as the functions used to approximate the terms employed to define the total strain energy of a system and their parameterization, and changes related to the functional form or parameterization of any part will in principle lead to changes of any of the parameters. That is, modifications of well-established force fields or the use of published parameter sets in different software packages need to be done with care. Dedicated force fields for metal complexes include the MOMEC and LFMM force fields.[33] For quantum-chemical modeling of complexes used in radiopharmaceuticals, DFT is the standard method, and program packages used include Gaussian,[34] Turbomole,[35] ADF,[36] and ORCA.[37] The recent years have primarily seen massive developments of ORCA, with constant improvement in terms of versatility, speed and accuracyand it also includes QM-MM modules (*i.e.* force fields) and is free of charge for academic users. Specifically, this also includes methods for computational spectroscopy, with a range of methods available, including AI-LFT (ab initio ligand field theory).

15. Tabulated values for Biodistribution Data

A graphical representation and source references for this data can be found in section 8 in the main portion of the manuscript.

Table S1. 1-hour post injection biodistribution analysis data summary of transition metal ions and metalloids in mice (n=3-4, species: balb/C, Ncru, male or female).

Organ/tissue	^{45}Ti-citrate (% ID/g ± stdev)[38]	^{89}Zr-oxalate (% ID/g ± stdev)[39]	^{52}Mn-chloride (% ID/g ± stdev)[40]	^{64}Cu-chloride (% ID/g ± stdev)[41]	^{68}Ga-chloride (% ID/g ± stdev)[42]	^{55}Co-chloride (% ID/g ± stdev)[43]
Blood	2.52 ± 0.74	10.45 ± 1.06	0.6 ± 0.11	3.18 ± 0.16	1.06 ± 0.3	4.5 ± 0.6
Heart	3.97 ± 0.69	3.91 ± 0.86	12.14 ± 7.31	3.95 ± 0.3	2.15 ± 0.4	4.8 ± 0.4
Liver	2.31 ± 1.38	2.91 ± 0.52	13 ± 6.07	31.66 ± 3.06	1.61 ± 0.29	15.7 ± 0.4
Lungs	2.47 ± 2.5	5.19 ± 1.58	3.64 ± 2.08	12.12 ± 1.18	4.16 ± 0.57	4 ± 0.3
Spleen	0.15 ± 0.06	1.46 ± 0.44	4.83 ± 2.04	3.24 ± 0.78	1.42 ± 0.27	1.9 ± 0.4
Kidney	4.4 ± 1.33	11.78 ± 7.45	38.14 ± 6.06	17.06 ± 1.42	2.53 ± 0.16	10.5 ± 1.4
Intestine	0.17 ± 0.05	1.43 ± 0.2	14.23 ± 5.7	19.89 ± 1.99	4 ± 0.99	3.6 ± 0.6
Muscle	0.04 ± 0.01	2.03 ± 1.01	0.25 ± 0.13	1.3 ± 0.22	1.22 ± 0.36	0.6 ± 0.1
Bone	3.65 ± 0.7	6.23 ± 4.35	2.42 ± 0.36	3.13 ± 0.46	3.14 ± 0.42	1.3 ± 0.2

Table S2. 1-hour post injection biodistribution analysis data summary of lanthanide ions in mice (n=3-4, species: balb/C, Ncru, male or female).

Organ/tissue	^{44}Sc-acetate(% ID/g ± stdev)[44]	^{86}Y-acetate (% ID/g ± stdev)[44]	^{177}Lu-chloride (% ID/g ± stdev)[44]	^{159}Tb-chloride (% ID/g ± stdev)[45]	^{157}Gd-chloride (% ID/g ± stdev)[45]	^{134}Ce-chloride (% ID/g ± stdev)[46]
Blood	17.04 ± 0.85	3.9 ± 1.19	6.4 ± 2.7	5 ± 0.9	4.9 ± 0.3	0.61 ± 0.2
Heart	5.13 ± 0.11	2.51 ± 0.28	3.6 ± 1.5	4.8 ± 2.2	5 ± 1.7	2.38 ± 0.43
Liver	8.72 ± 1.46	5.65 ± 1.51	11 ± 3	27 ± 3	34 ± 4	22.79 ± 1.75
Lungs	24.51 ± 3.13	9.3 ± 0.79	5.6 ± 2.2	4.8 ± 0.4	4.8 ± 0.4	2.26 ± 0.41
Spleen	8.56 ± 0.43	3.34 ± 0.35	3.8 ± 0.5	6.1 ± 1.4	6.3 ± 0.9	1.26 ± 0.35
Kidney	6.53 ± 0.01	26.93 ± 12.43	7.3 ± 1.7	8.7 ± 2.5	8 ± 2	3.77 ± 1.05
Intestine	2.45 ± 0.27	1.87 ± 0.19	3.2 ± 1.1	2.6 ± 1	2.5 ± 0.8	1.64 ± 0.35
Muscle	0.7 ± 0.06	0.88 ± 0.11	1.5 ± 0.6	1.6 ± 0.9	1.6 ± 0.6	0.59 ± 0.36
Bone	2.36 ± 0.78	10.73 ± 2.32	19 ± 3	13 ± 2	13 ± 1	13.06 ± 0.4

Table S3. 1-hour post injection biodistribution analysis data summary of lanthanum in mice (n=4, species: balb/C, male).

Organ/tissue	^{132}La-acetate (% ID/g ± stdev)[47]
Blood	0.5 ± 0.07
Heart	3.13 ± 0.23
Liver	64.35 ± 8.12
Lungs	±
Spleen	1.45 ± 0.2
Kidney	7.55 ± 0.61
Intestine	1.38 ± 0.18
Muscle	0.35 ± 0.15
Bone	0.35 ± 0.1

References

(1) Kuveke, R. E. H.; Barwise, L.; van Ingen, Y.; Vashisth, K.; Roberts, N.; Chitnis, S. S.; Dutton, J. L.; Martin, C. D.; Melen, R. L. An International Study Evaluating Elemental Analysis. *ACS Cent. Sci.* **2022**, *8* (7), 855-863. DOI: 10.1021/acscentsci.2c00325. Brown, R. J. C. Correspondence on "An International Study Evaluating Elemental Analysis". *ACS Cent. Sci.* **2023**, *9* (4), 600-601. DOI: 10.1021/acscentsci.2c01484. Proctor, S.; Lovera, S.; Tomich, A.; Lavallo, V. Searching for the Truth: Elemental Analysis–A Powerful but Often Poorly Executed Technique. *ACS Cent. Sci.* **2022**, *8* (7), 874-876. DOI: 10.1021/acscentsci.2c00761.
(2) Little, M. J.; Aubry, N.; Beaudoin, M.-E.; Goudreau, N.; LaPlante, S. R. Quantifying trifluoroacetic acid as a counterion in drug discovery by 19F NMR and capillary electrophoresis. *J. Pharm. Biomed. Anal.* **2007**, *43* (4), 1324-1330. DOI: https://doi.org/10.1016/j.jpba.2006.10.039.
(3) Solomon, E. I.; Lever, A. B. P. *Inorganic electronic structure and spectroscopy*; Wiley, 1999.
(4) Figgis, B. Ch. 6: Ligand Field Theory. In *Comprehensive coordination chemistry*, Vol. 1; Pergamon Press, 1987; pp 213-280.
(5) Reber, C.; Beaulac, R. 2.22 - Optical (Electronic) Spectroscopy. In *Comprehensive Coordination Chemistry II*, McCleverty, J. A., Meyer, T. J. Eds.; Pergamon, 2003; pp 287-302.
(6) Grills, D. C.; Turner, J. J.; George, M. W. 2.7 - Time-resolved Infrared Spectroscopy. In *Comprehensive Coordination Chemistry II*, McCleverty, J. A., Meyer, T. J. Eds.; Pergamon, 2003; pp 91-101.
(7) Nakamoto, K. *Infrared and Raman spectra of inorganic and coordination compounds, part B: applications in coordination, organometallic, and bioinorganic chemistry*; John Wiley & Sons, 2009.
(8) Warner, S. D.; Butler, I. S. 2.8 - Raman and FT-Raman Spectroscopy. In *Comprehensive Coordination Chemistry II*, McCleverty, J. A., Meyer, T. J. Eds.; Pergamon, 2003; pp 103-112.
(9) Eaton, G. R.; Eaton, S. S. 2.03 - Electron Paramagnetic Resonance Spectroscopy. In *Comprehensive Coordination Chemistry III*, Constable, E. C., Parkin, G., Que Jr, L. Eds.; Elsevier, 2021; pp 44-59.
(10) Weil, J. A.; Bolton, J. R. *Electron paramagnetic resonance: elementary theory and practical applications*; John Wiley & Sons, 2007.
(11) Eaton, G. R.; Eaton, S. S.; Barr, D. P.; Weber, R. T. *Quantitative EPR*; Springer Science & Business Media, 2010.
(12) Clegg, W. 2.04 - X-Ray and Neutron Diffraction. In *Comprehensive Coordination Chemistry III*, Constable, E. C., Parkin, G., Que Jr, L. Eds.; Elsevier, 2021; pp 60-76.
(13) Garino, C.; Borfecchia, E.; Gobetto, R.; van Bokhoven, J. A.; Lamberti, C. Determination of the electronic and structural configuration of coordination compounds by synchrotron-radiation techniques. *Coord. Chem. Rev.* **2014**, *277-278*, 130-186. DOI: https://doi.org/10.1016/j.ccr.2014.03.027.
(14) Penner-Hahn, J. E. 2.13 - X-ray Absorption Spectroscopy. In *Comprehensive Coordination Chemistry II*, McCleverty, J. A., Meyer, T. J. Eds.; Pergamon, 2003; pp 159-186.
(15) Fitch, A. N. 2.10 - Synchrotron Methods. In *Comprehensive Coordination Chemistry III*, Constable, E. C., Parkin, G., Que Jr, L. Eds.; Elsevier, 2021; pp 160-182.
(16) Bard, A. J.; Faulkner, L. R. *Electrochemical Methods: Fundamentals and Applications*; Wiley, 2012.
(17) Bond, A. M. 2.15 - Electrochemistry: General Introduction. In *Comprehensive Coordination Chemistry II*, McCleverty, J. A., Meyer, T. J. Eds.; Pergamon, 2003; pp 197-222.
(18) Holze, R. 2.08 - Electrode Potentials: Conversion Scales and Calculations. In *Comprehensive Coordination Chemistry III*, Constable, E. C., Parkin, G., Que Jr, L. Eds.; Elsevier, 2021; pp 119-128.
(19) Drago, R. S. *Physical Methods for Chemists*; Saunders, 1992. Scott, R. A.; Lukehart, C. M. Applications of Physical Methods to Inorganic and Bioinorganic Chemistry. John Wiley & Sons: 2007. Que Jr., L. Physical Methods in Bioinorganic Chemistry – Spectroscopy and Magnetism. University Science Books: 2000. Bakac, A. Physical Inorganic Chemistry – Principles, Methods, and Models. Wiley & Sons: 2010. Crichton, R. R.; Louro, R. O. Practical Approaches to Biological Inorganic Chemistry. 2nd ed.; Elsevier: 2020.
(20) Gans, P.; Sabatini, A.; Vacca, A. Investigation of equilibria in solution. Determination of equilibrium constants with the HYPERQUAD suite of programs. *Talanta* **1996**, *43* (10), 1739-1753. DOI: https://doi.org/10.1016/0039-9140(96)01958-3. Gans, P.; Sabatini, A.; Vacca, A. Determination of equilibrium constants from spectrophotometric data obtained from solutions of known pH: The program pHab. *Ann. Chim.* **1999**, 45.
(21) Frassineti, C.; Ghelli, S.; Gans, P.; Sabatini, A.; Moruzzi, M. S.; Vacca, A. Nuclear Magnetic Resonance as a Tool for Determining Protonation Constants of Natural Polyprotic Bases in Solution. *Anal. Biochem.* **1995**, *231* (2), 374-382. DOI: https://doi.org/10.1006/abio.1995.9984. Frassineti, C.; Alderighi, L.; Gans, P.; Sabatini, A.; Vacca, A.; Ghelli, S. Determination of protonation constants of some fluorinated polyamines by means of 13C NMR data processed by the new computer program HypNMR2000. Protonation sequence in polyamines. *Anal. Bioanal. Chem.* **2003**, *376* (7), 1041-1052. DOI: 10.1007/s00216-003-2020-0.

(22) *WinEPR Simfonia*. Bruker, https://www.bruker.com/en/products-and-solutions/mr/epr-instruments/epr-software.html (accessed 16 March 2025).
(23) Hanson, G. R.; Gates, K. E.; Noble, C. J.; Griffin, M.; Mitchell, A.; Benson, S. XSophe-Sophe-XeprView®. A computer simulation software suite (v. 1.1.3) for the analysis of continuous wave EPR spectra. *J. Inorg. Biochem.* **2004**, *98* (5), 903-916. DOI: https://doi.org/10.1016/j.jinorgbio.2004.02.003.
(24) Stoll, S.; Schweiger, A. EasySpin, a comprehensive software package for spectral simulation and analysis in EPR. *J. Magn. Res.* **2006**, *178* (1), 42-55. DOI: https://doi.org/10.1016/j.jmr.2005.08.013.
(25) *ReactLab Software Tools*. https://jplusconsulting.com/, https://jplusconsulting.com/ (accessed 16 March 2025).
(26) Harris, W. R.; Carrano, C. J.; Cooper, S. R.; Sofen, S. R.; Avdeef, A. E.; McArdle, J. V.; Raymond, K. N. Coordination Chemistry of Microbial Iron Transport Compounds. 19. Stability Constants and Electrochemical Behavior of Ferric Enterobactin and Model Complexes. *J. Am. Chem. Soc.* **1979**, *101*, 6097-6104.
(27) Krężel, A.; Wójcik, J.; Maciejczyk, M.; Bal, W. May GSH and l-His contribute to intracellular binding of zinc? Thermodynamic and solution structural study of a ternary complex. *Chem. Commun.* **2003**, (6), 704-705, 10.1039/B300632H. DOI: 10.1039/B300632H. Jeżowska-Bojczuk, M.; Kaczmarek, P.; Bal, W.; Kasprzak, K. S. Coordination mode and oxidation susceptibility of nickel(II) complexes with 2′-deoxyguanosine 5′-monophosphate and l-histidine. *J. Inorg. Biochem.* **2004**, *98* (11), 1770-1777. DOI: https://doi.org/10.1016/j.jinorgbio.2004.08.002.
(28) Pierre, V. C.; Botta, M.; Aime, S.; Raymond, K. N. Substituent Effects on Gd(III)-Based MRI Contrast Agents: Optimizing the Stability and Selectivity of the Complex and the Number of Coordinated Water Molecules1. *Inorg. Chem.* **2006**, *45* (20), 8355-8364. DOI: 10.1021/ic061262q.
(29) Werner, E. J.; Kozhukh, J.; Botta, M.; Moore, E. G.; Avedano, S.; Aime, S.; Raymond, K. N. 1,2-Hydroxypyridonate/Terephthalamide Complexes of Gadolinium(III): Synthesis, Stability, Relaxivity, and Water Exchange Properties. *Inorg. Chem.* **2009**, *48* (1), 277-286. DOI: 10.1021/ic801730u. Doble, D. M. J.; Melchior, M.; O'Sullivan, B.; Siering, C.; Xu, J.; Pierre, V. C.; Raymond, K. N. Toward Optimized High-Relaxivity MRI Agents: The Effect of Ligand Basicity on the Thermodynamic Stability of Hexadentate Hydroxypyridonate/Catecholate Gadolinium(III) Complexes. *Inorg. Chem.* **2003**, *42* (16), 4930-4937. DOI: 10.1021/ic026240s.
(30) Phukan, B.; Patel, A. B.; Mukherjee, C. A water-soluble and water-coordinated Mn(ii) complex: synthesis, characterization and phantom MRI image study. *Dalton Trans.* **2015**, *44* (29), 12990-12994, 10.1039/C5DT01781E. DOI: 10.1039/C5DT01781E.
(31) Maeder, M.; Neuhold, Y.-M.; Puxty, G.; King, P. Analysis of reactions in aqueous solution at non-constant pH: no more buffers? *Phys. Chem. Chem. Phys.* **2003**, *5* (13), 2836-2841, 10.1039/B301488F. DOI: 10.1039/B301488F. Comba, P.; Gahan, L. R.; Hanson, G. R.; Maeder, M.; Westphal, M. Carbonic anhydrase activity of dinuclear CuII complexes with patellamide model ligands. *Dalton Trans.* **2014**, *43* (8), 3144-3152, 10.1039/C3DT53135J. DOI: 10.1039/C3DT53135J.
(32) Comba, P. 2.14 - Computational Coordination Chemistry. In *Comprehensive Coordination Chemistry III*, Constable, E. C., Parkin, G., Que Jr, L. Eds.; Elsevier, 2021; pp 241-255.
(33) Comba, P.; Ströhle, M.; Hambley, T. W. The Directionality of d-Orbitals and Molecular-Mechanics Calculations of Octahedral Transition-Metal Compounds. *Helvet. Chim. Act.* **1995**, *78* (8), 2042-2047. DOI: https://doi.org/10.1002/hlca.19950780812 (acccessed 2025/04/06). Deeth, R. J.; Fey, N.; Williams–Hubbard, B. DommiMOE: An implementation of ligand field molecular mechanics in the molecular operating environment. *J. Comp. Chem.* **2005**, *26* (2), 123-130. DOI: https://doi.org/10.1002/jcc.20137 (acccessed 2025/04/06). Deeth, R. J.; Anastasi, A.; Diedrich, C.; Randell, K. Molecular modelling for transition metal complexes: Dealing with d-electron effects. *Coord. Chem. Rev.* **2009**, *253* (5), 795-816. DOI: https://doi.org/10.1016/j.ccr.2008.06.018. Comba, P.; Hambley, T. W.; Martin, B. *Molecular Modeling of Inorganic Compounds*; John Wiley & Sons, 2009.
(34) Frisch, M. J. T., G. W.; Schlegel, H. B.; Scuseria, G. E.; Robb, M. A.; Cheeseman, J. R.; Scalmani, G.; Barone, V.; Petersson, G. A.; Nakatsuji, H.; Li, X.; Caricato, M.; Marenich, A. V.; Bloino, J.; Janesko, B. G.; Gomperts, R.; Mennucci, B.; Hratchian, H. P.; Ortiz, J. V.; Izmaylov, A. F.; Sonnenberg, J. L.; Williams-Young, D.; Ding, F.; Lipparini, F.; Egidi, F.; Goings, J.; Peng, B.; Petrone, A.; Henderson, T.; Ranasinghe, D.; Zakrzewski, V. G.; Gao, J.; Rega, N.; Zheng, G.; Liang, W.; Hada, M.; Ehara, M.; Toyota, K.; Fukuda, R.; Hasegawa, J.; Ishida, M.; Nakajima, T.; Honda, Y.; Kitao, O.; Nakai, H.; Vreven, T.; Throssell, K.; Montgomery, J. A., Jr.; Peralta, J. E.; Ogliaro, F.; Bearpark, M. J.; Heyd, J. J.; Brothers, E. N.; Kudin, K. N.; Staroverov, V. N.; Keith, T. A.; Kobayashi, R.; Normand, J.; Raghavachari, K.; Rendell, A. P.; Burant, J. C.; Iyengar, S. S.; Tomasi, J.; Cossi, M.; Millam, J. M.; Klene, M.; Adamo, C.; Cammi, R.; Ochterski, J. W.; Martin, R. L.; Morokuma, K.; Farkas, O.; Foresman, J. B.; Fox, D. J. Gaussian 16, Revision C.01. *Gaussian, Inc., Wallingford CT* **2016**.
(35) Franzke, Y. J.; Holzer, C.; Andersen, J. H.; Begušić, T.; Bruder, F.; Coriani, S.; Della Sala, F.; Fabiano, E.; Fedotov, D. A.; Fürst, S.; et al. TURBOMOLE: Today and Tomorrow. *J. Che. Theor. Comp.* **2023**, *19* (20), 6859-6890. DOI: 10.1021/acs.jctc.3c00347.

(36) te Velde, G.; Bickelhaupt, F. M.; Baerends, E. J.; Fonseca Guerra, C.; van Gisbergen, S. J. A.; Snijders, J. G.; Ziegler, T. Chemistry with ADF. *J. Comp. Chem.* **2001**, *22* (9), 931-967. DOI: https://doi.org/10.1002/jcc.1056 (acccessed 2025/03/15).
(37) Neese, F.; Wennmohs, F.; Becker, U.; Riplinger, C. The ORCA quantum chemistry program package. *J Chem Phys* **2020**, *152* (22), 224108. DOI: 10.1063/5.0004608 From NLM.
(38) Koller, A. J.; Saini, S.; Chaple, I. F.; Joaqui-Joaqui, M. A.; Paterson, B. M.; Ma, M. T.; Blower, P. J.; Pierre, V. C.; Robinson, J. R.; Lapi, S. E. A General Design Strategy Enabling the Synthesis of Hydrolysis-Resistant, Water-Stable Titanium (IV) Complexes. *Angew. Chem. Int. Ed.* **2022**, *61* (22), e202201211.
(39) Petrik, M.; Zhai, C.; Novy, Z.; Urbanek, L.; Haas, H.; Decristoforo, C. In Vitro and In Vivo Comparison of Selected Ga-68 and Zr-89 Labelled Siderophores. *Mol. Imaging Biol.* **2016**, *18* (3), 344-352, journal article. DOI: 10.1007/s11307-015-0897-6.
(40) Wooten, A. L.; Aweda, T. A.; Lewis, B. C.; Gross, R. B.; Lapi, S. E. Biodistribution and PET Imaging of pharmacokinetics of manganese in mice using Manganese-52. *PLoS One* **2017**, *12* (3), 1-14. DOI: 10.1371/journal.pone.0174351.
(41) Boswell, C. A.; Sun, X.; Niu, W.; Weisman, G. R.; Wong, E. H.; Rheingold, A. L.; Anderson, C. J. Comparative in Vivo Stability of Copper-64-Labeled Cross-Bridged and Conventional Tetraazamacrocyclic Complexes. *J. Med. Chem.* **2004**, *47* (6), 1465–1474.
(42) Waldron, B. P.; Parker, D.; Burchardt, C.; Yufit, D. S.; Zimny, M.; Roesch, F. Structure and stability of hexadentate complexes of ligands based on AAZTA for efficient PET labelling with gallium-68. *Chem. Commun.* **2013**, *49*, 579-581.
(43) Mastren, T.; Marquez, B. V.; Sultan, D. E.; Bollinger, E.; Eisenbeis, P.; Voller, T.; Lapi, S. E. Cyclotron production of high–specific activity 55Co and in vivo evaluation of the stability of 55Co metal-chelate-peptide complexes. *Mol. imaging* **2015**, *14* (10), 7290.2015. 00025.
(44) Whetter, J. N.; Śmiłowicz, D.; Becker, K. V.; Aluicio-Sarduy, E.; Kelderman, C. A. A.; Koller, A. J.; Glaser, O. M.; Marlin, A.; Ahn, S. H.; Kretowicz, M. N.; et al. Phosphonate-Based Aza-Macrocycle Ligands for Low-Temperature, Stable Chelation of Medicinally Relevant Rare Earth Radiometals and Radiofluorination. *J. Am. Chem. Soc.* **2024**, in press. DOI: 10.1021/jacs.4c11254.
(45) Wallimann, R. H.; Mehta, A.; Mapanao, A. K.; Köster, U.; Kneuer, R.; Schindler, P.; van der Meulen, N. P.; Schibli, R.; Müller, C. Preclinical comparison of (radio) lanthanides using mass spectrometry and nuclear imaging techniques: biodistribution of lanthanide-based tumor-targeting agents and lanthanides in ionic form. *Eur. J. Nucl. Med. Mol. Imaging* **2025**, *52* (4), 1370-1382.
(46) Bailey, T. A.; Mocko, V.; Shield, K. M.; An, D. D.; Akin, A. C.; Birnbaum, E. R.; Brugh, M.; Cooley, J. C.; Engle, J. W.; Fassbender, M. E. Developing the ^{134}Ce and ^{134}La pair as companion positron emission tomography diagnostic isotopes for ^{225}Ac and ^{227}Th radiotherapeutics. *Nature Chem.* **2021**, *13* (3), 284-289.
(47) Aluicio-Sarduy, E.; Thiele, N. A.; Martin, K. E.; Vaughn, B. A.; Devaraj, J.; Olson, A. P.; Barnhart, T. E.; Wilson, J. J.; Boros, E.; Engle, J. W. Establishing Radiolanthanum Chemistry for Targeted Nuclear Medicine Applications. *Chem. Eur. J.* **2020**, *26* (6), 1238-1242. DOI: https://doi.org/10.1002/chem.201905202 (acccessed 2025/03/15).
